# Supplementary material for: Hypothalamic CDK4 regulates thermogenesis by modulating sympathetic innervation of adipose tissues
Source: EMBO Rep. 2020 Jul 12;21(9):e49807. doi: 10.15252/embr.201949807 (PMC7507572; doi:10.15252/embr.201949807)
Supplement: Supplementary file 1 — Appendix [file EMBR-21-e49807-s001.pdf]

<u>Appendix:</u> Hypothalamic CDK4 regulates thermogenesis by modulating sympathetic innervation and activation of BAT

Table of contents

1. Appendix Figure S1 2
2. Appendix Figure S2 3
3. Appendix Figure S3 4
4. Appendix Figure S4 6
5. Appendix supplemental methods 7

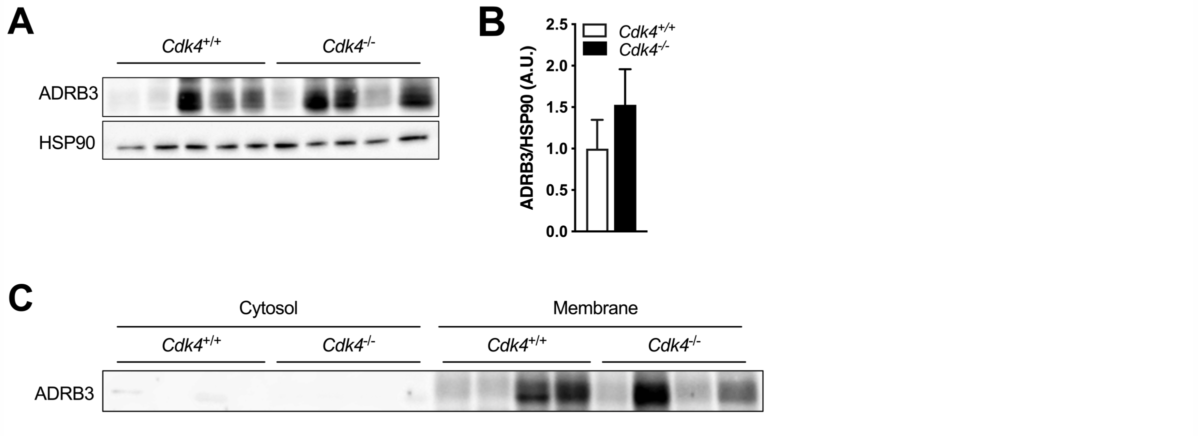

Appendix Figure S1 - Expression of beta-3 adrenergic receptor (ADRB3) in scWAT of *Cdk4*$^{-/-}$ mice.

A-B Western blot analysis (A) and quantification of ADRB3 protein expression (n=5 biological replicates) (B) in scWAT of *Cdk4*$^{+/+}$ and *Cdk4*$^{-/-}$ mice. HSP90 was used as loading control.

C Western blot analysis of the location of ADRB3 with tissue subcellular fractions (cytoplasm and plasma membrane) of scWAT of *Cdk4*$^{+/+}$ and *Cdk4*$^{-/-}$ mice (n=4 biological replicates).

Data information: All data are shown as the mean ± SEM.

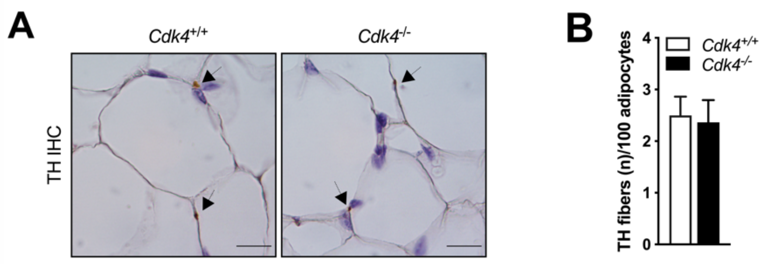

Appendix Figure S2 - TH immunohistochemical analysis in scWAT of *Cdk4*$^{-/-}$ mice.

A-B TH immunohistochemical staining in scWAT sections (scale bar 10 µm, arrows indicate TH parenchymal fibers) (A) and corresponding quantification of the number of TH fibers relative to 100 adipocytes (B) (*Cdk4*$^{+/+}$ (n=6) and *Cdk4*$^{-/-}$ (n=5)).

Data information: All data are shown as the mean ± SEM; Student's t-test was used for statistical analysis. **P<0.01.

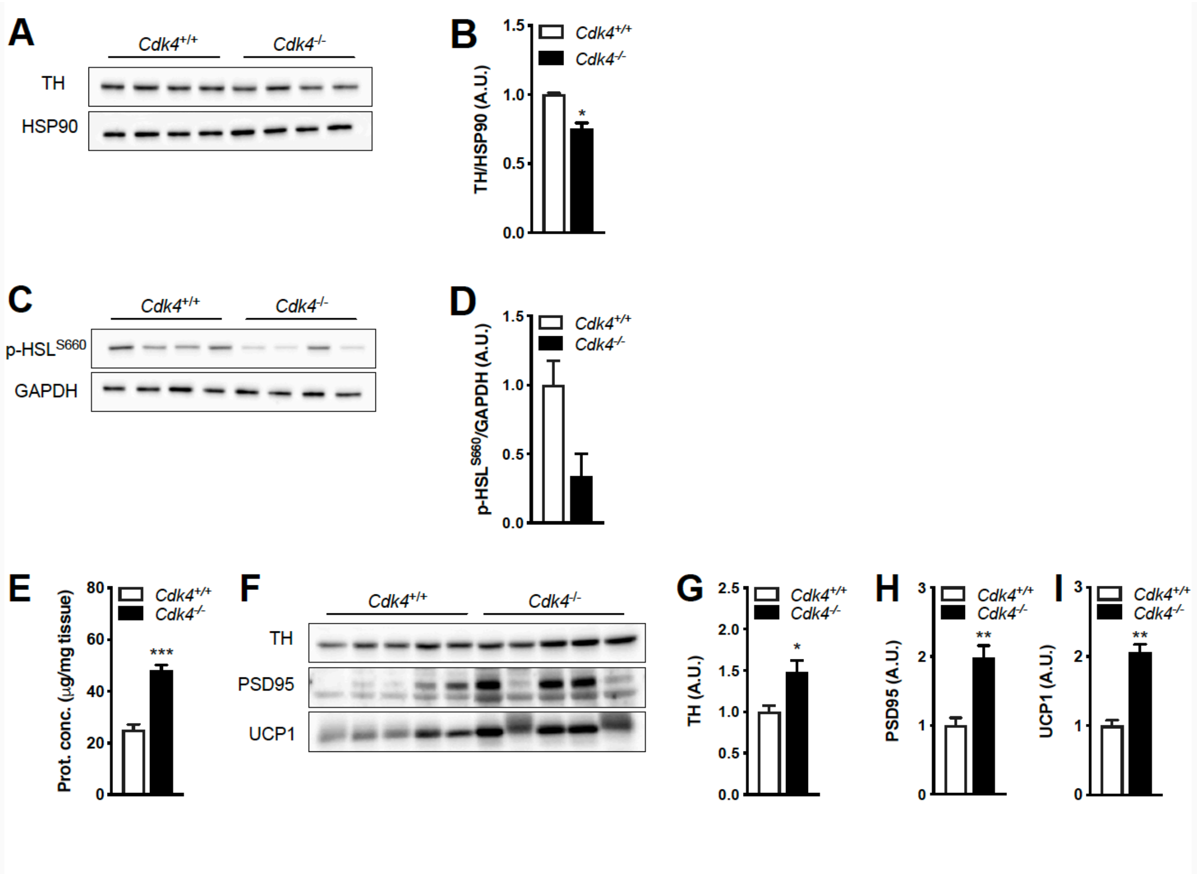

Appendix Figure S3 - Expression of TH and p-HSL S660 in iBAT of *Cdk4*$^{-/-}$ mice.

A-B Western blot analysis (A) and quantification of TH (B) in iBAT of *Cdk4*$^{+/+}$ and *Cdk4*$^{-/-}$ mice (n=4 biological replicates). HSP90 was used as loading control.

C-D Western blot analysis (C) and quantification of p-HSL S660 protein expression (D) (n=4 biological replicates) in iBAT of *Cdk4*$^{+/+}$ and *Cdk4*$^{-/-}$ mice. GAPDH was used as loading control.

E Protein concentration of iBAT protein extracts from equal amounts of tissues in *Cdk4*$^{+/+}$ (n=5) and *Cdk4*$^{-/-}$ (n=5) mice.

F-G Western blot analysis (F) and quantification of TH (G) and PSD95 (H) and UCP1 (I) in iBAT protein extracts of equal amount for *Cdk4*$^{+/+}$ and *Cdk4*$^{-/-}$ mice (n=5 biological replicates).

Data information: All data are shown as the mean ± SEM; Student's t-test (D) and Mann-Whitney U test (B, F, G) were used for statistical analyses. **P<0.01, ***P<0.001, ***P<0.005.

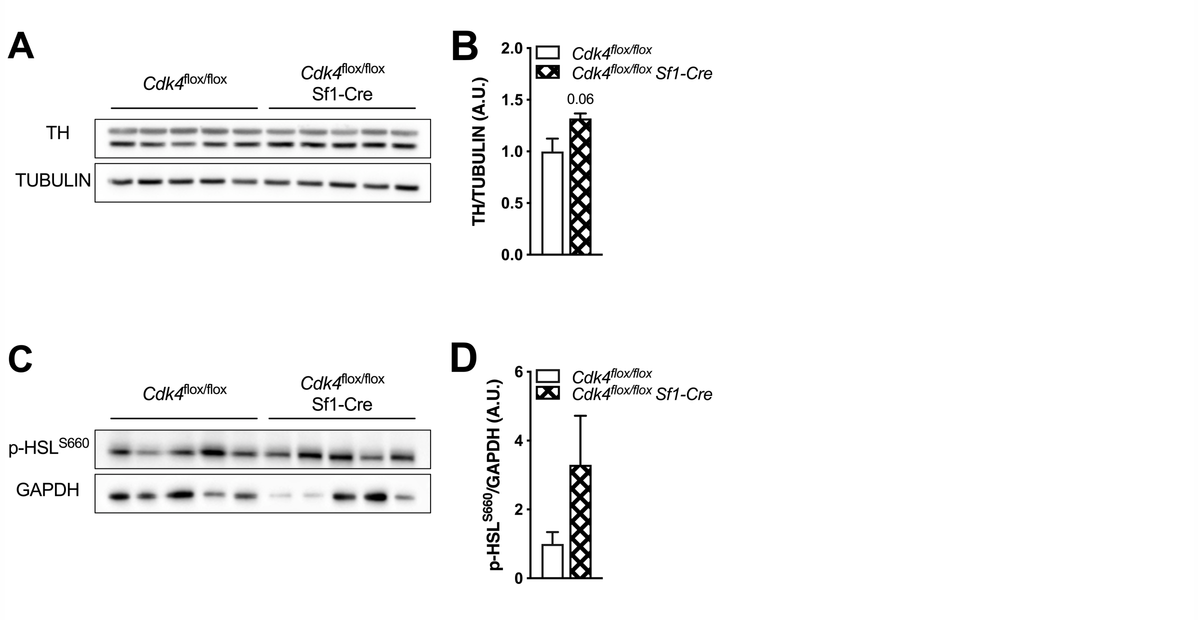

Appendix Figure S4 - Expression of TH and p-HSL S660 in BAT of *Cdk4$^{flox/flox}$* Sf1-Cre mice.

A-B Western blot analysis (A) and quantification of TH (B) (n=5 biological replicates) in iBAT of *Cdk4$^{flox/flox}$ and Cdk4$^{flox/flox}$ Sf1-Cre mice*. Tubulin was used as loading control.

C. Western blot analysis (C) and quantification (D) of p-HSL S660 protein expression (n=5 biological replicates) in iBAT of *Cdk4$^{flox/flox}$ and Cdk4$^{flox/flox}$ Sf1-Cre mice*. GAPDH was used as loading control.

Data information: All data are shown as the mean ± SEM; Mann-Whitney U test was used for statistical analysis.

Appendix supplemental methods

Subcellular fractionation of scWAT. Sample preparation was performed as previously described (Xia, Pessentheiner et al., 2018). 100mg of frozen tissue (scWAT from *Cdk4*$^{+/+}$ and *Cdk4*$^{-/-}$ mice were minced in 1 mL hypotonic lysis buffer containing 50 mM HEPES, 50 mM sucrose, 1 mM EDTA, 100 mM NaCl and 1 x PIC, and were thoroughly dounced (60 strokes) with a tissue Grinder (VWR VOS14) at 1600 rpm. Lysates were centrifuged at 5000 g, 4 °C for 10 minutes to pellet organelles and cell debris. The supernatant was centrifuged at 100,000 g, 4 °C for 1 hour. The resulting membrane pellets were resuspended in RIPA buffer with 1 x PIC for Western blotting. The supernatant represented the cytosolic fraction. Cytosolic proteins were precipitated with four times the sample volume of cold (-20°C) acetone. After overnight precipitation, samples were centrifuge at 13,000× g for 10 minutes. The resulting pellet was resuspended in RIPA buffer with 1x PIC. 20μg of protein were loaded for Western blotting.

Protein extraction and western blot analysis. Proteins from BAT and scWAT were extracted using mammalian protein extraction reagent (MPER, Pierce) as per the manufacturer's instructions, separated by gel electrophoresis and analyzed using the antibodies anti-ADRB3 (ab94506, Abcam. Kindly provided by Dr. Hadrien Demagny and Prof. Kristina Schoonjans), anti-PSD95 (ab18258, Abcam) and anti-p-HSL^{S660} (4126, Cell signaling).

References

Xia W, Pessentheiner AR, Hofer DC, Amor M, Schreiber R, Schoiswohl G, Eichmann TO, Walenta E, Itariu B, Prager G, Hackl H, Stulnig T, Kratky D, Rulicke T, Bogner-Strauss JG (2018) Loss of ABHD15 Impairs the Anti-lipolytic Action of Insulin by Altering PDE3B Stability and Contributes to Insulin Resistance. *Cell Rep* 23: 1948-1961
