# Supplementary material for: Hypothalamic CDK4 regulates thermogenesis by modulating sympathetic innervation of adipose tissues
Source: EMBO Rep. 2020 Jul 12;21(9):e49807. doi: 10.15252/embr.201949807 (PMC7507572; doi:10.15252/embr.201949807)
Supplement: Supplementary file 2 — Expanded View Figures PDF [file EMBR-21-e49807-s002.pdf]

Expanded View Figures

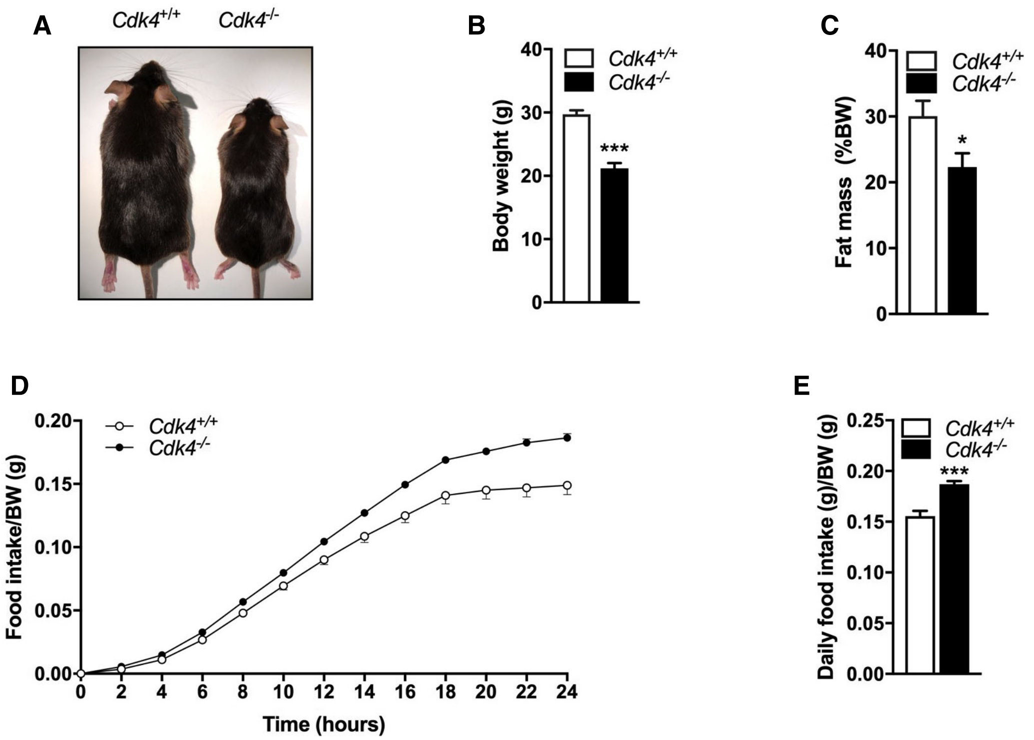

Figure EV1. *Cdk4*$^{-/-}$ mice are smaller and have increased food intake compared to their wild-type littermates (*Cdk4*$^{+/+}$).

A Whole-body image of *Cdk4*$^{+/+}$ and *Cdk4*$^{-/-}$ mice.

B, C Body weight (B) and fat mass percentage (C) of *Cdk4*$^{+/+}$ (n = 5) and *Cdk4*$^{-/-}$ (n = 6) mice.

D, E Food intake was measured using the Phenomaster device (TSE system) and is shown as cumulative intake over 24 h (D) and daily cumulative food intake (E) of 14-week-old *Cdk4*$^{+/+}$ (n = 7) and *Cdk4*$^{-/-}$ (n = 8) mice.

Data information: All data are shown as the mean ± SEM. Student's t-test was used for statistical analysis *$P < 0.05$; ***$P < 0.001$.
Source data are available online for this figure.

© 2020 The Authors

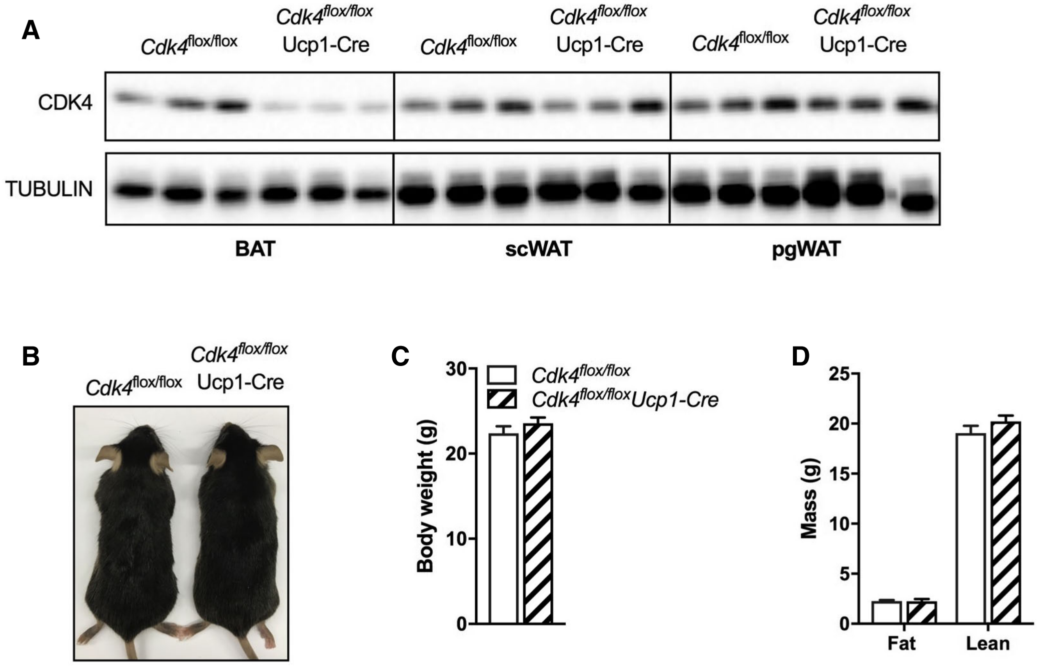

Figure EV2. *Cdk4* BAT-specific knockout mice have normal body weight, fat mass, and lean mass.

A Western blot for CDK4 expression in iBAT, scWAT, and pgWAT from *Cdk4*$^{flox/flox}$ (n = 3 biological replicates) and *Cdk4*$^{flox/flox}$ Ucp1-Cre (n = 3) mice. Tubulin was used as loading control. Please notice that upon amplification, the western blot images are abnormally pixelated. This is because the saturation of the images is shown in FigEV2A_Exposure_3.0sec.scn, which is a direct acquisition file from the Imager, and is found in the data source folder. A JPEG image is also available in this folder.

B Whole-body image of *Cdk4*$^{flox/flox}$ and *Cdk4*$^{flox/flox}$ Ucp1-Cre mice.

C, D Body weight (C) and body composition (fat and lean mass) (D) of *Cdk4*$^{flox/flox}$ (n = 8) and *Cdk4*$^{flox/flox}$ Ucp1-Cre (n = 8) mice.

Data information: All data are shown as the mean ± SEM.
Source data are available online for this figure.

 © 2020 The Authors

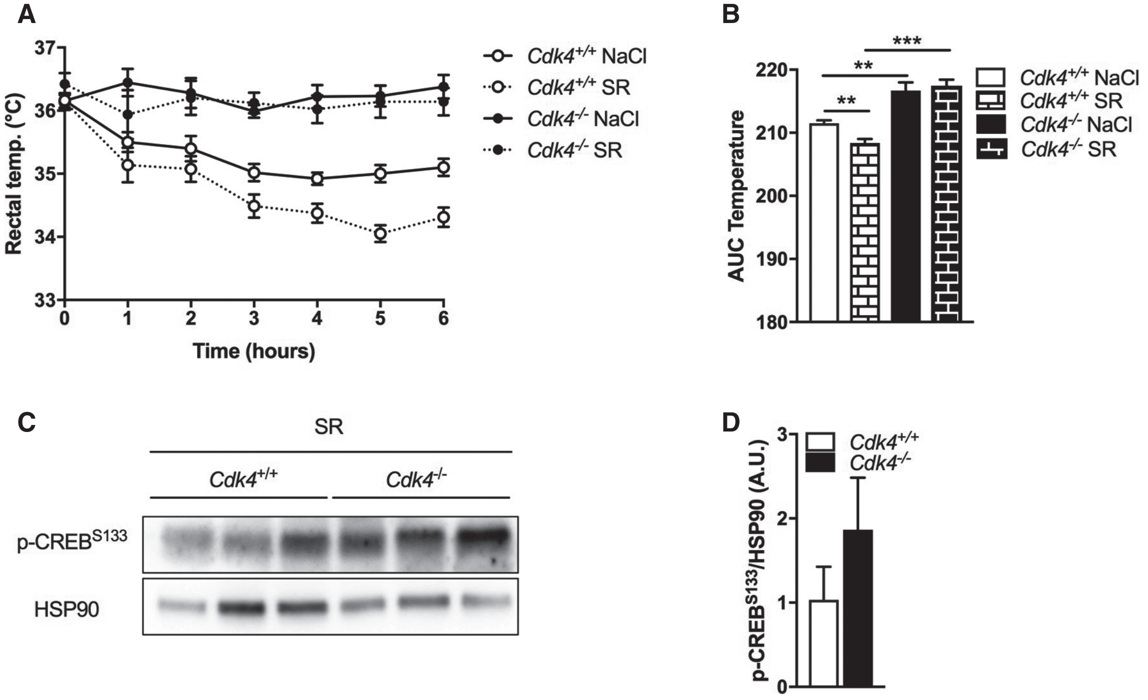

Figure EV3. β3-receptor inhibition does not blunt the increased thermogenic response of *Cdk4*$^{-/-}$ animals.

A, B Acute cold test (4°C) after 5 days of treatment with a β3-adrenergic antagonist (SR; *Cdk4*$^{+/+}$ [n = 5] and *Cdk4*$^{-/-}$ [n = 5]) or vehicle (NaCl; *Cdk4*$^{+/+}$ [n = 8] and *Cdk4*$^{-/-}$ [n = 9]) (A) and corresponding quantification of the area under the curve (AUC) (B).

C, D Western blot analysis (C) and quantification of p-CREB S133 protein expression after 7 days of SR treatment (D) (n = 3 biological replicates). HSP90 was used as loading control.

Data information: All data are shown as the mean ± SEM. Student's t-test was used for statistical analyses. **$P < 0.01$, ***$P < 0.001$.
Source data are available online for this figure.

Figure EV4. *Cdk4* deficiency in Sf1 neurons does not affect body weight, body composition, or indirect calorimetry measures. ▶

A, B Body weight (A) and body composition (fat mass and lean mass) (B) of *Cdk4*$^{flox/flox}$ (n = 17) and *Cdk4*$^{flox/flox}$ Sf1-Cre (n = 11) mice.

C–H Indirect calorimetry was performed using the Oxymax apparatus (Columbus Instruments) in *Cdk4*$^{flox/flox}$ (n = 6) and *Cdk4*$^{flox/flox}$ Sf1-Cre (n = 5) mice. Whole-body oxygen consumption rate (VO_2) (C, D), respiratory exchange ratio (RER) (E, F), and energy expenditure (G, H) were measured at 24°C during the light phase (white rectangle) and dark phase (black rectangle).

I, J CalR was used to implement GLM-regression plot for each group corresponding to the association between energy balance and total body mass at 24°C (I). The CalR interface displays each mouse as a dot, and the standard error of mean for each group in gray. The "mass effect" and "group effect" were analyzed using a generalized linear model (GLM) using body weight as a covariate. The results of this analysis are shown in table (J) for *Cdk4*$^{flox/flox}$ (n = 6) and *Cdk4*$^{flox/flox}$ Sf1-Cre (n = 5) mice.

Data information: All data are shown as the mean ± SEM.

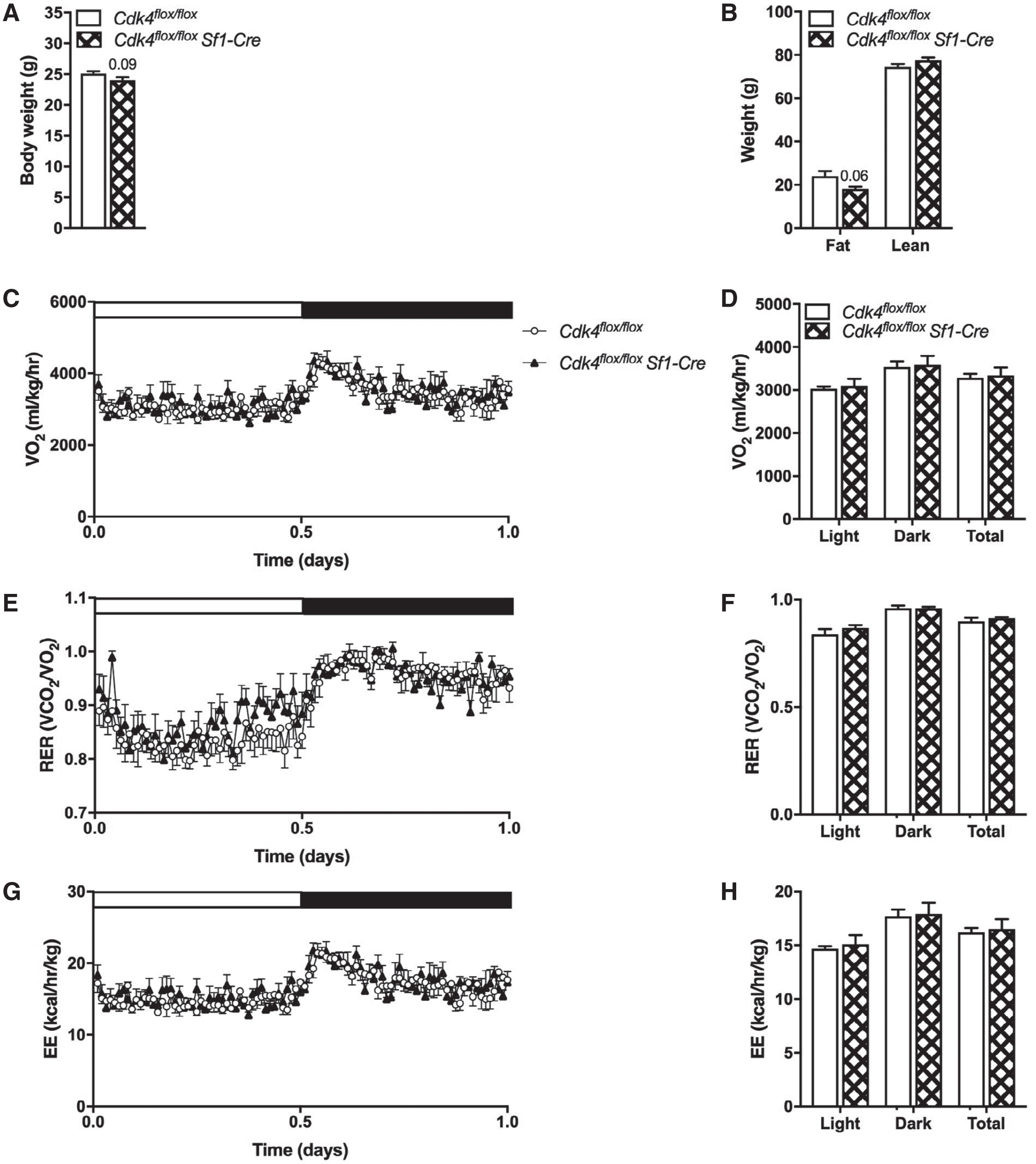

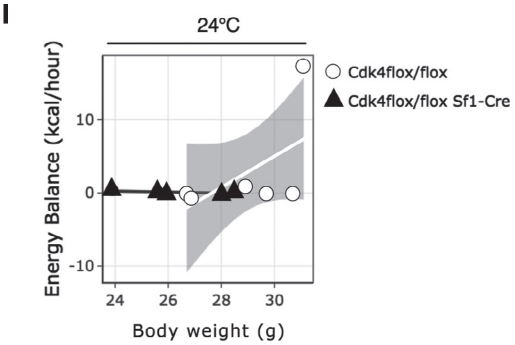

J

	24°C		
Effect	Mass	Group	GLM
Oxygen Consumption (ml/hr)	0.8159	0.2338	
Carbon Dioxide Production (ml/hr)	0.6008	0.4934	
Energy Expenditure (kcal/hour)	0.7562	0.2788	
Energy Balance (kcal/hour)	0.2474	0.7553	
	24°C		ANOVA
Effect		Group	
Respiratory Exchange Ratio		0.4689	

Figure EV4.

 © 2020 The Authors

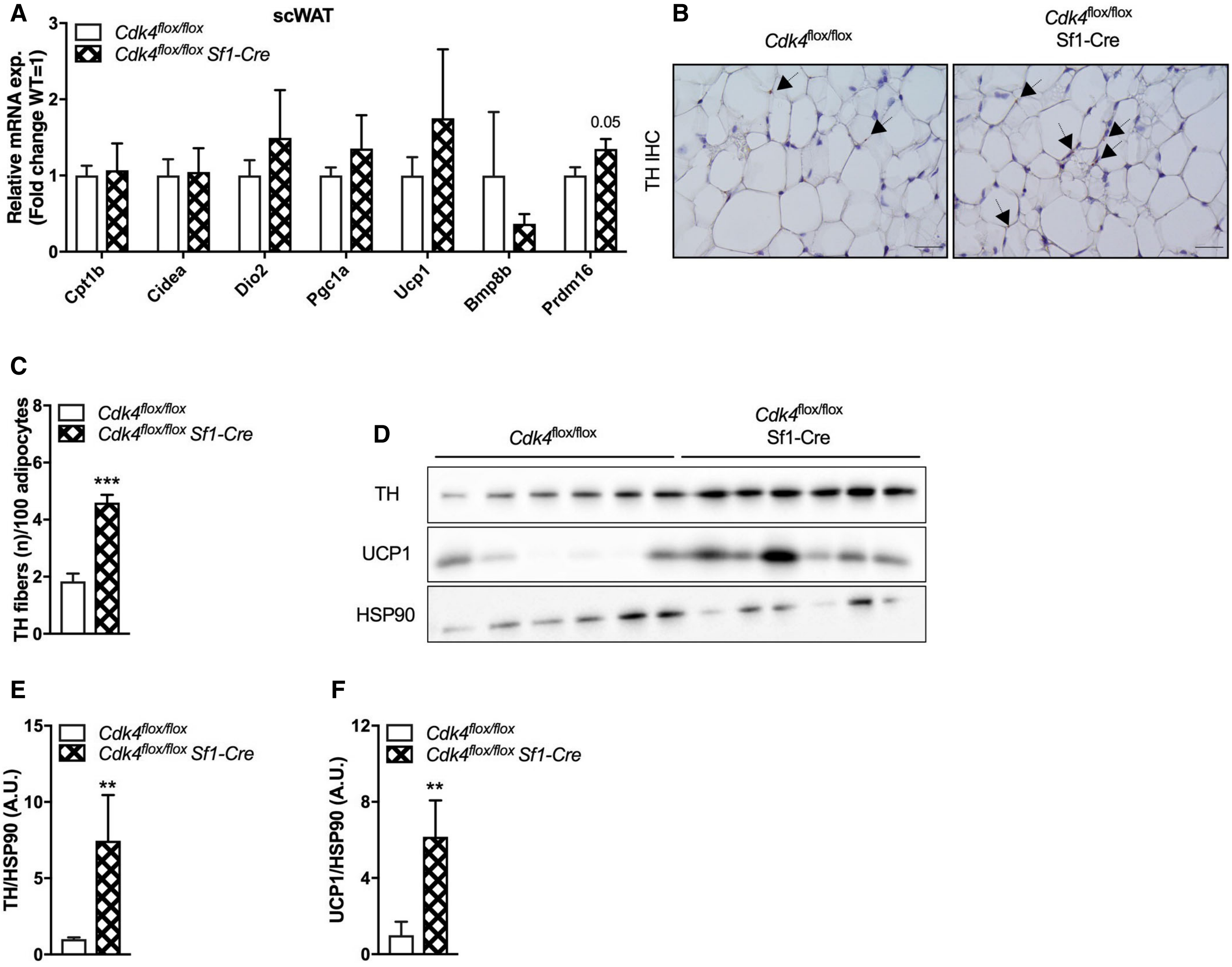

Figure EV5. Increased sympathetic innervation and WAT browning in *Cdk4*$^{flox/flox}$ Sf1-Cre mice.

A Expression of thermogenic genes in scWAT of *Cdk4*$^{flox/flox}$ (n = 9) and *Cdk4*$^{flox/flox}$ Sf1-Cre (n = 8) mice as assessed by RT–PCR.

B, C TH immunohistochemical (IHC) staining in iBAT sections (scale bar 20 μm, arrows indicate TH parenchymal fibers) (B) and corresponding quantification of the number of TH fibers relative to 100 adipocytes (C) (*Cdk4*$^{flox/flox}$ [n = 5] and *Cdk4*$^{flox/flox}$ Sf1-Cre [n = 6]).

D–F Western blot analysis (D) and quantification of TH (E) and UCP1 protein expression (n = 6 biological replicates) (F). HSP90 was used as loading control.

Data information: All data are shown as the mean ± SEM. Student's t-test (C) and Mann–Whitney U-test (E, F) were used for statistical analyses. $^{**}P < 0.01$, $^{***}P < 0.001$.

Source data are available online for this figure.
