# Supplementary figures and images for: Hypothalamic CDK4 regulates thermogenesis by modulating sympathetic innervation of adipose tissues
Source: EMBO Rep. 2020 Jul 12;21(9):e49807. doi: 10.15252/embr.201949807 (PMC7507572; doi:10.15252/embr.201949807)

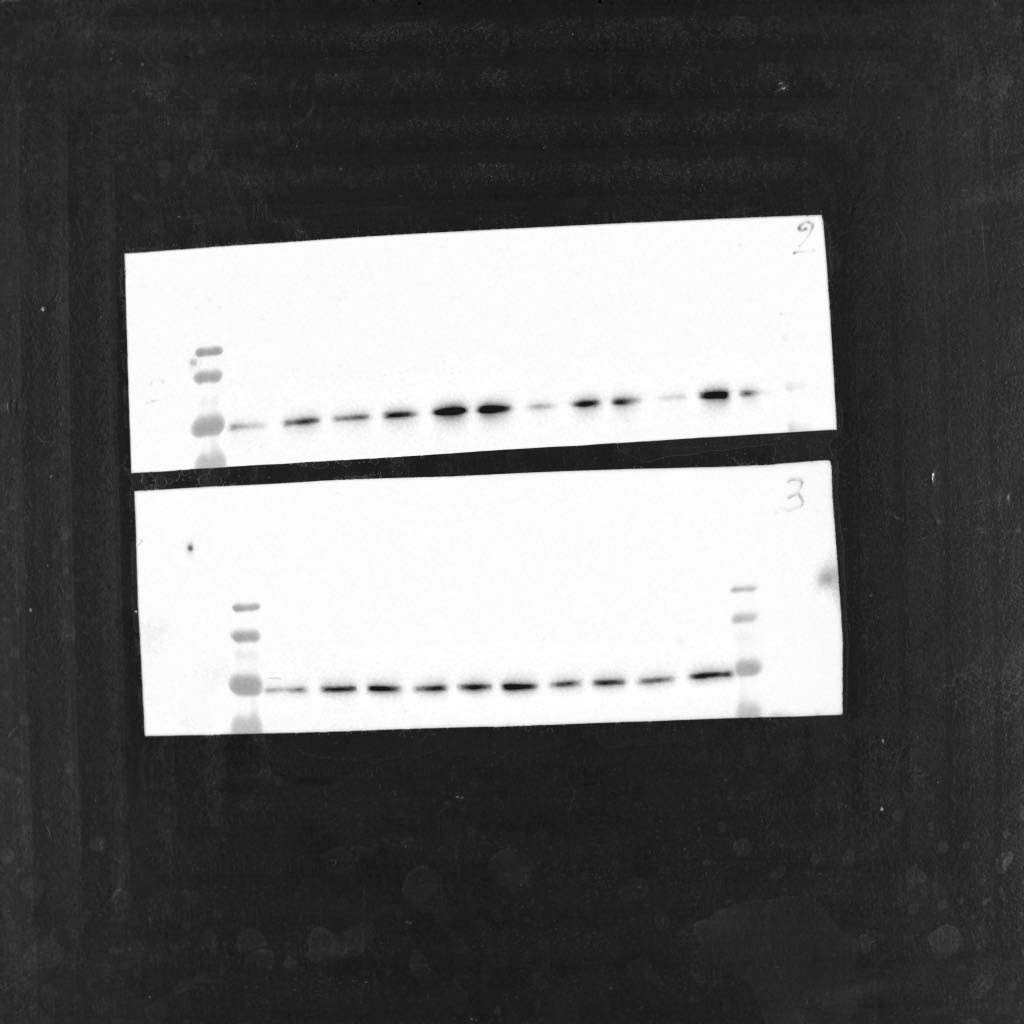

Supplement: Supplementary file 3 — Source Data for Expanded View and Appendix [file EMBR-21-e49807-s009.zip › source_data_EV_figures/Source_Data_FigEV5/EMBOR-2019-49807V1_FigEV5D_WB_HSP90.jpg]

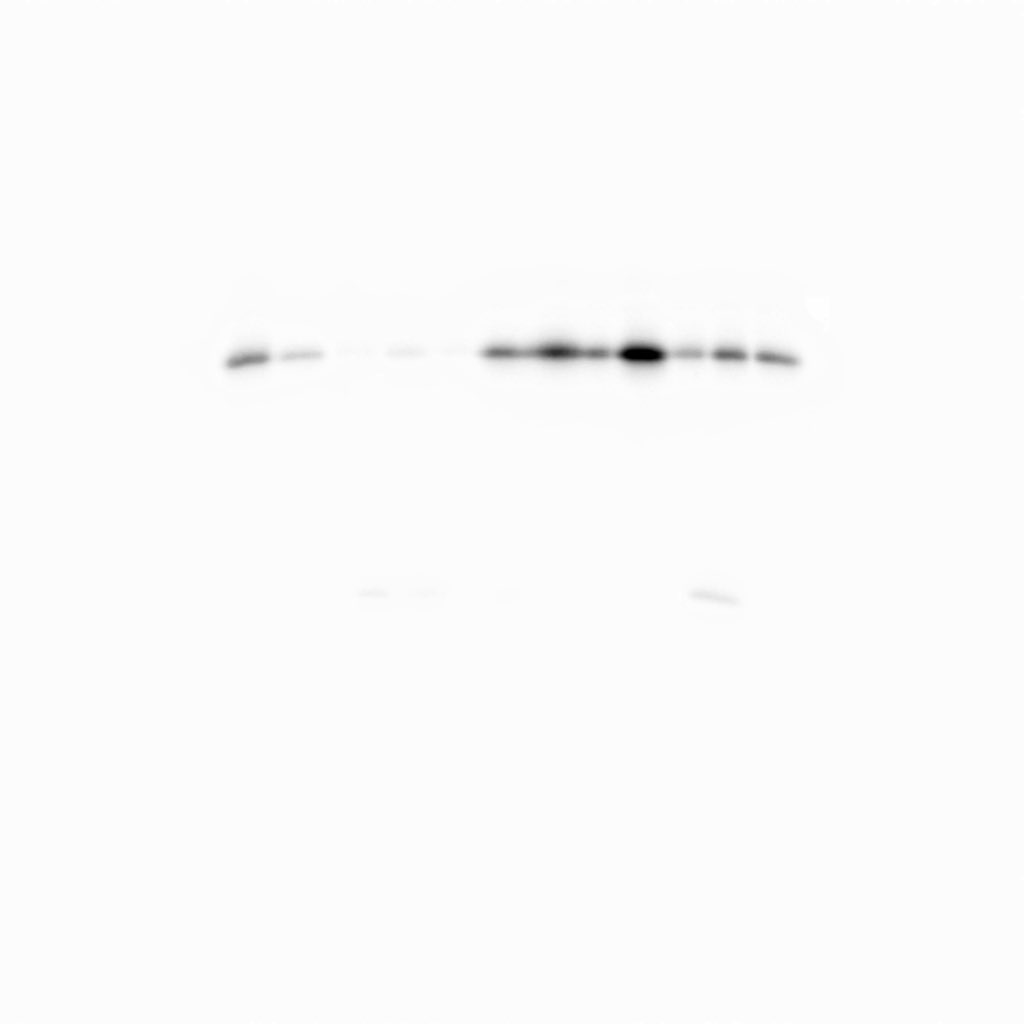

Supplement: Supplementary file 3 — Source Data for Expanded View and Appendix [file EMBR-21-e49807-s009.zip › source_data_EV_figures/Source_Data_FigEV5/EMBOR-2019-49807V1_FigEV5D_WB_UCP1.jpg]

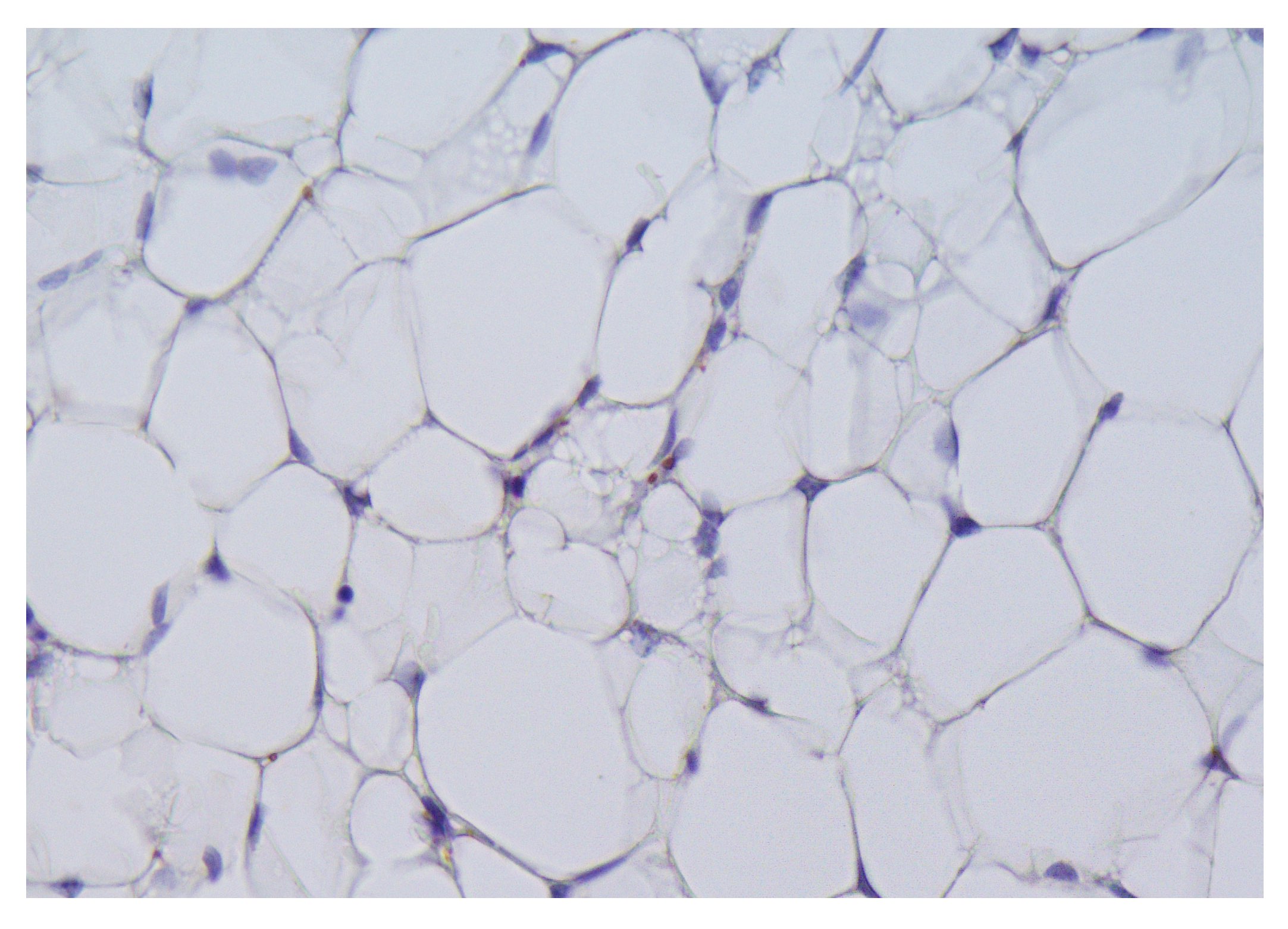

Supplement: Supplementary file 3 — Source Data for Expanded View and Appendix [file EMBR-21-e49807-s009.zip › source_data_EV_figures/Source_Data_FigEV5/EMBOR-2019-49807V1_FigEV5B_TH_KO.jpg]

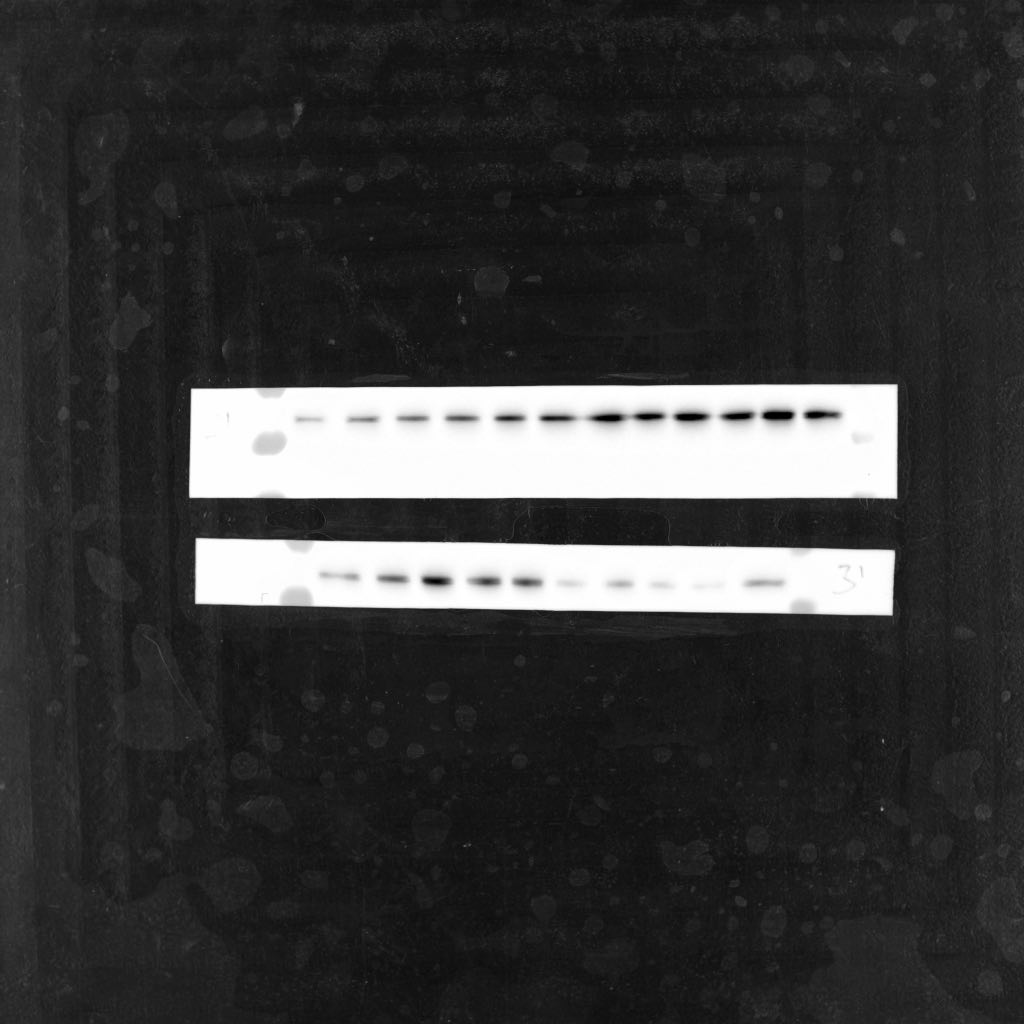

Supplement: Supplementary file 3 — Source Data for Expanded View and Appendix [file EMBR-21-e49807-s009.zip › source_data_EV_figures/Source_Data_FigEV5/EMBOR-2019-49807V1_FigEV5D_WB_TH.jpg]

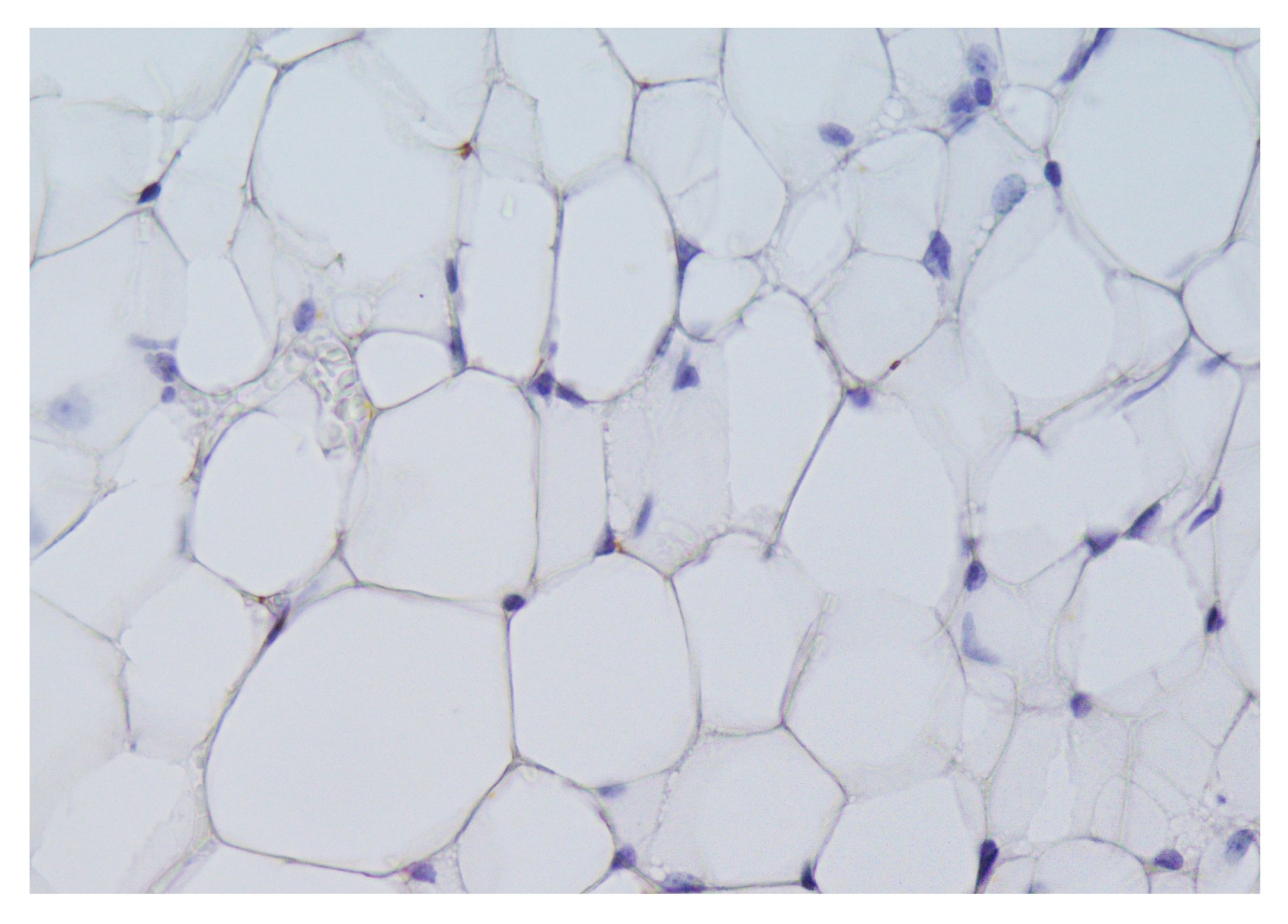

Supplement: Supplementary file 3 — Source Data for Expanded View and Appendix [file EMBR-21-e49807-s009.zip › source_data_EV_figures/Source_Data_FigEV5/EMBOR-2019-49807V1_FigEV5B_TH_WT.jpg]

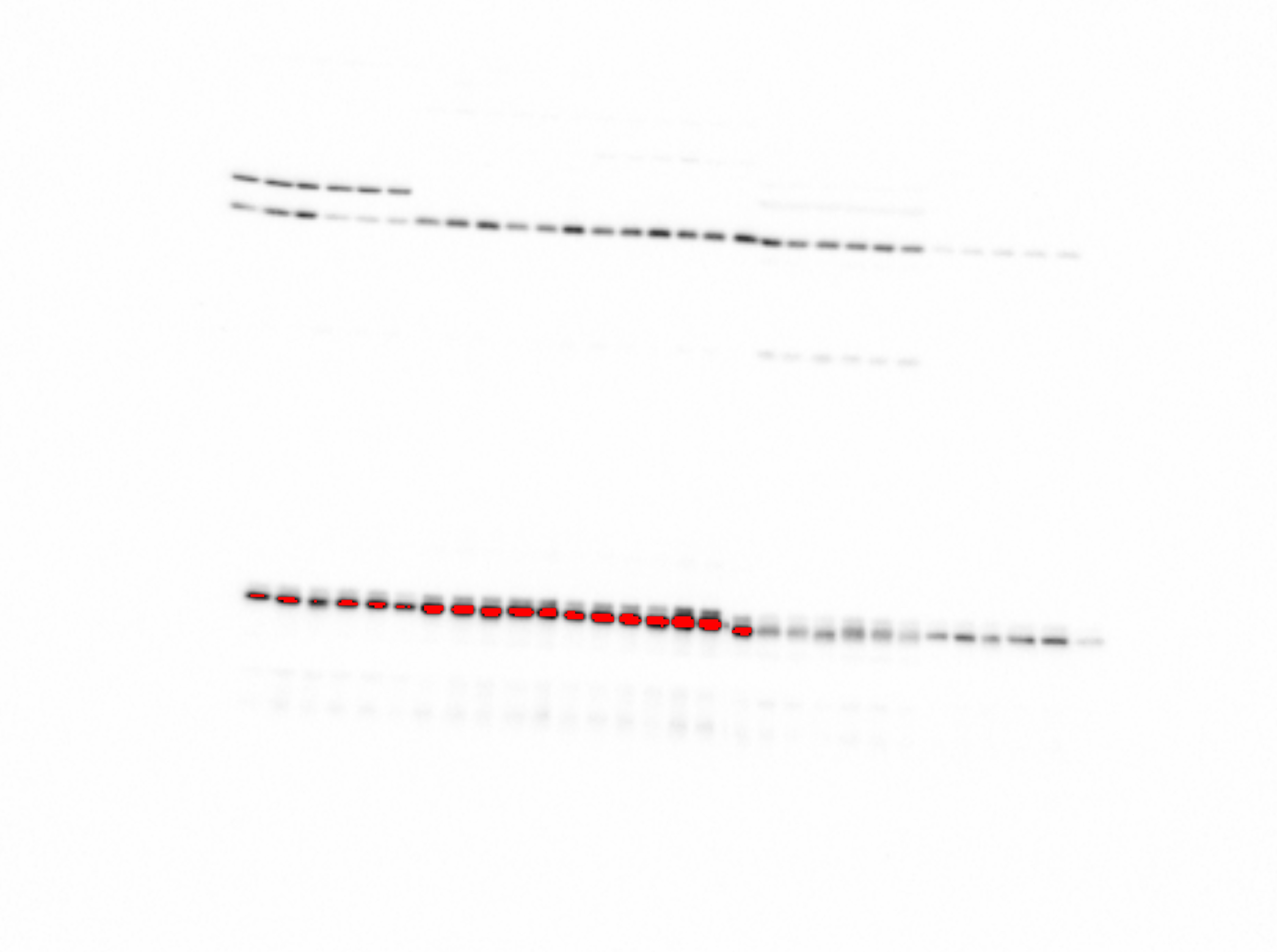

Supplement: Supplementary file 3 — Source Data for Expanded View and Appendix [file EMBR-21-e49807-s009.zip › source_data_EV_figures/Source_Data_FigEV2/EV2_Exposure_3.0sec.jpg]

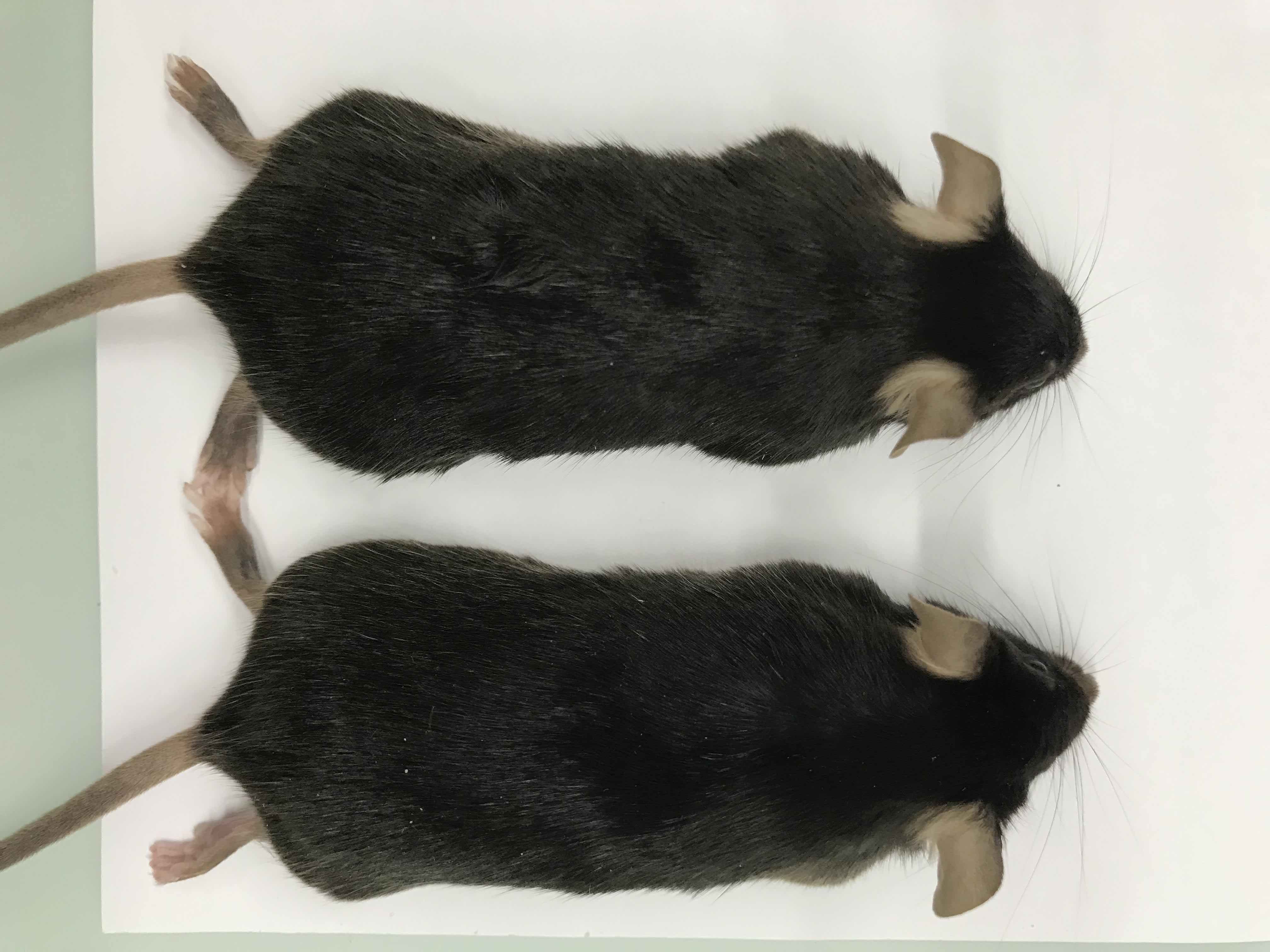

Supplement: Supplementary file 3 — Source Data for Expanded View and Appendix [file EMBR-21-e49807-s009.zip › source_data_EV_figures/Source_Data_FigEV2/EMBOR-2019-49807V1_FigEV2B_WHOLE BODY.jpg]

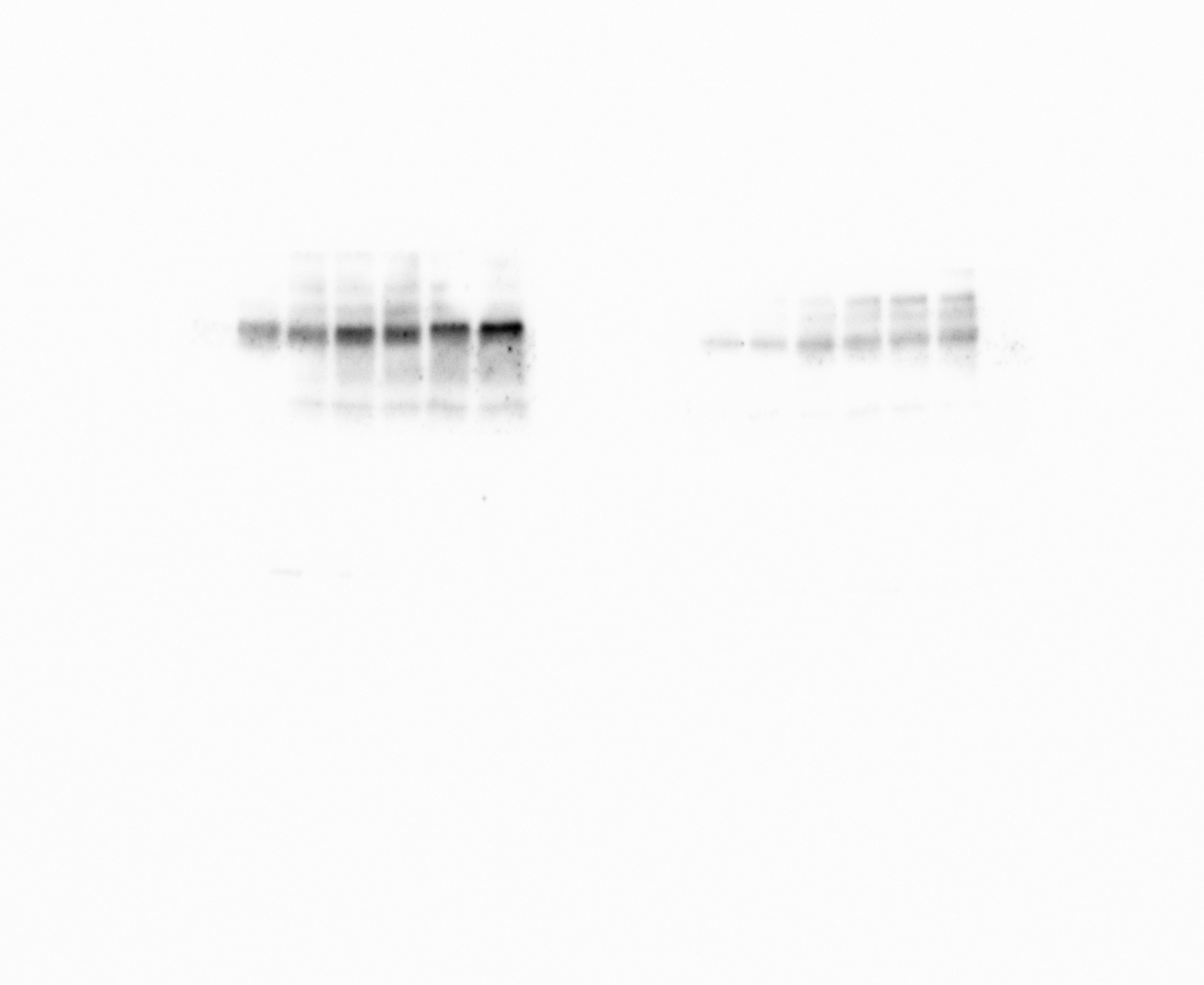

Supplement: Supplementary file 3 — Source Data for Expanded View and Appendix [file EMBR-21-e49807-s009.zip › source_data_EV_figures/Source_Data_FigEV3/EMBOR-2019-49807V1_FigEV3C_WB_PCREBS133.jpg]

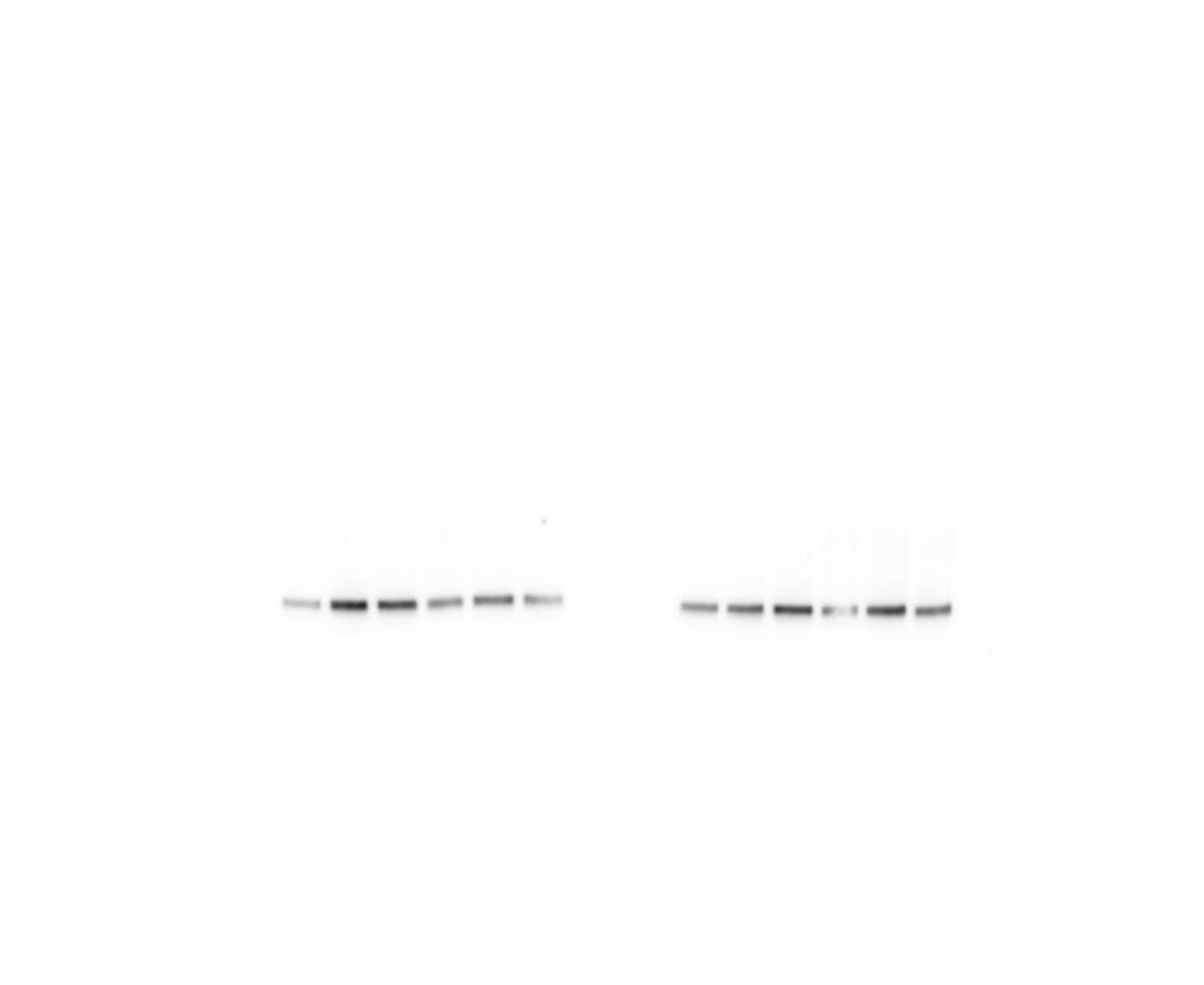

Supplement: Supplementary file 3 — Source Data for Expanded View and Appendix [file EMBR-21-e49807-s009.zip › source_data_EV_figures/Source_Data_FigEV3/EMBOR-2019-49807V1_FigEV3C_WB_HSP90.jpg]

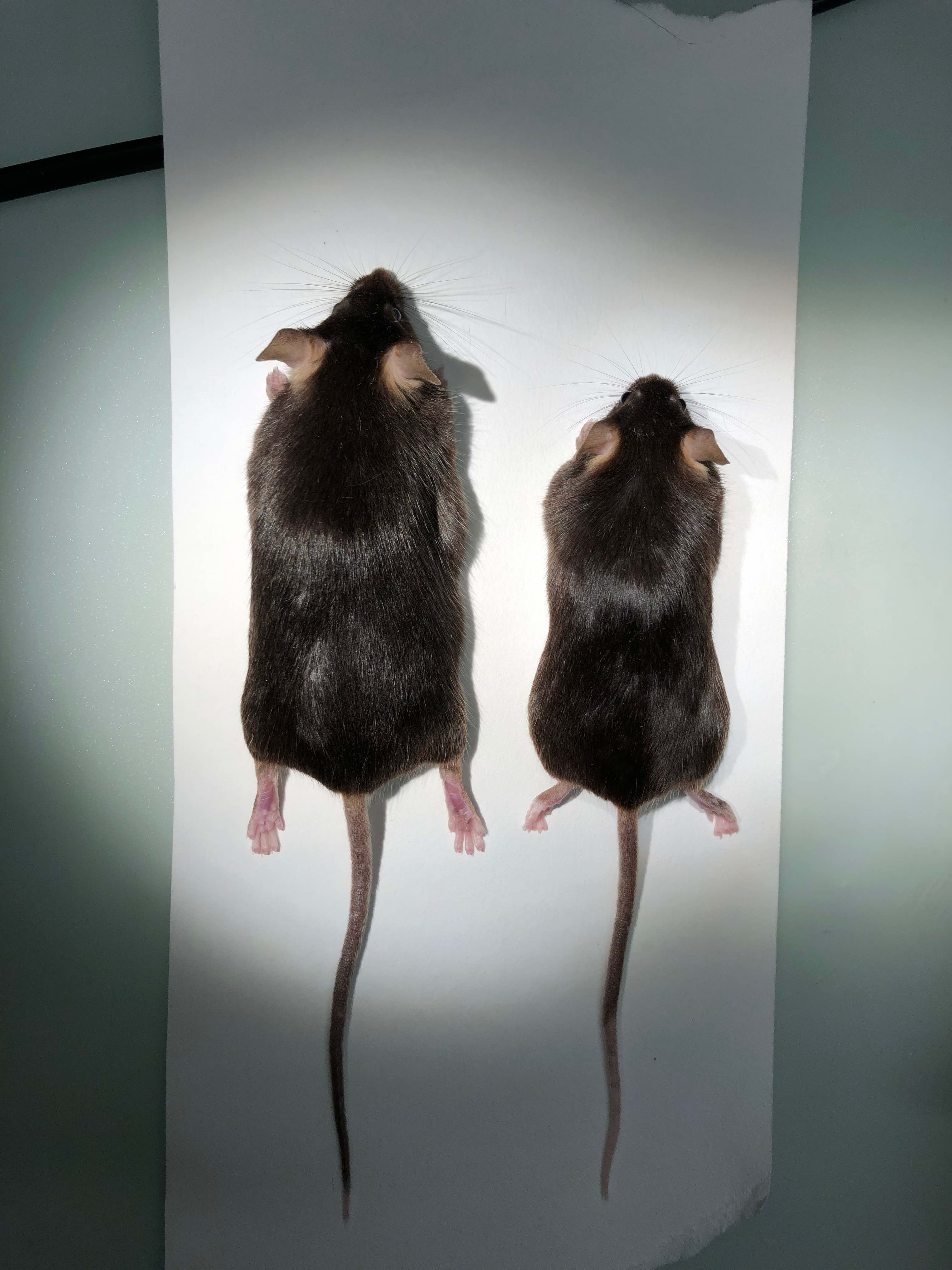

Supplement: Supplementary file 3 — Source Data for Expanded View and Appendix [file EMBR-21-e49807-s009.zip › source_data_EV_figures/Source_Data_FigEV1/EMBOR-2019-49807V1_FigEV1A_WHOLE BODY.jpg]

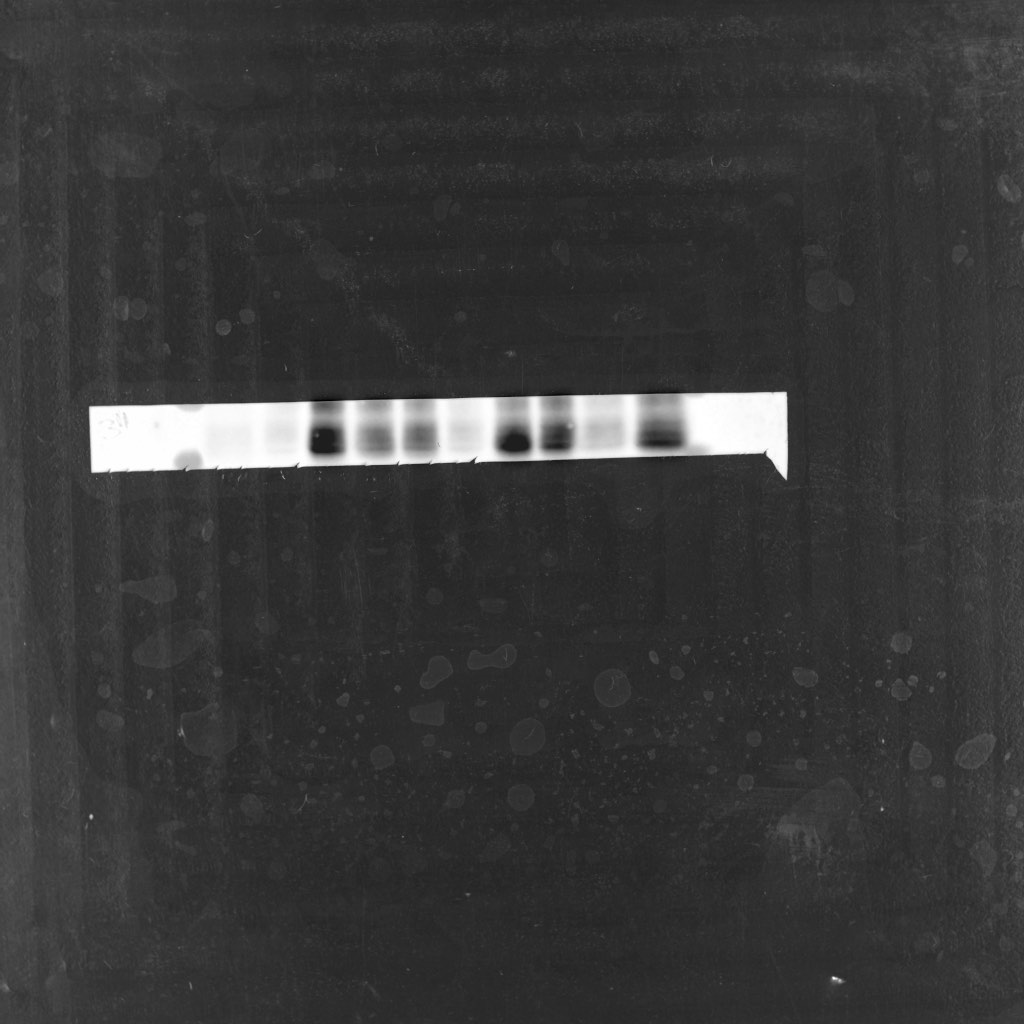

Supplement: Supplementary file 3 — Source Data for Expanded View and Appendix [file EMBR-21-e49807-s009.zip › Source_Data_appendix_figures/Source_Data_Appendix_FigS1/EMBOR-2019-49807V1_FigS1A_WB_ADRB3.jpg]

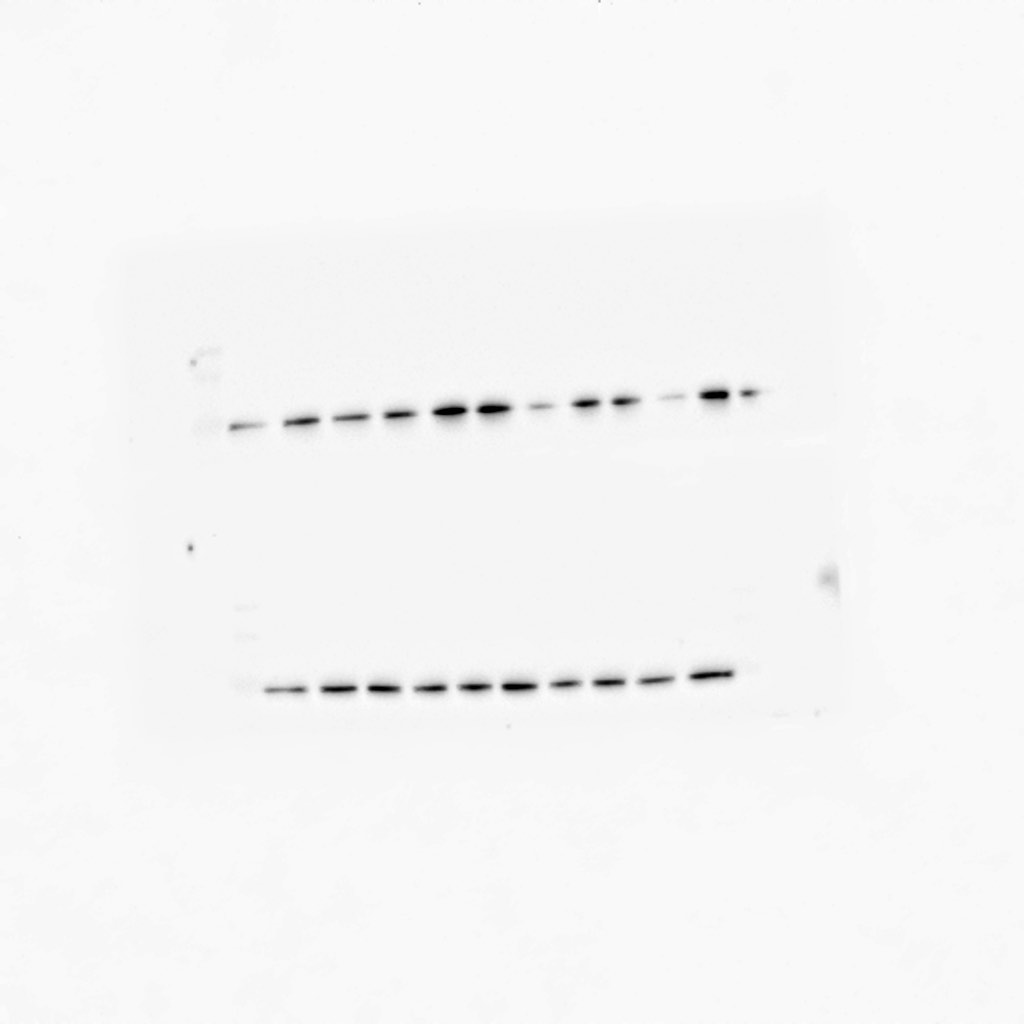

Supplement: Supplementary file 3 — Source Data for Expanded View and Appendix [file EMBR-21-e49807-s009.zip › Source_Data_appendix_figures/Source_Data_Appendix_FigS1/EMBOR-2019-49807V1_FigS1A_WB_HSP90.jpg]

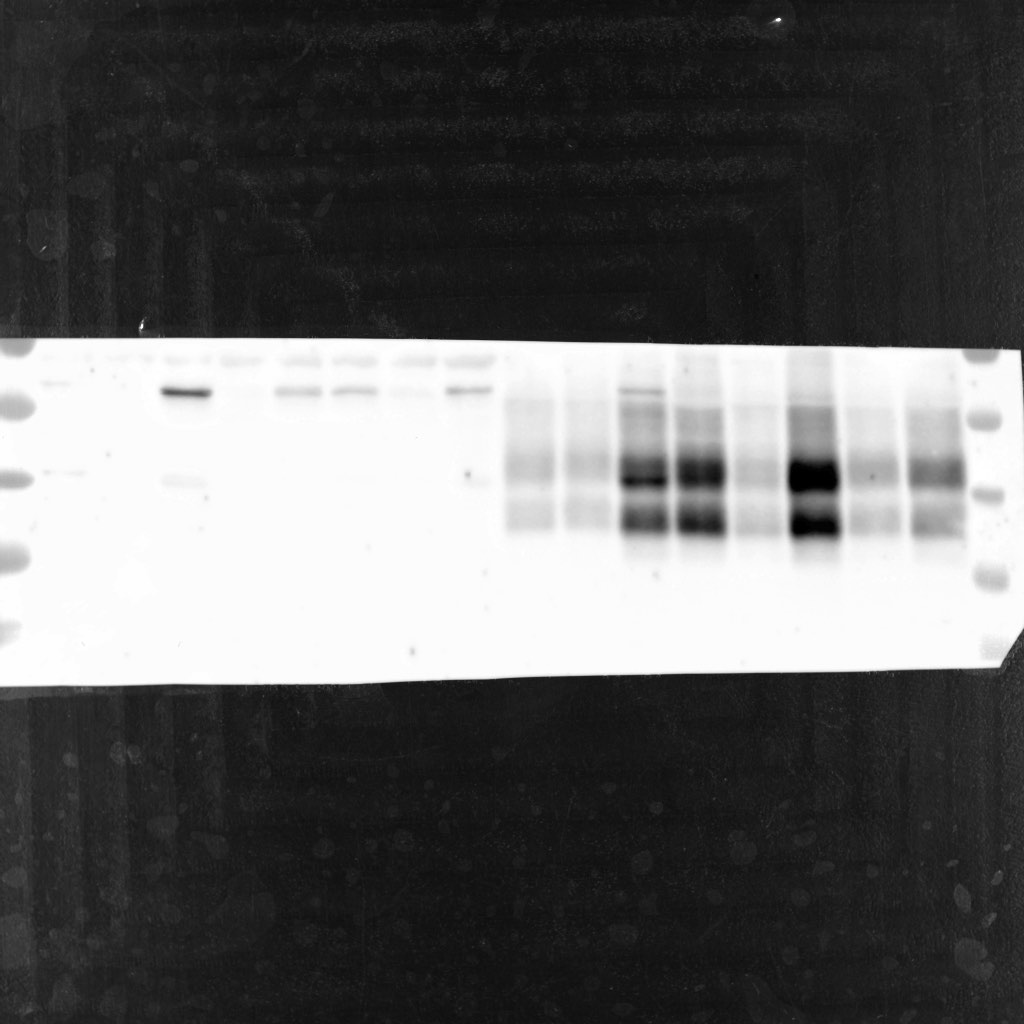

Supplement: Supplementary file 3 — Source Data for Expanded View and Appendix [file EMBR-21-e49807-s009.zip › Source_Data_appendix_figures/Source_Data_Appendix_FigS1/EMBOR-2019-49807V1_FigS1C_WB_ADRB3.jpg]

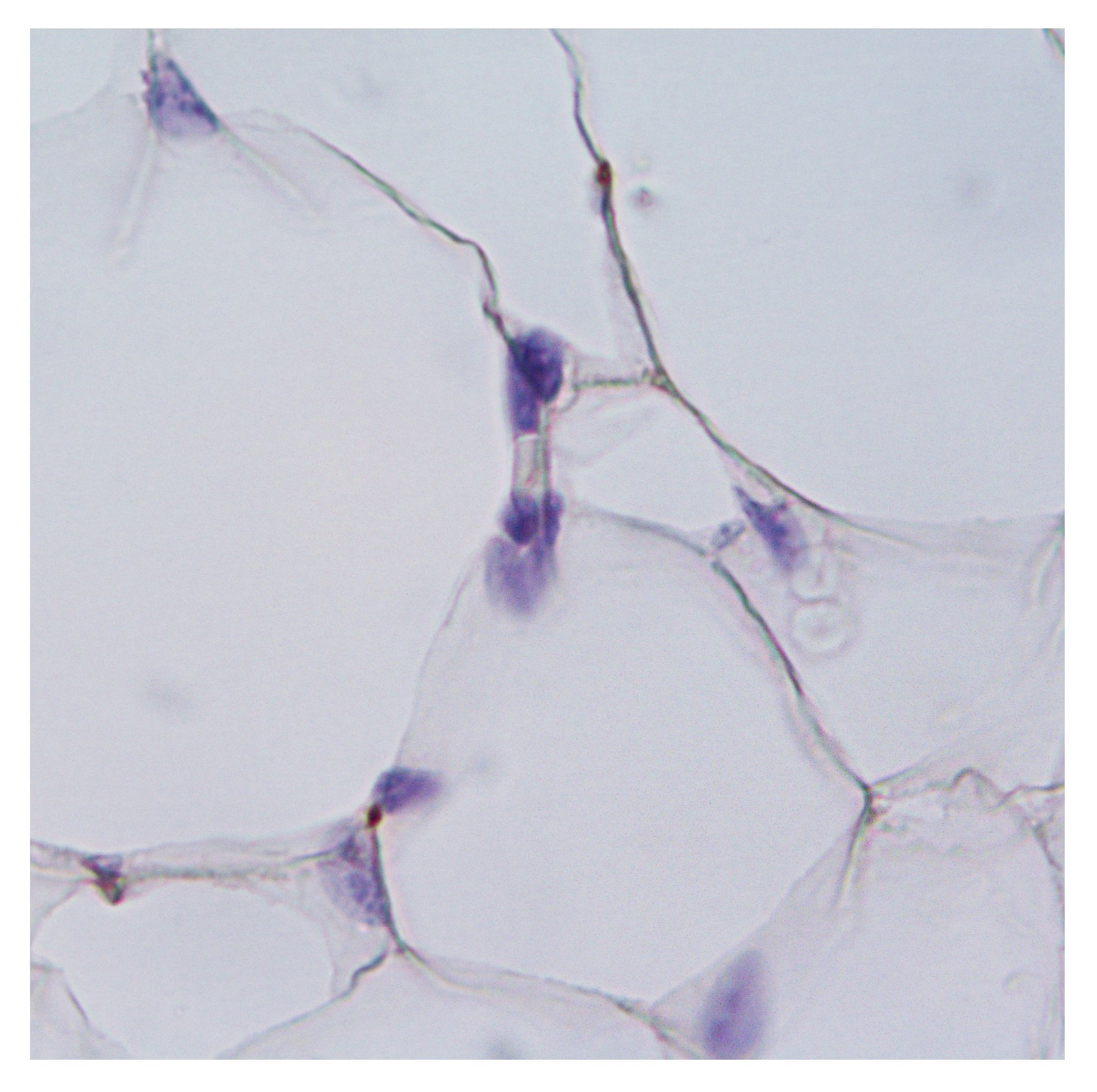

Supplement: Supplementary file 3 — Source Data for Expanded View and Appendix [file EMBR-21-e49807-s009.zip › Source_Data_appendix_figures/Source_Data_Appendix_FigS2/EMBOR-2019-49807V1_FigS2A_TH_KO.jpg]

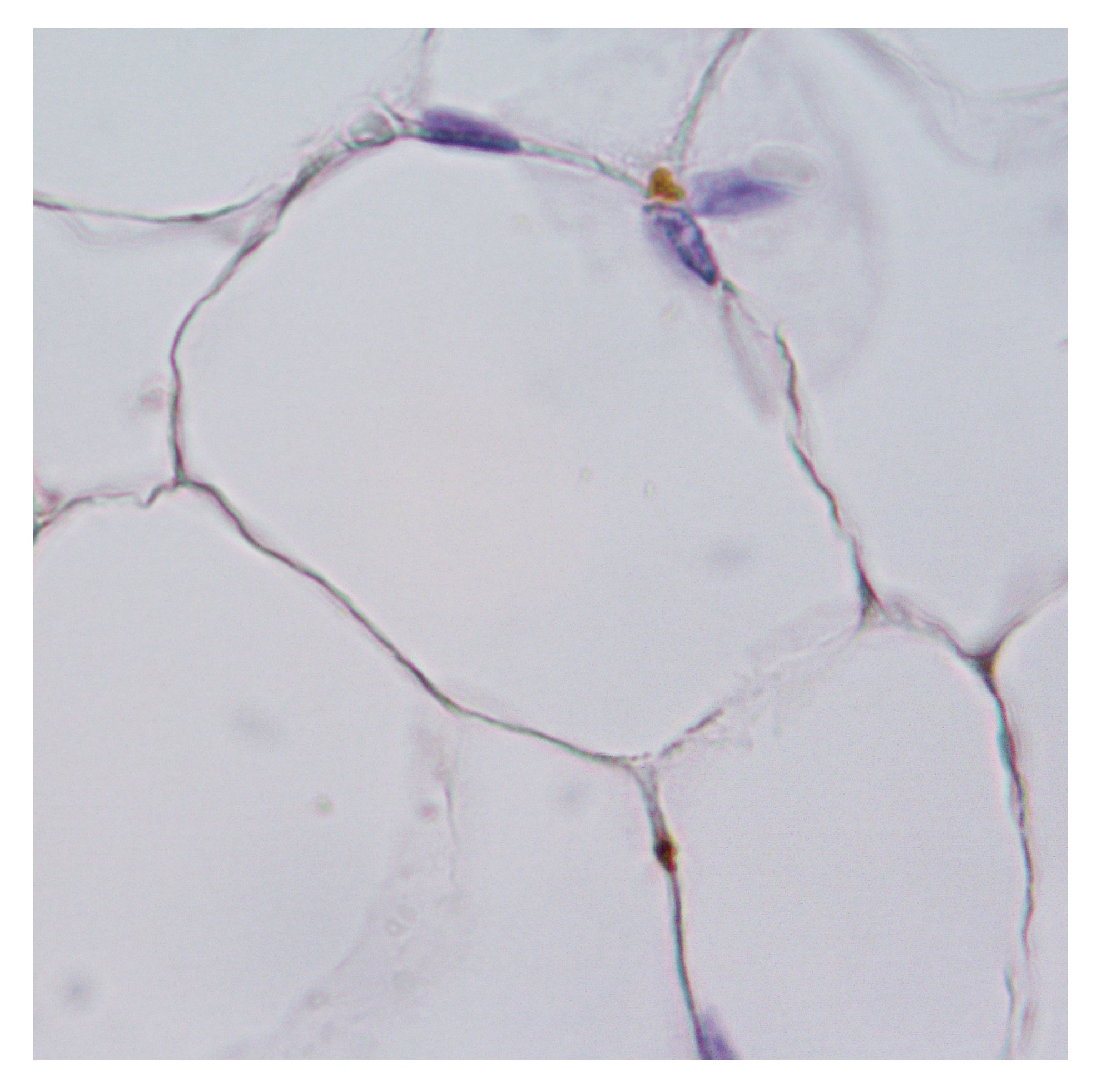

Supplement: Supplementary file 3 — Source Data for Expanded View and Appendix [file EMBR-21-e49807-s009.zip › Source_Data_appendix_figures/Source_Data_Appendix_FigS2/EMBOR-2019-49807V1_FigS2A_TH_WT.jpg]

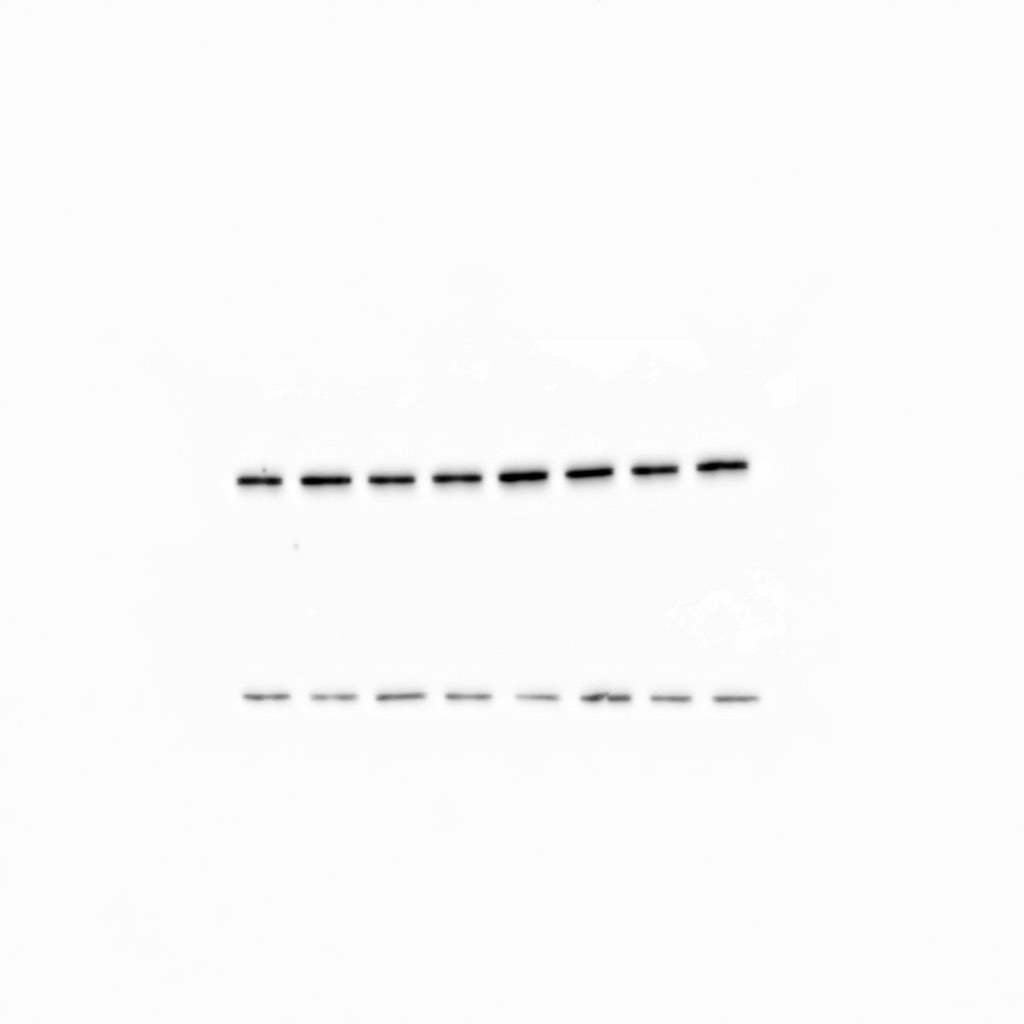

Supplement: Supplementary file 3 — Source Data for Expanded View and Appendix [file EMBR-21-e49807-s009.zip › Source_Data_appendix_figures/Source_Data_Appendix_FigS3/EMBOR-2019-49807V1_FigS3A_WB_HSP90.jpg]

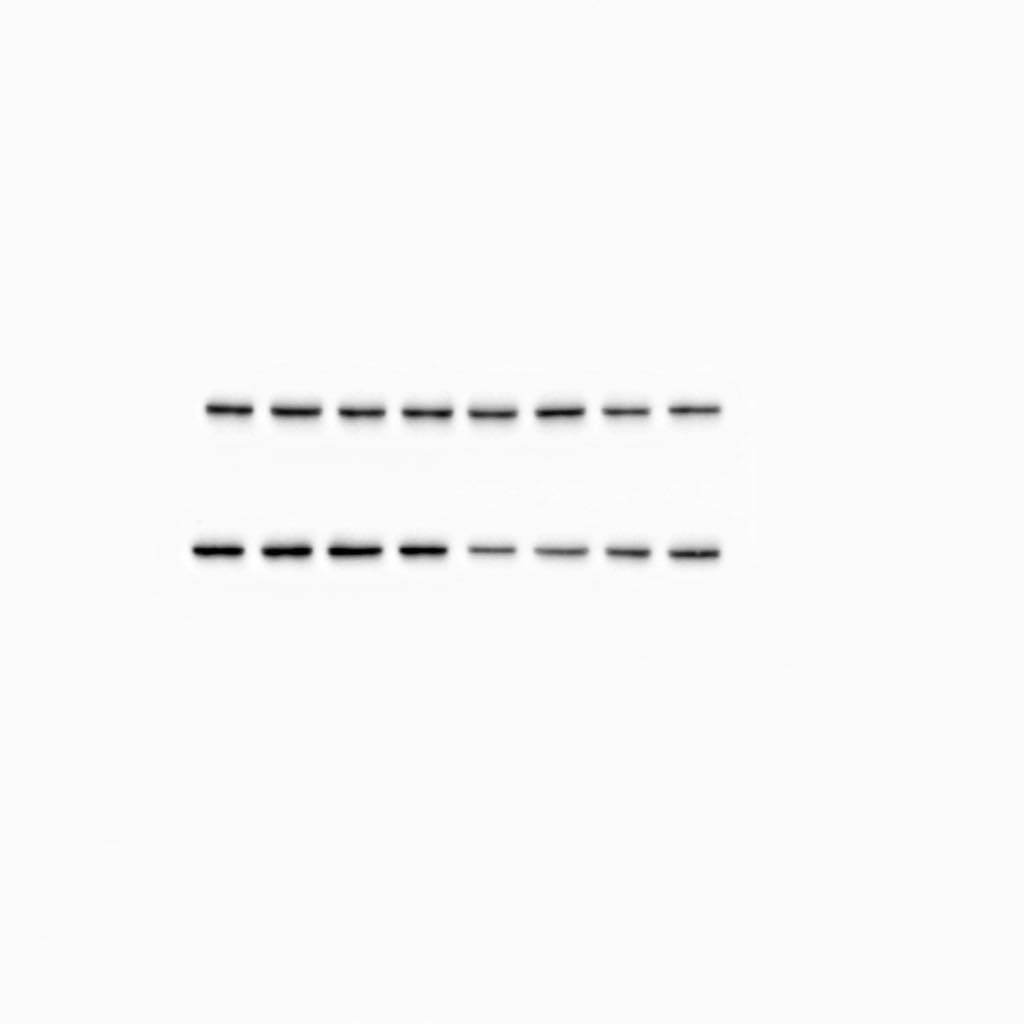

Supplement: Supplementary file 3 — Source Data for Expanded View and Appendix [file EMBR-21-e49807-s009.zip › Source_Data_appendix_figures/Source_Data_Appendix_FigS3/EMBOR-2019-49807V1_FigS3A_WB_TH.jpg]

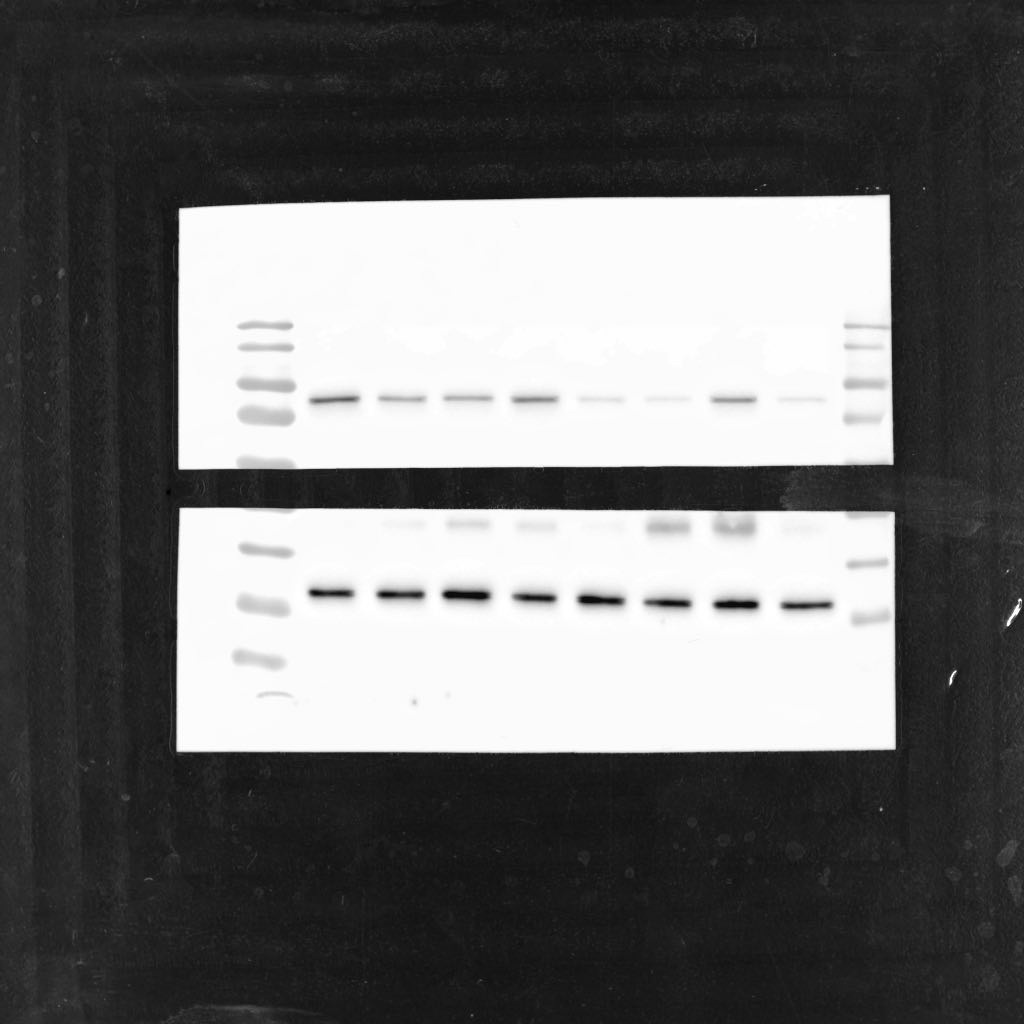

Supplement: Supplementary file 3 — Source Data for Expanded View and Appendix [file EMBR-21-e49807-s009.zip › Source_Data_appendix_figures/Source_Data_Appendix_FigS3/EMBOR-2019-49807V1_FigS3C_WB_GAPDH.jpg]

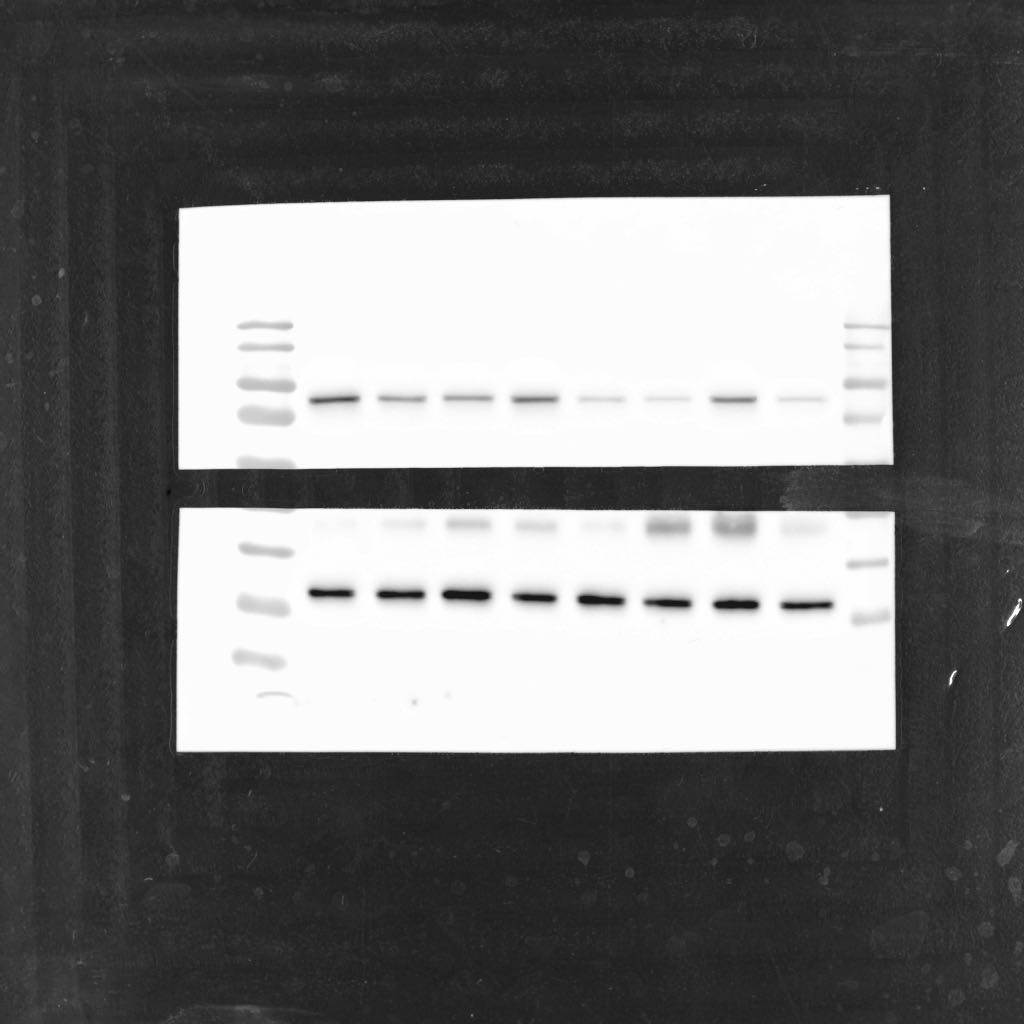

Supplement: Supplementary file 3 — Source Data for Expanded View and Appendix [file EMBR-21-e49807-s009.zip › Source_Data_appendix_figures/Source_Data_Appendix_FigS3/EMBOR-2019-49807V1_FigS3C_WB_PHSLS660.jpg]

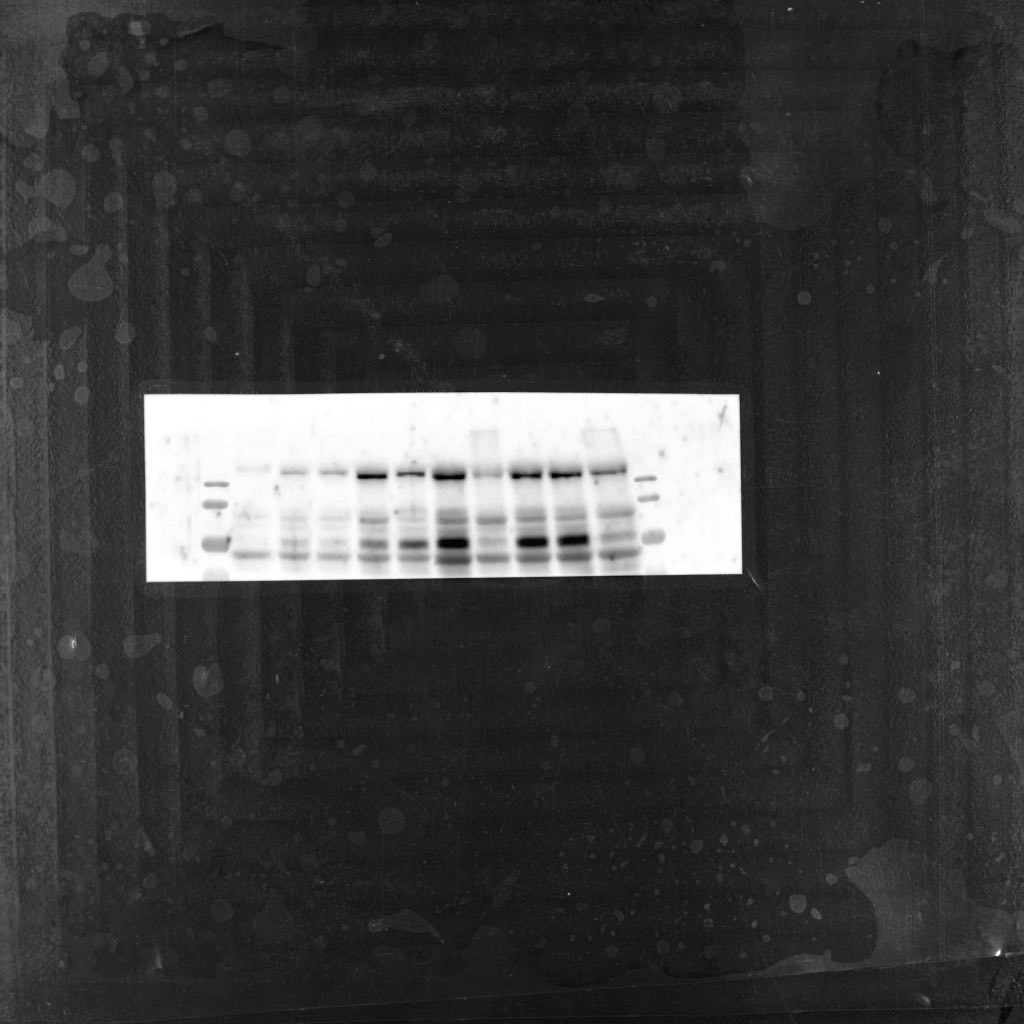

Supplement: Supplementary file 3 — Source Data for Expanded View and Appendix [file EMBR-21-e49807-s009.zip › Source_Data_appendix_figures/Source_Data_Appendix_FigS3/EMBOR-2019-49807V1_FigS3F_WB_PSD95.jpg]

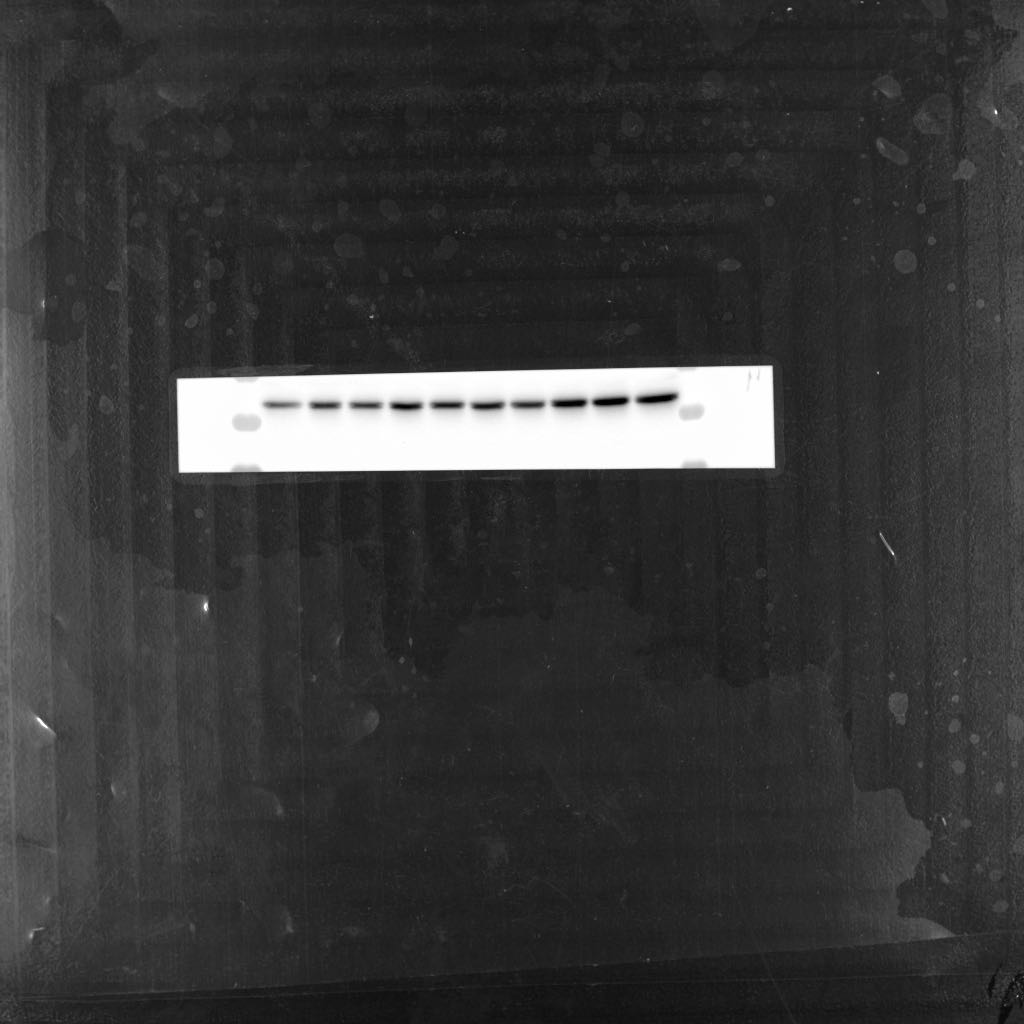

Supplement: Supplementary file 3 — Source Data for Expanded View and Appendix [file EMBR-21-e49807-s009.zip › Source_Data_appendix_figures/Source_Data_Appendix_FigS3/EMBOR-2019-49807V1_FigS3F_WB_TH.jpg]

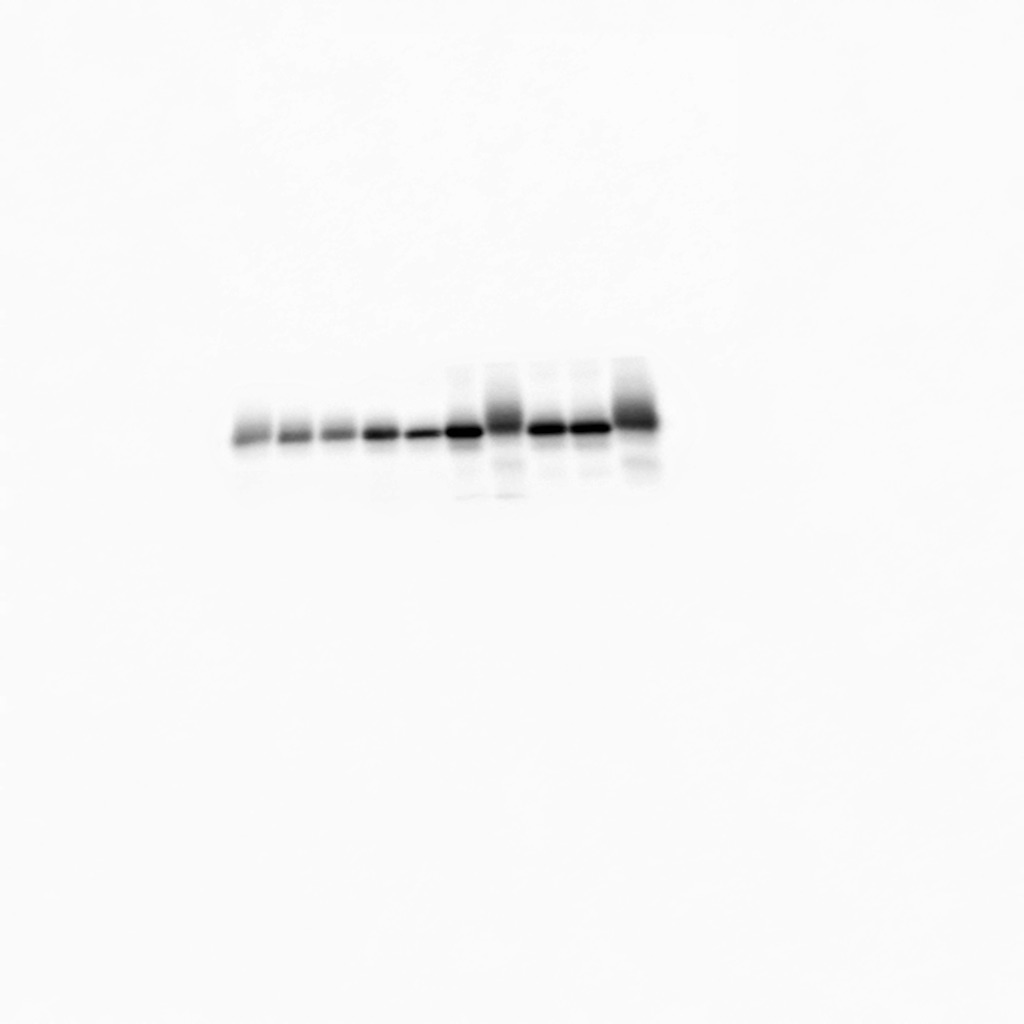

Supplement: Supplementary file 3 — Source Data for Expanded View and Appendix [file EMBR-21-e49807-s009.zip › Source_Data_appendix_figures/Source_Data_Appendix_FigS3/EMBOR-2019-49807V1_FigS3F_WB_UCP1.jpg]

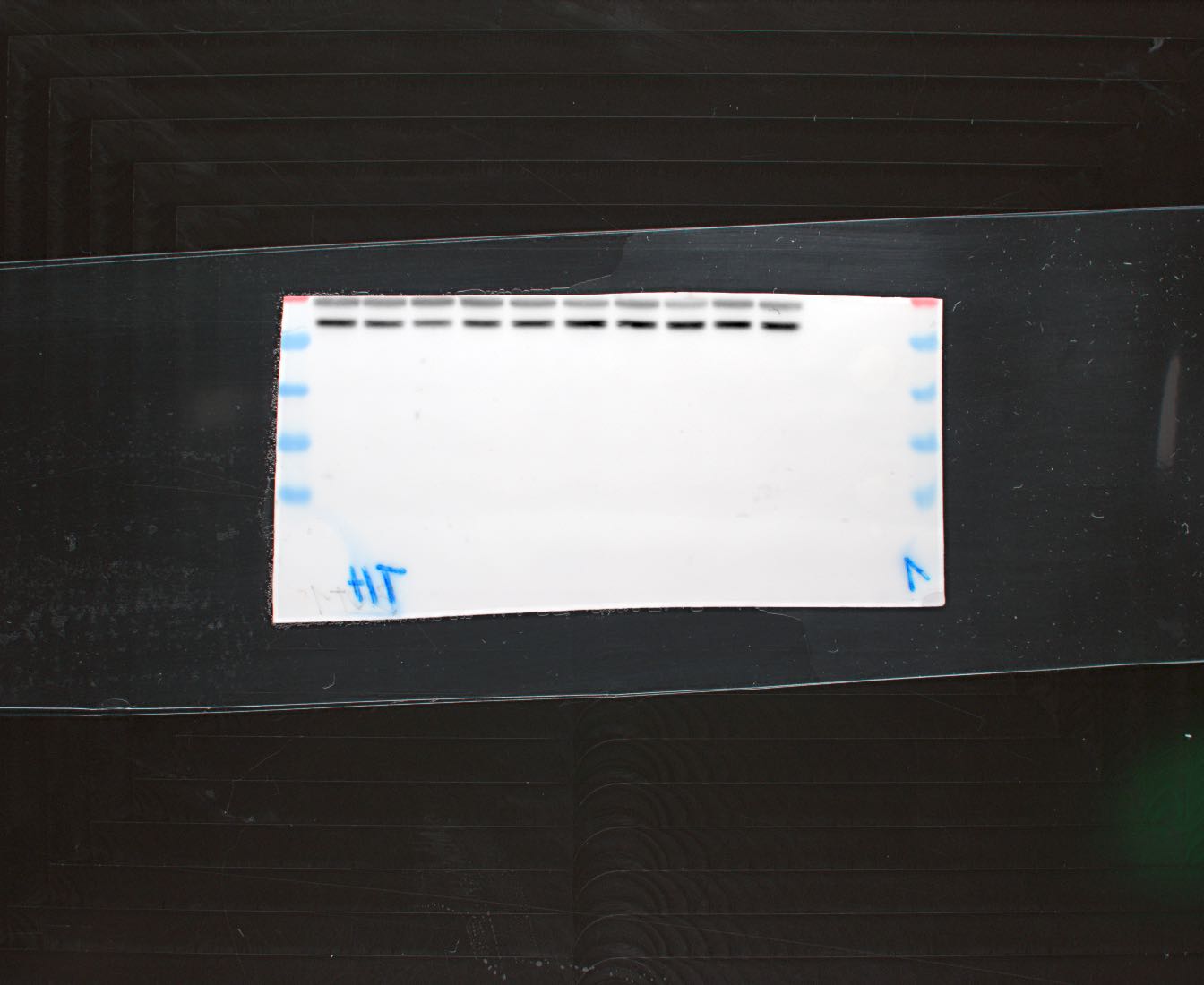

Supplement: Supplementary file 3 — Source Data for Expanded View and Appendix [file EMBR-21-e49807-s009.zip › Source_Data_appendix_figures/Source_Data_Appendix_FigS4/EMBOR-2019-49807V1_FigS4A_WB_TH.jpg]

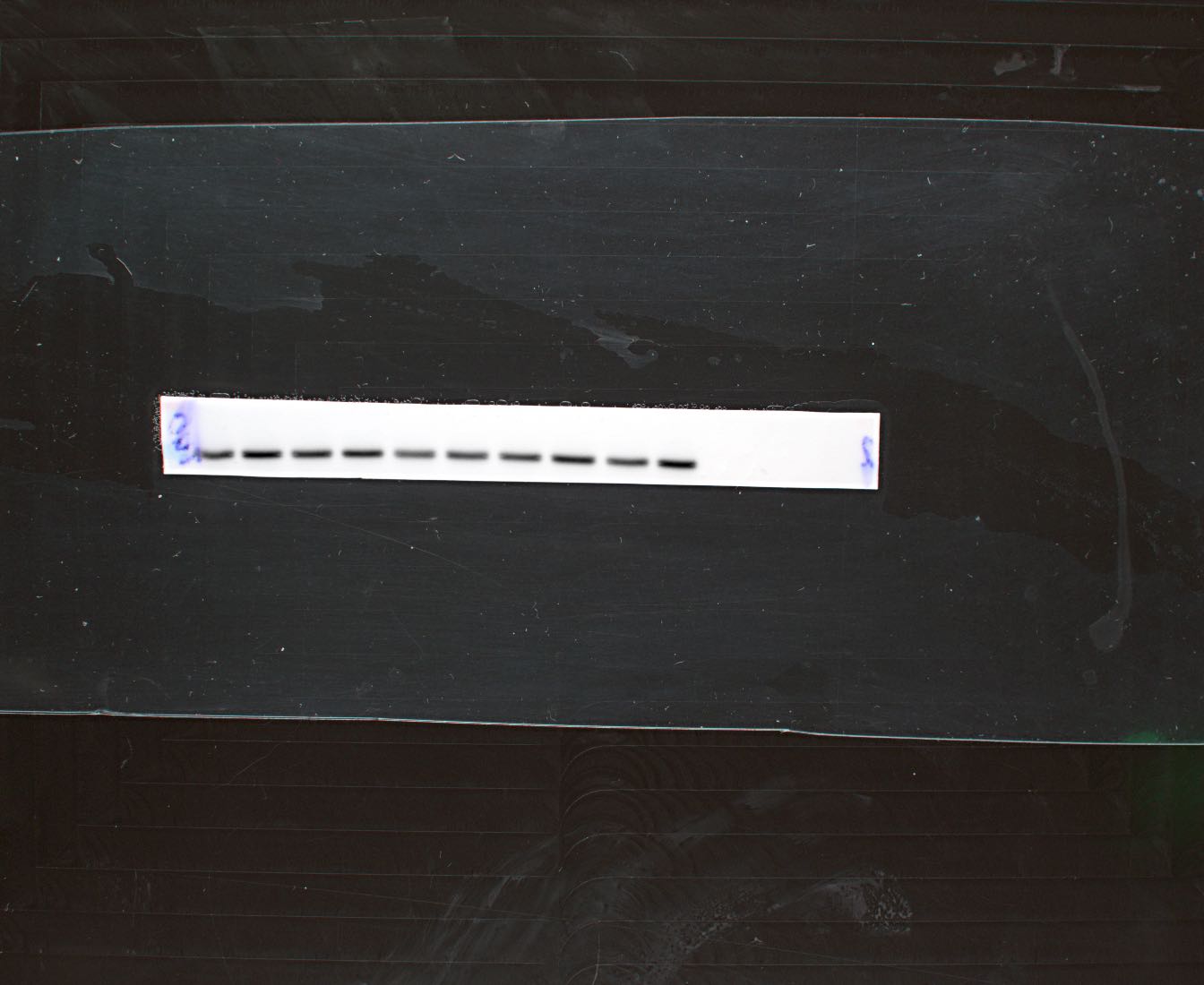

Supplement: Supplementary file 3 — Source Data for Expanded View and Appendix [file EMBR-21-e49807-s009.zip › Source_Data_appendix_figures/Source_Data_Appendix_FigS4/EMBOR-2019-49807V1_FigS4A_WB_TUBULIN.jpg]

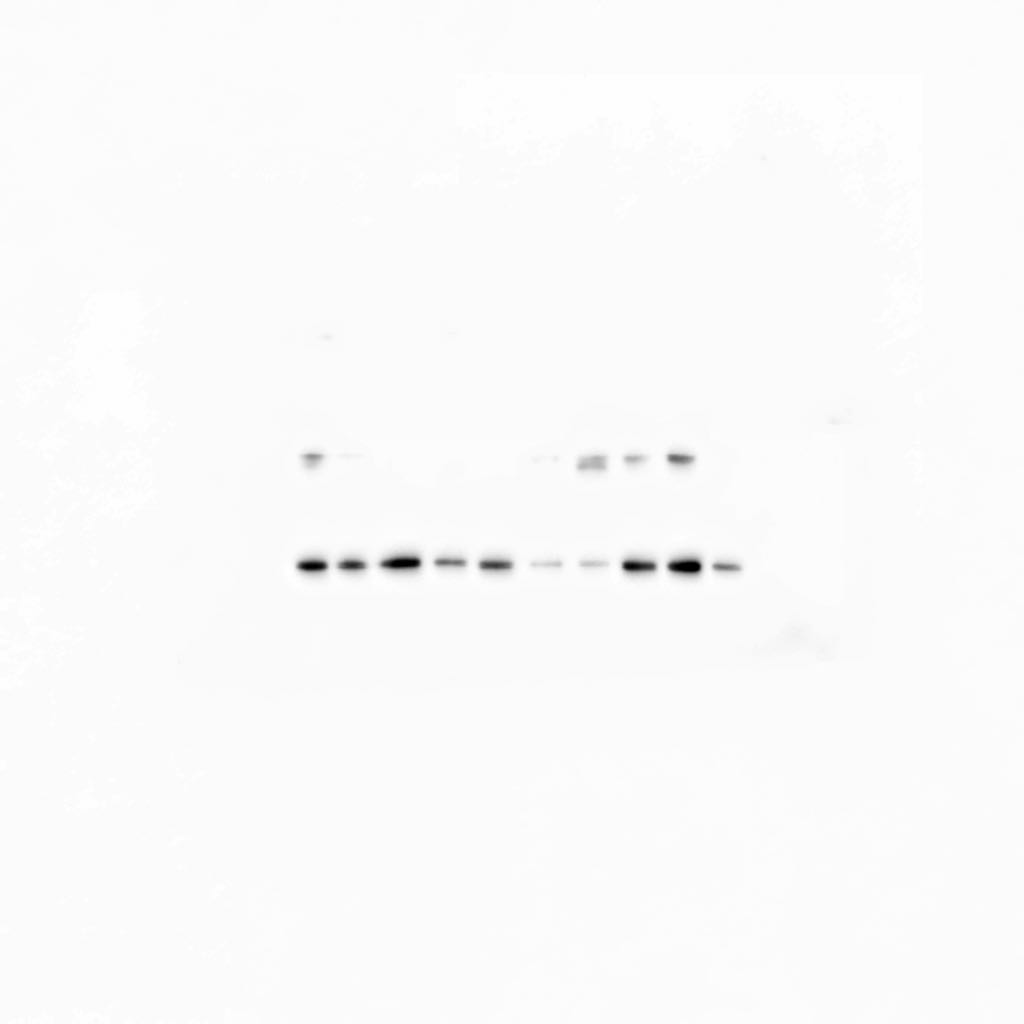

Supplement: Supplementary file 3 — Source Data for Expanded View and Appendix [file EMBR-21-e49807-s009.zip › Source_Data_appendix_figures/Source_Data_Appendix_FigS4/EMBOR-2019-49807V1_FigS4C_WB_GAPDH.jpg]

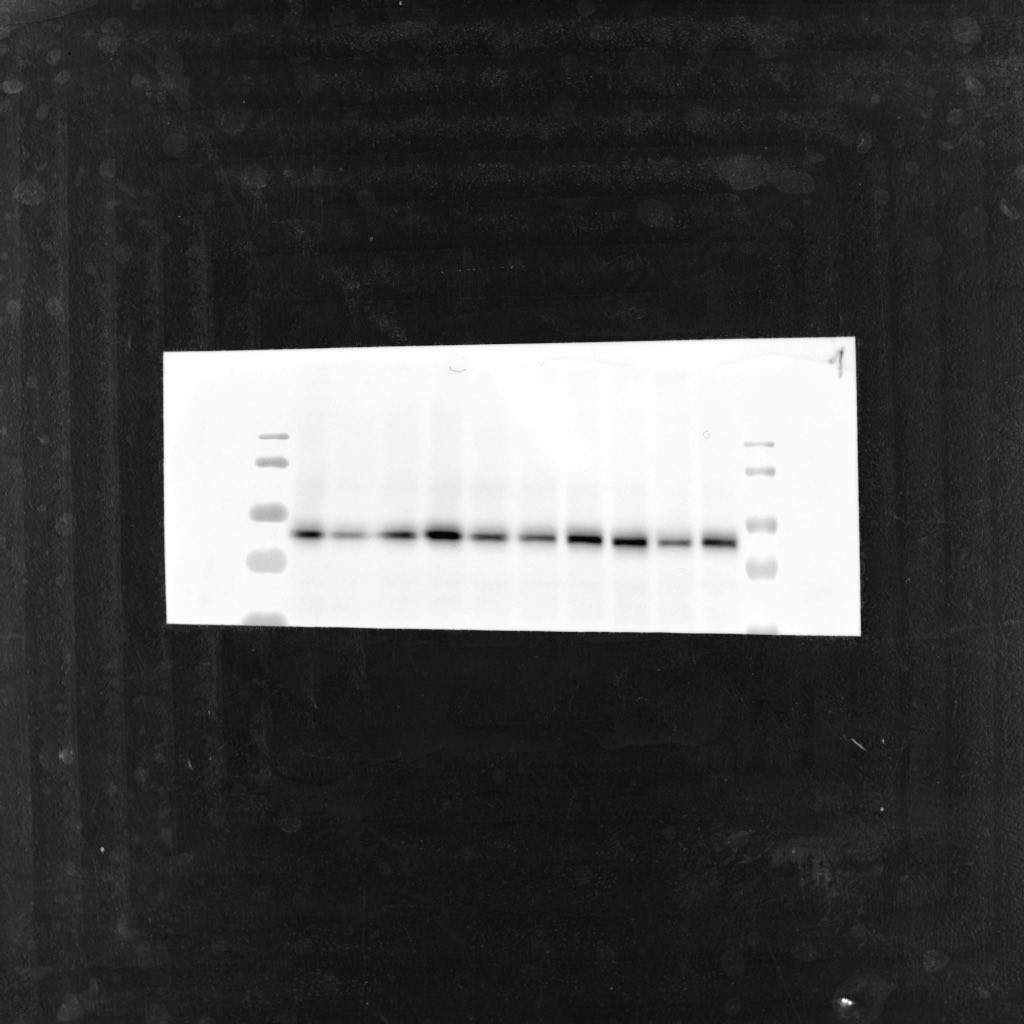

Supplement: Supplementary file 3 — Source Data for Expanded View and Appendix [file EMBR-21-e49807-s009.zip › Source_Data_appendix_figures/Source_Data_Appendix_FigS4/EMBOR-2019-49807V1_FigS4C_WB_PHSLS660.jpg]

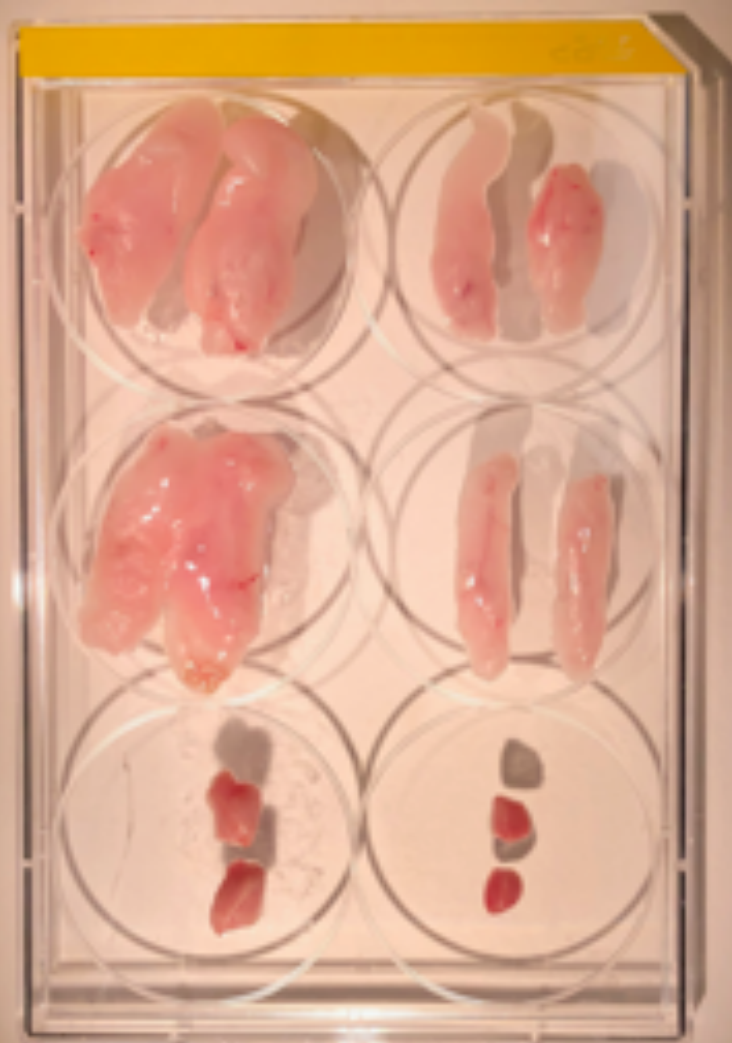

Supplement: Supplementary file 6 — Source Data for Figure 2 [file EMBR-21-e49807-s004.zip › EMBOR-2019-49807V1_Fig2A_BAT.pdf]

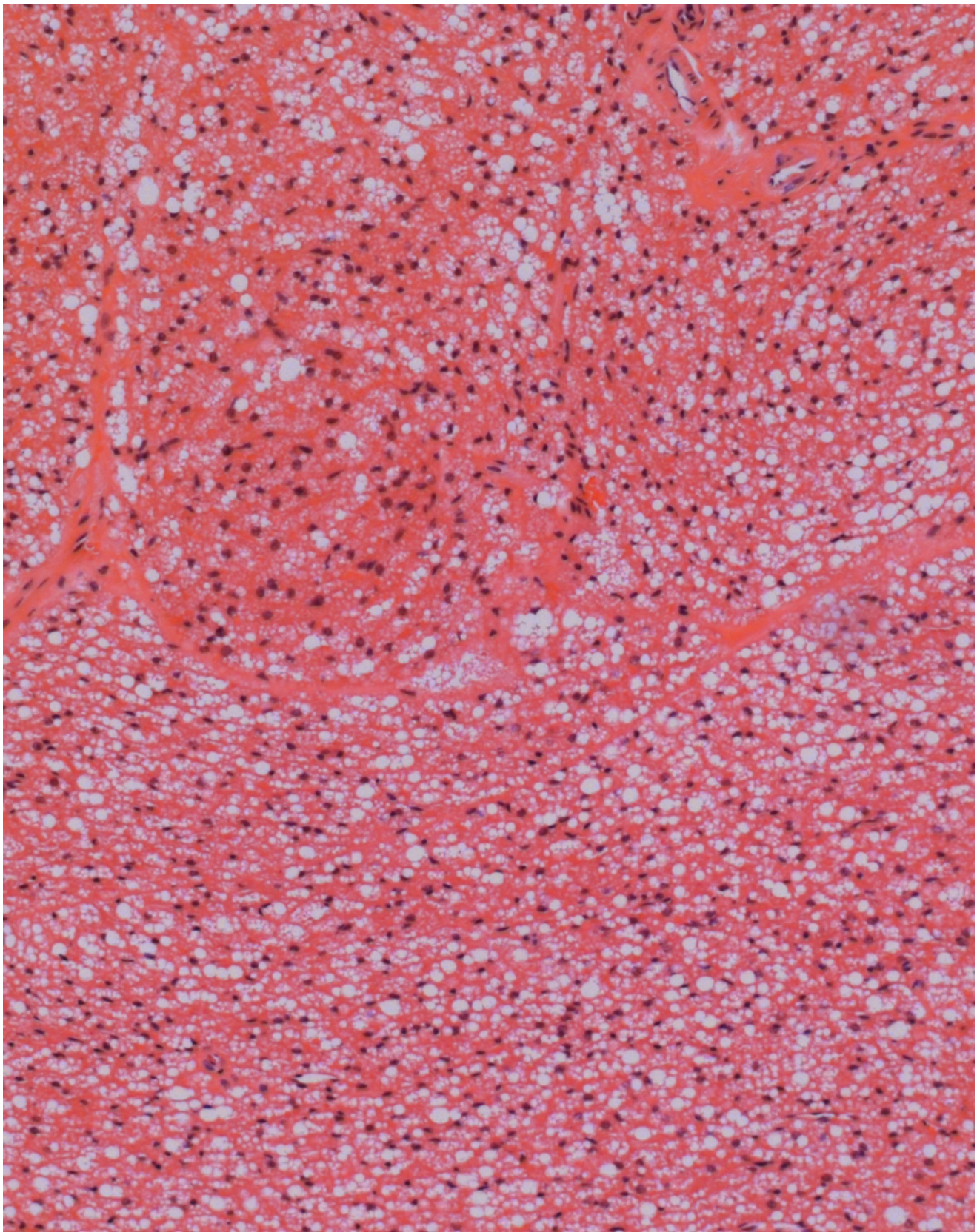

Supplement: Supplementary file 6 — Source Data for Figure 2 [file EMBR-21-e49807-s004.zip › EMBOR-2019-49807V1_Fig2C_HE_Cdk4KO.tif.pdf]

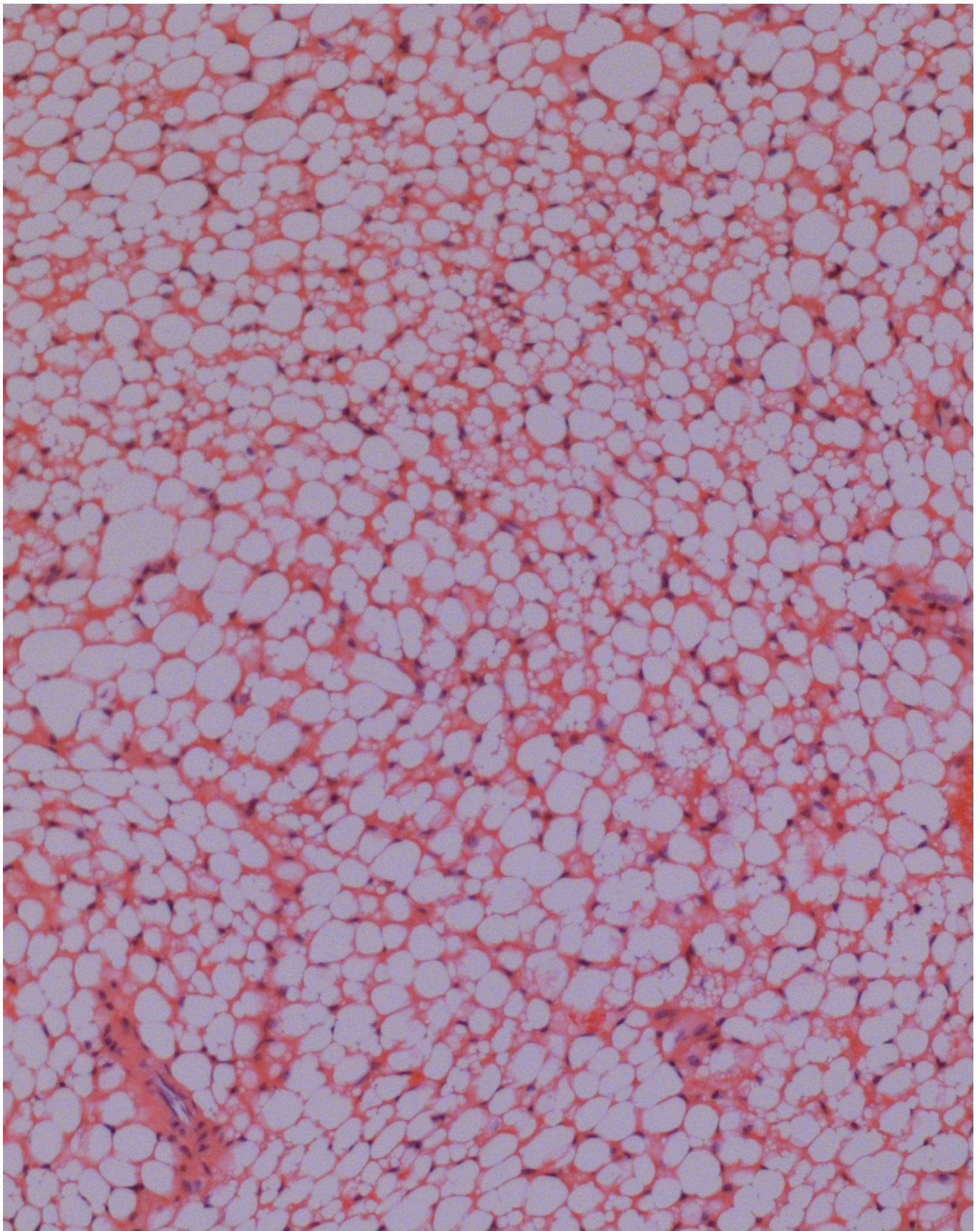

Supplement: Supplementary file 6 — Source Data for Figure 2 [file EMBR-21-e49807-s004.zip › EMBOR-2019-49807V1_Fig2C_HE_Cdk4WT.tif.pdf]

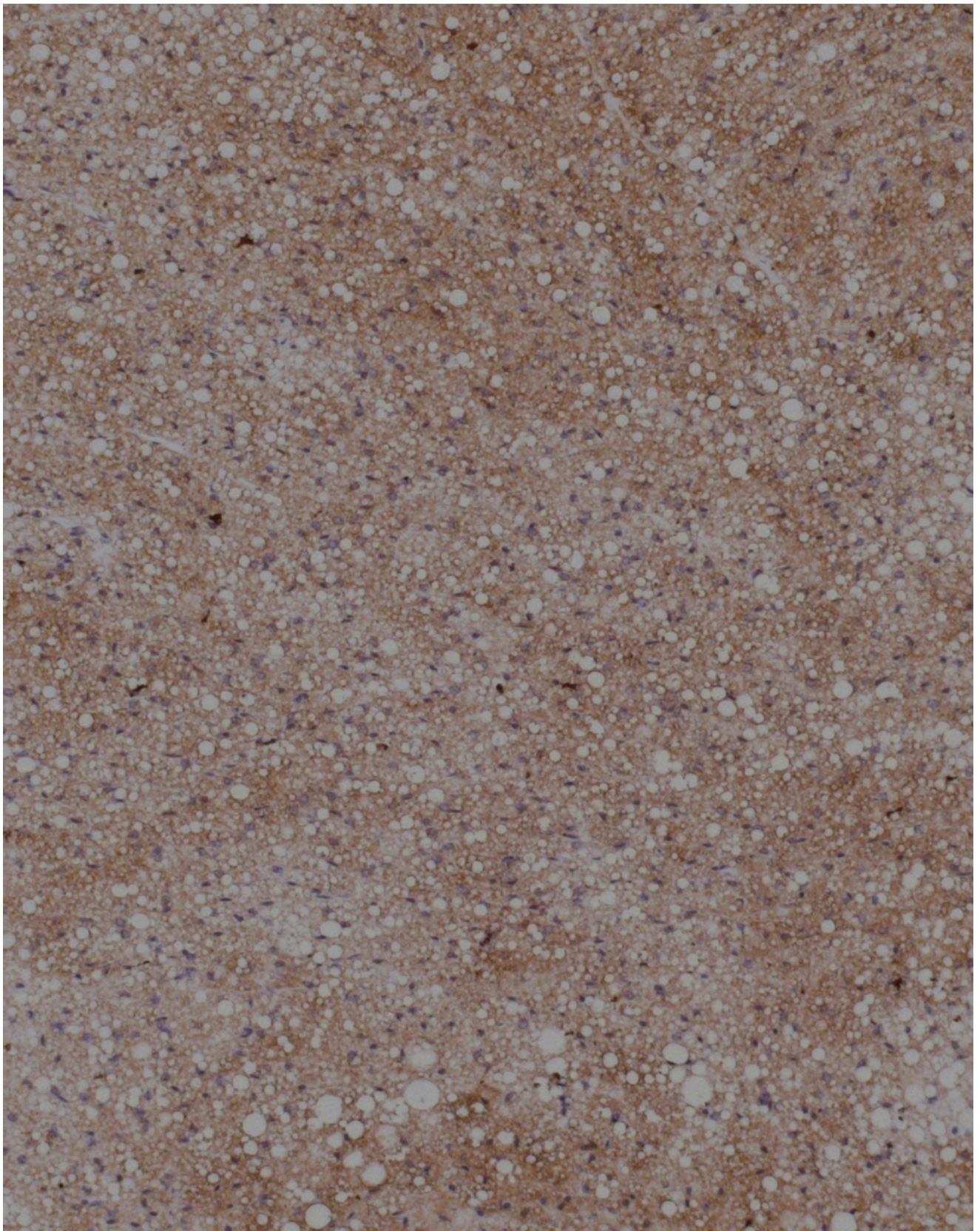

Supplement: Supplementary file 6 — Source Data for Figure 2 [file EMBR-21-e49807-s004.zip › EMBOR-2019-49807V1_Fig2C_UCP1_Cdk4KO.tif.pdf]

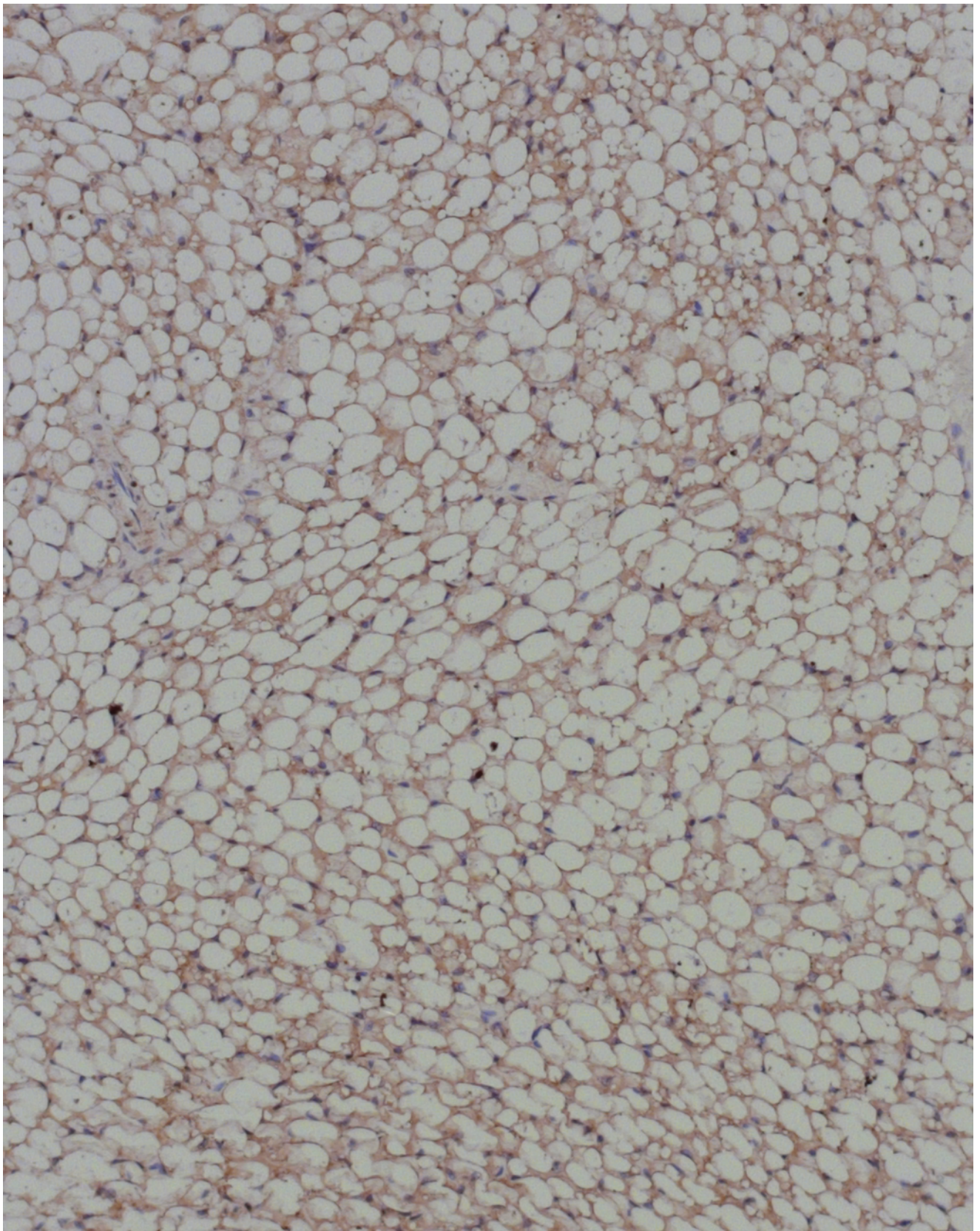

Supplement: Supplementary file 6 — Source Data for Figure 2 [file EMBR-21-e49807-s004.zip › EMBOR-2019-49807V1_Fig2C_UCP1_Cdk4WT.tif.pdf]

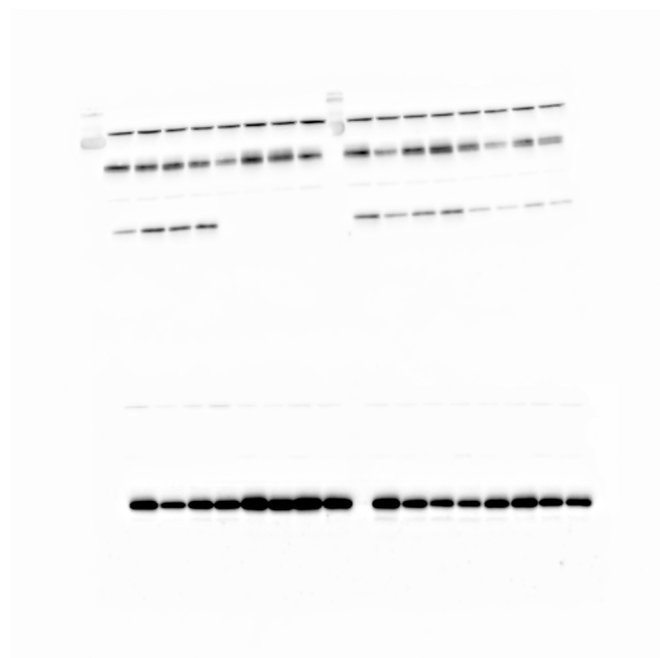

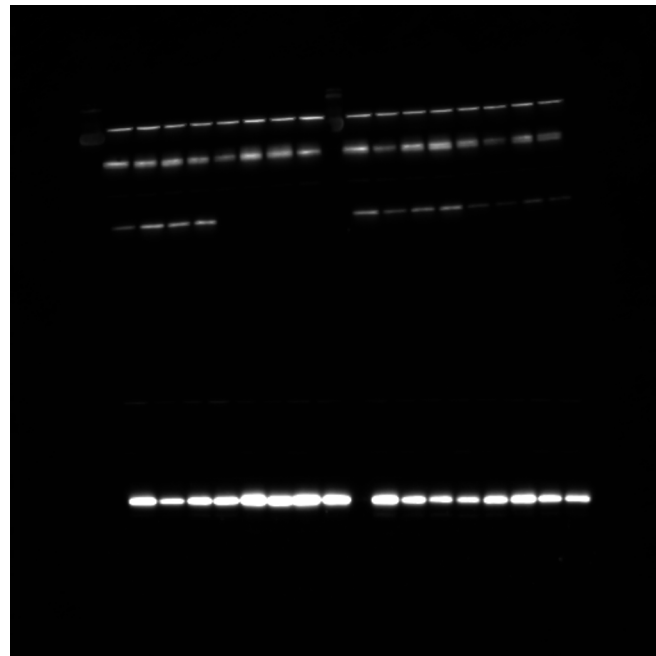

Supplement: Supplementary file 6 — Source Data for Figure 2 [file EMBR-21-e49807-s004.zip › EMBOR-2019-49807V1_Fig2D_WB_CDK4.Tif.pdf]

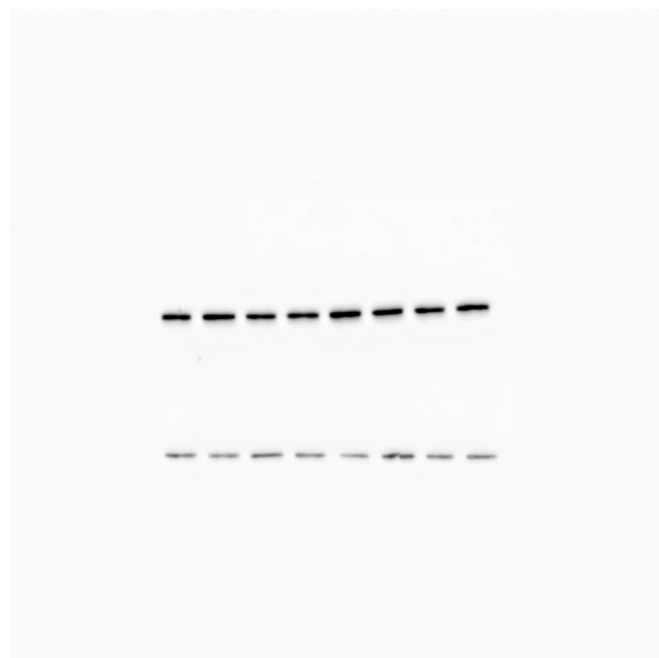

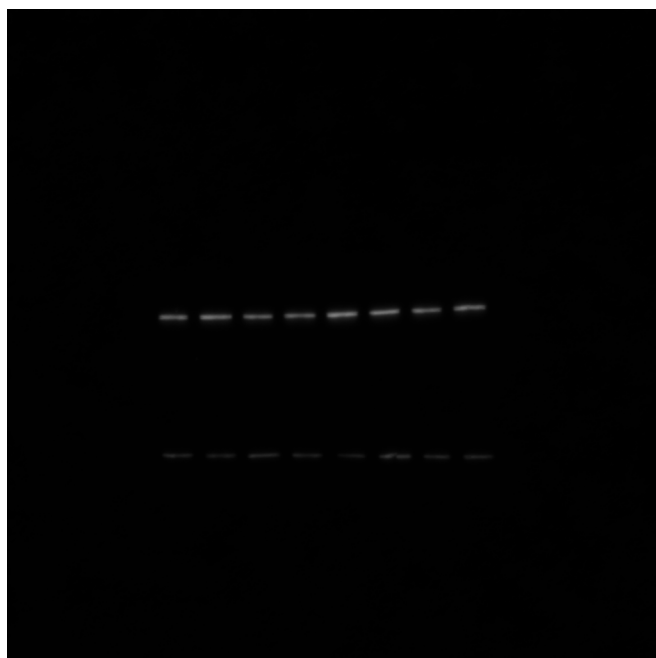

Supplement: Supplementary file 6 — Source Data for Figure 2 [file EMBR-21-e49807-s004.zip › EMBOR-2019-49807V1_Fig2D_WB_HSP90.Tif.pdf]

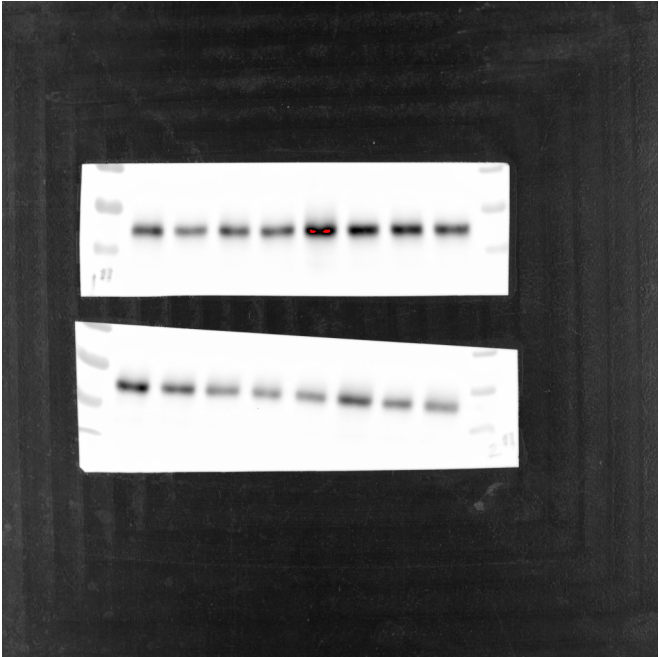

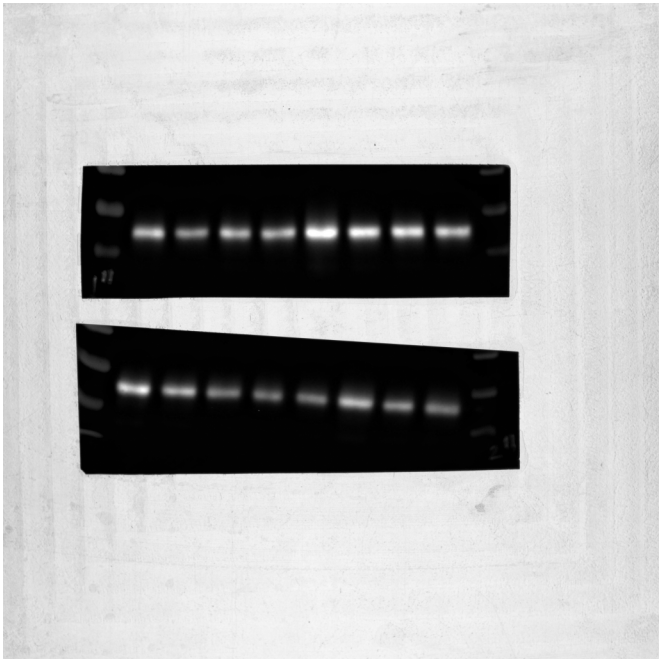

Supplement: Supplementary file 6 — Source Data for Figure 2 [file EMBR-21-e49807-s004.zip › EMBOR-2019-49807V1_Fig2D_WB_UCP1.Tif.pdf]

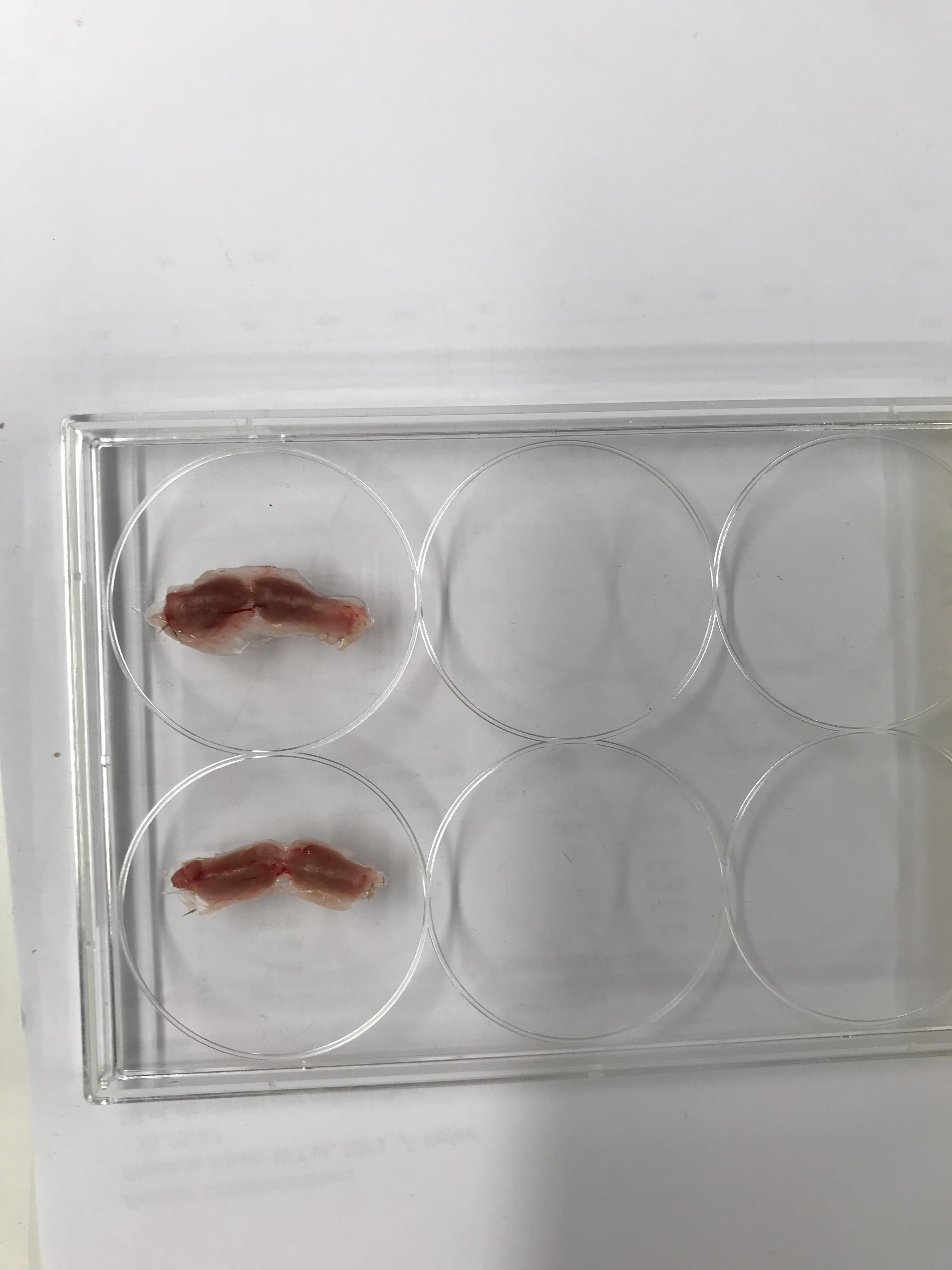

Supplement: Supplementary file 7 — Source Data for Figure 3 [file EMBR-21-e49807-s005.zip › EMBOR-2019-49807V1_Fig3C_BAT.jpg]

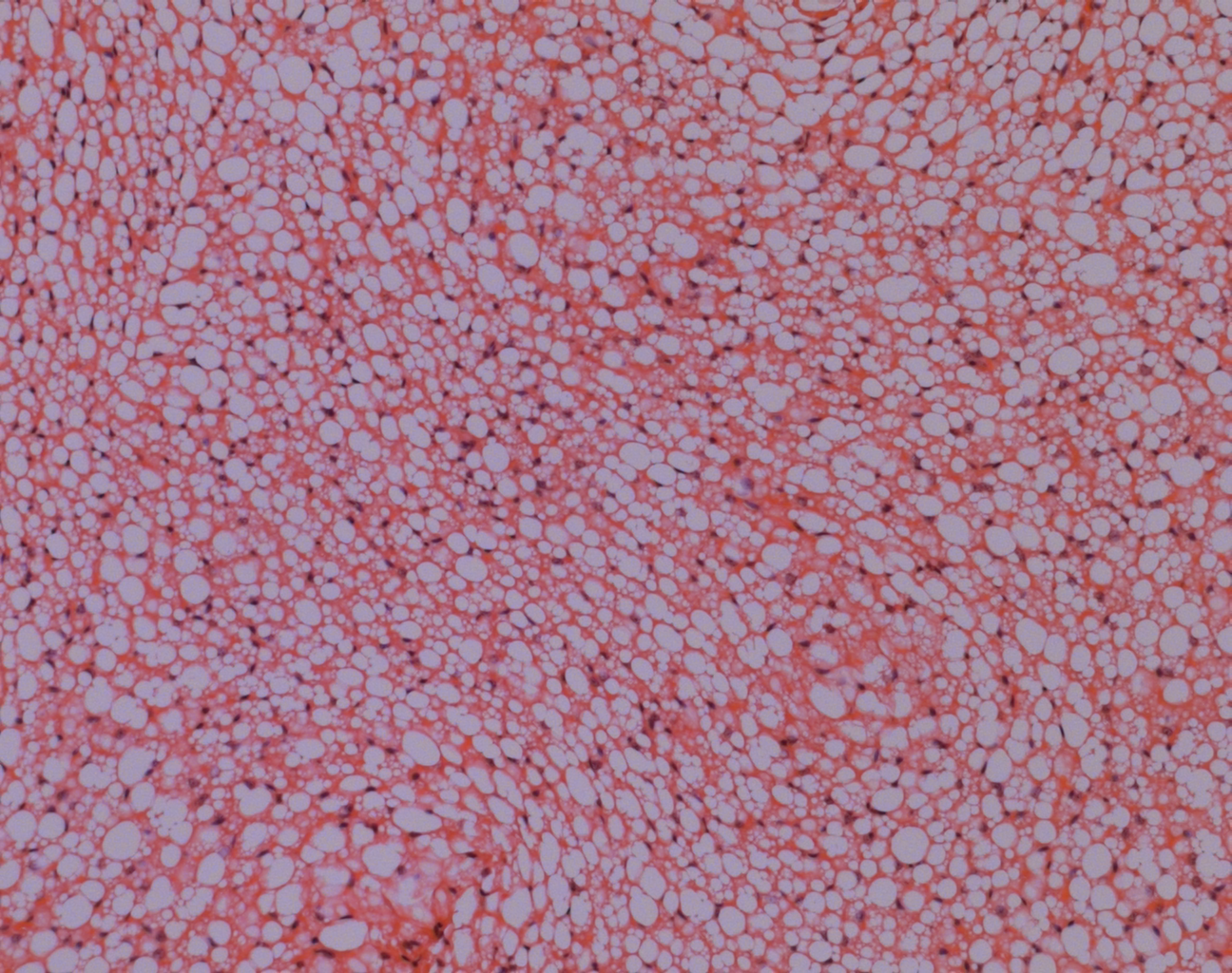

Supplement: Supplementary file 7 — Source Data for Figure 3 [file EMBR-21-e49807-s005.zip › EMBOR-2019-49807V1_Fig3E_HE_KO.tif]

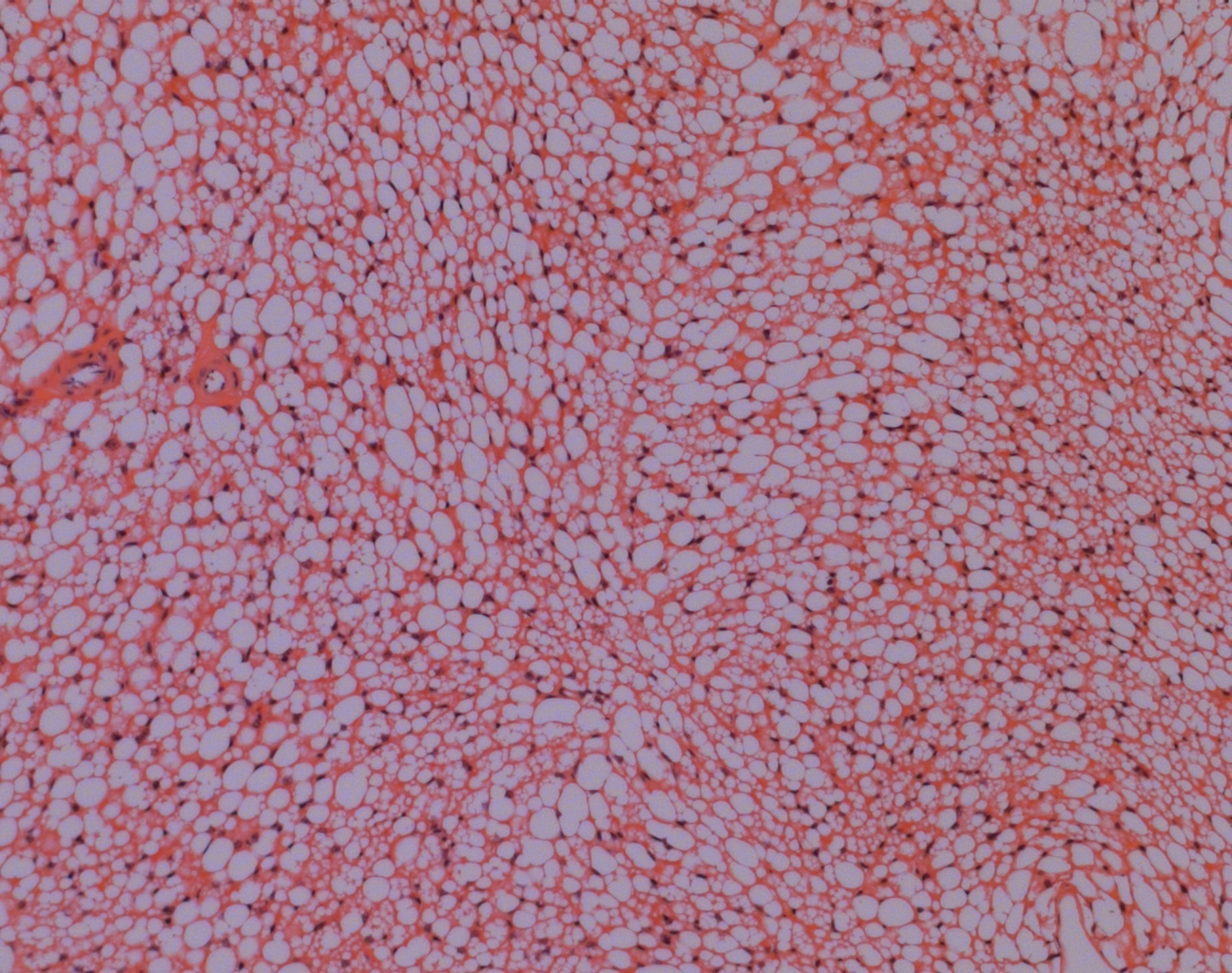

Supplement: Supplementary file 7 — Source Data for Figure 3 [file EMBR-21-e49807-s005.zip › EMBOR-2019-49807V1_Fig3E_HE_WT.tif]

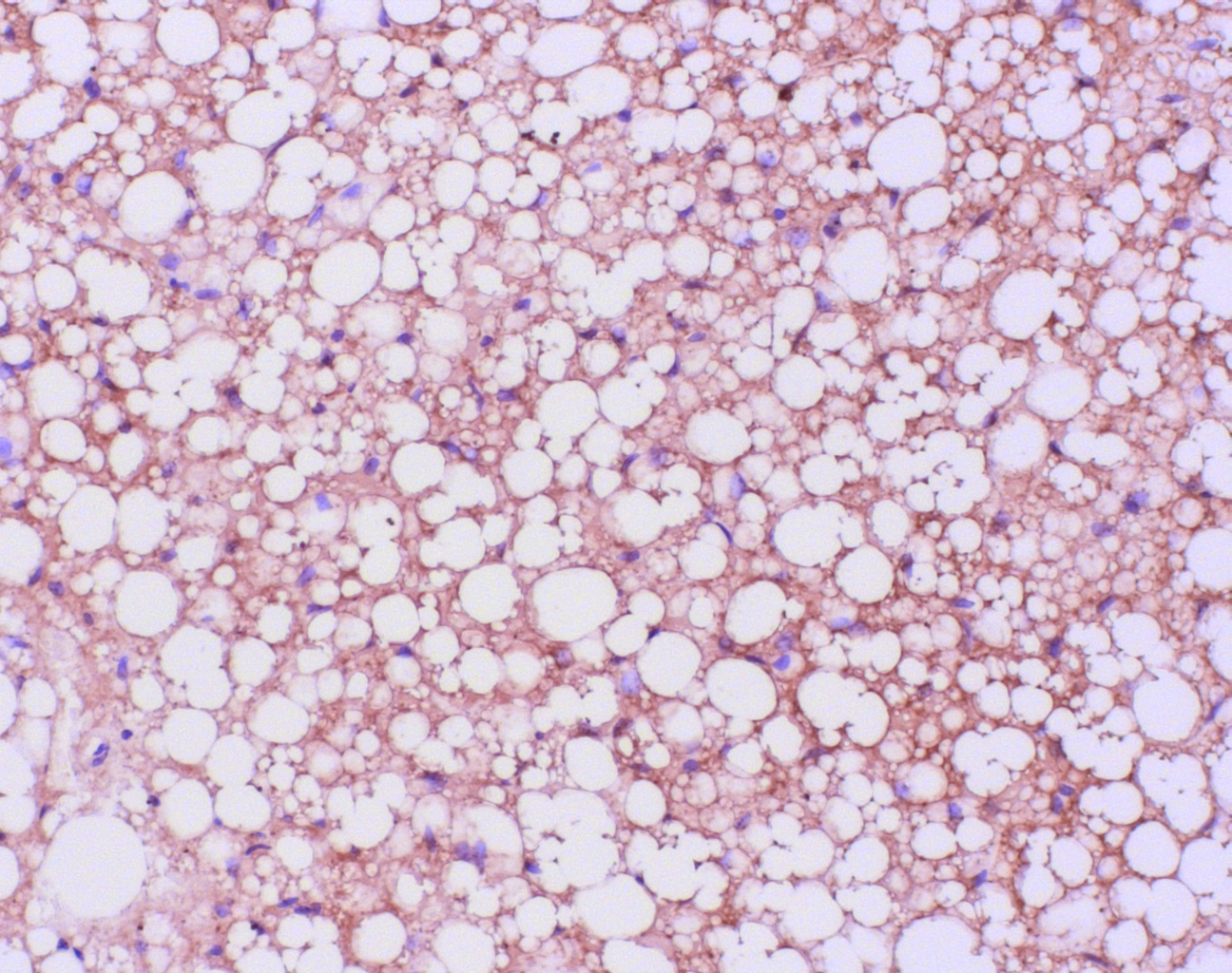

Supplement: Supplementary file 7 — Source Data for Figure 3 [file EMBR-21-e49807-s005.zip › EMBOR-2019-49807V1_Fig3E_UCP1_KO.tif]

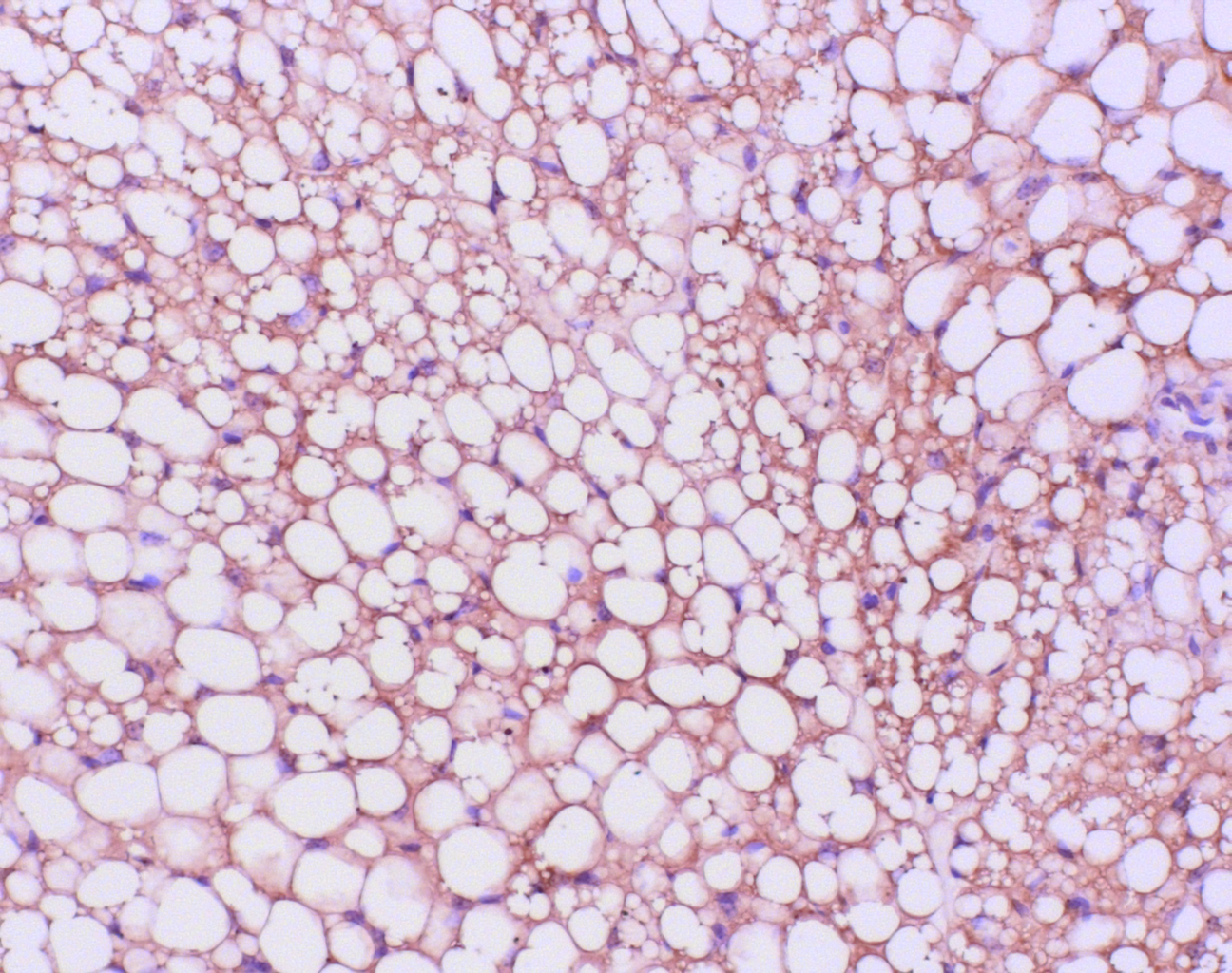

Supplement: Supplementary file 7 — Source Data for Figure 3 [file EMBR-21-e49807-s005.zip › EMBOR-2019-49807V1_Fig3E_UCP1_WT.tif]

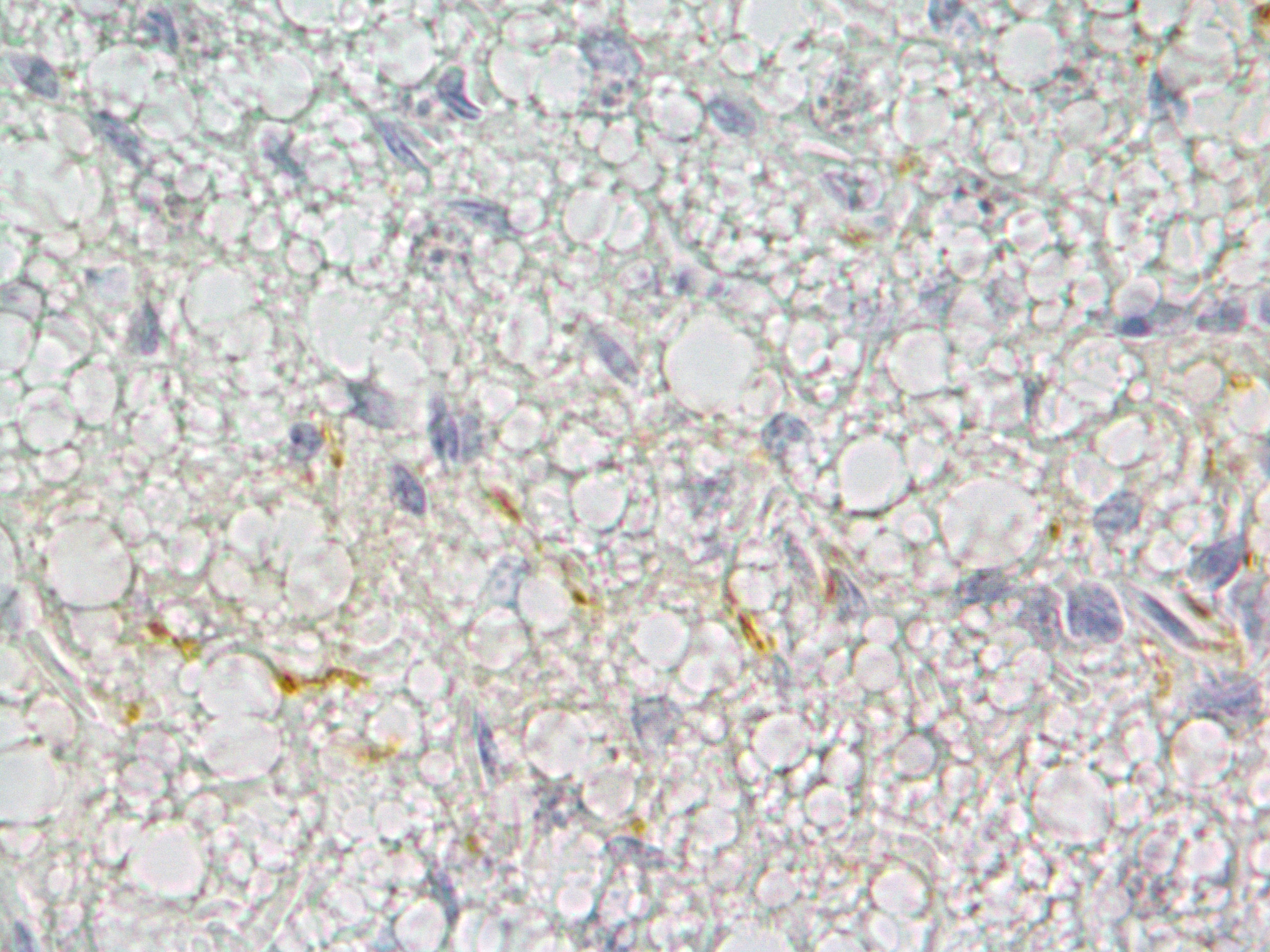

Supplement: Supplementary file 8 — Source Data for Figure 4 [file EMBR-21-e49807-s006.zip › EMBOR-2019-49807V1_Fig4A_TH_KO.JPG]

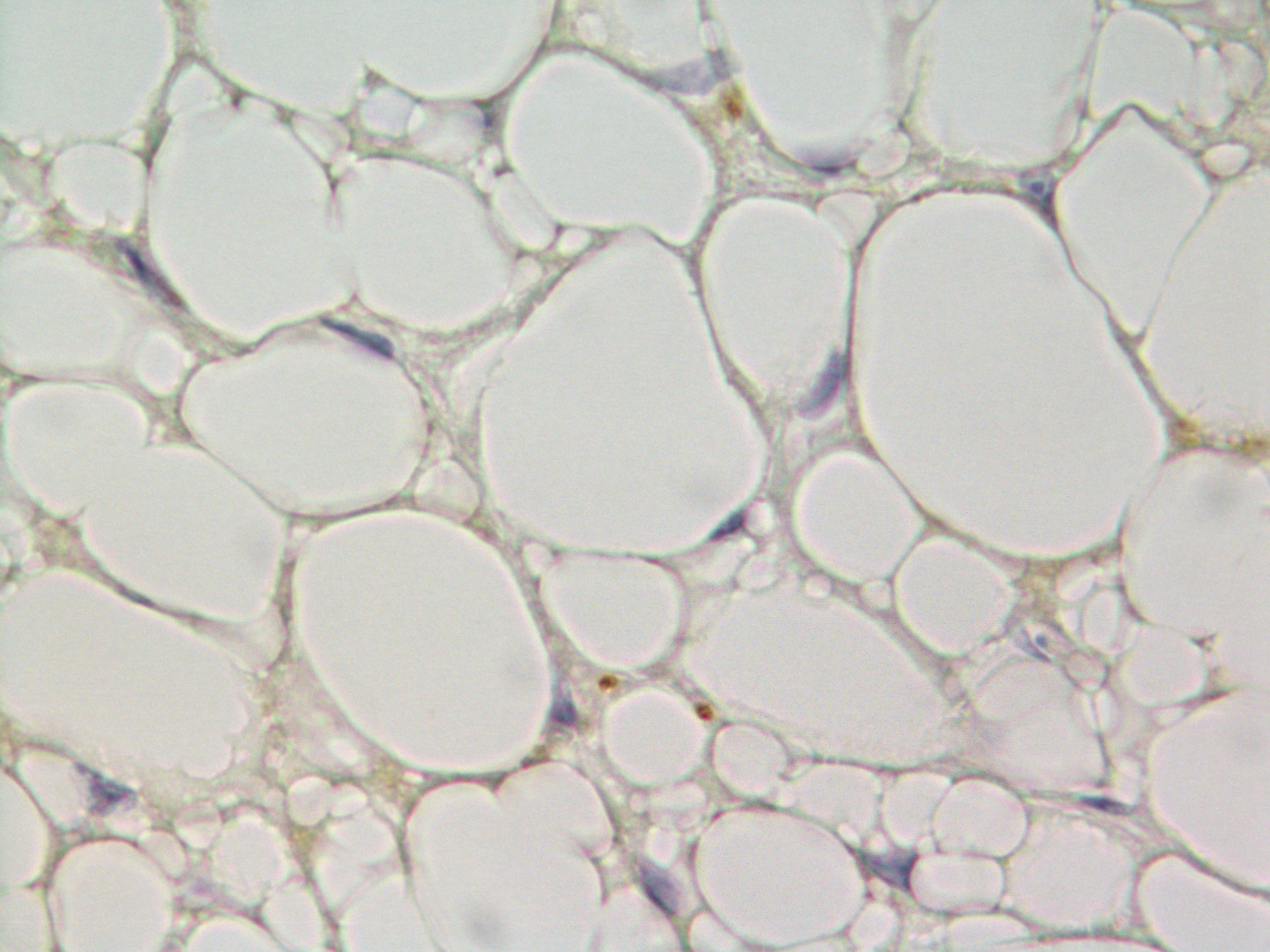

Supplement: Supplementary file 8 — Source Data for Figure 4 [file EMBR-21-e49807-s006.zip › EMBOR-2019-49807V1_Fig4A_TH_WT.JPG]

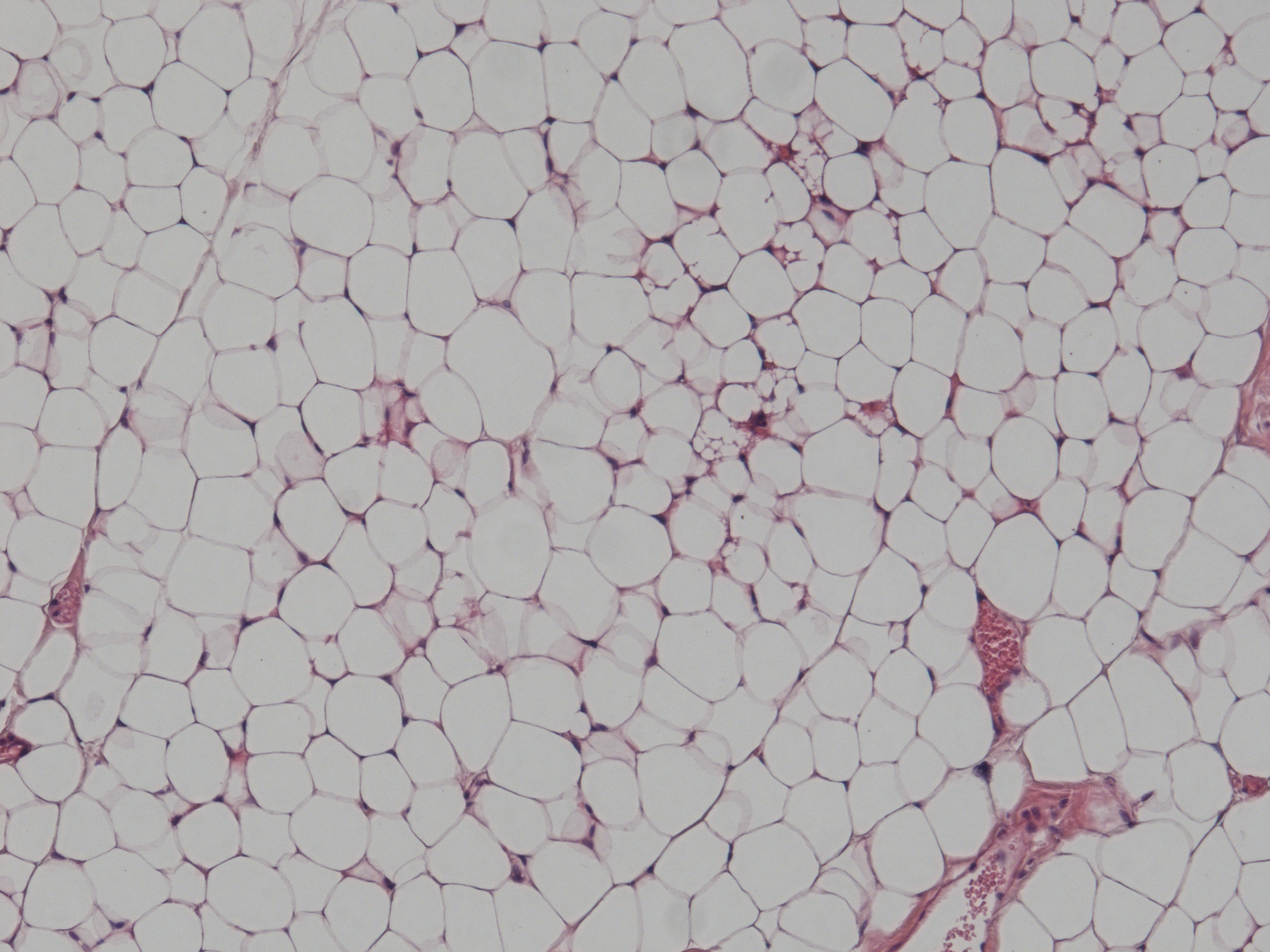

Supplement: Supplementary file 8 — Source Data for Figure 4 [file EMBR-21-e49807-s006.zip › EMBOR-2019-49807V1_Fig4C_HE_KO.tif]

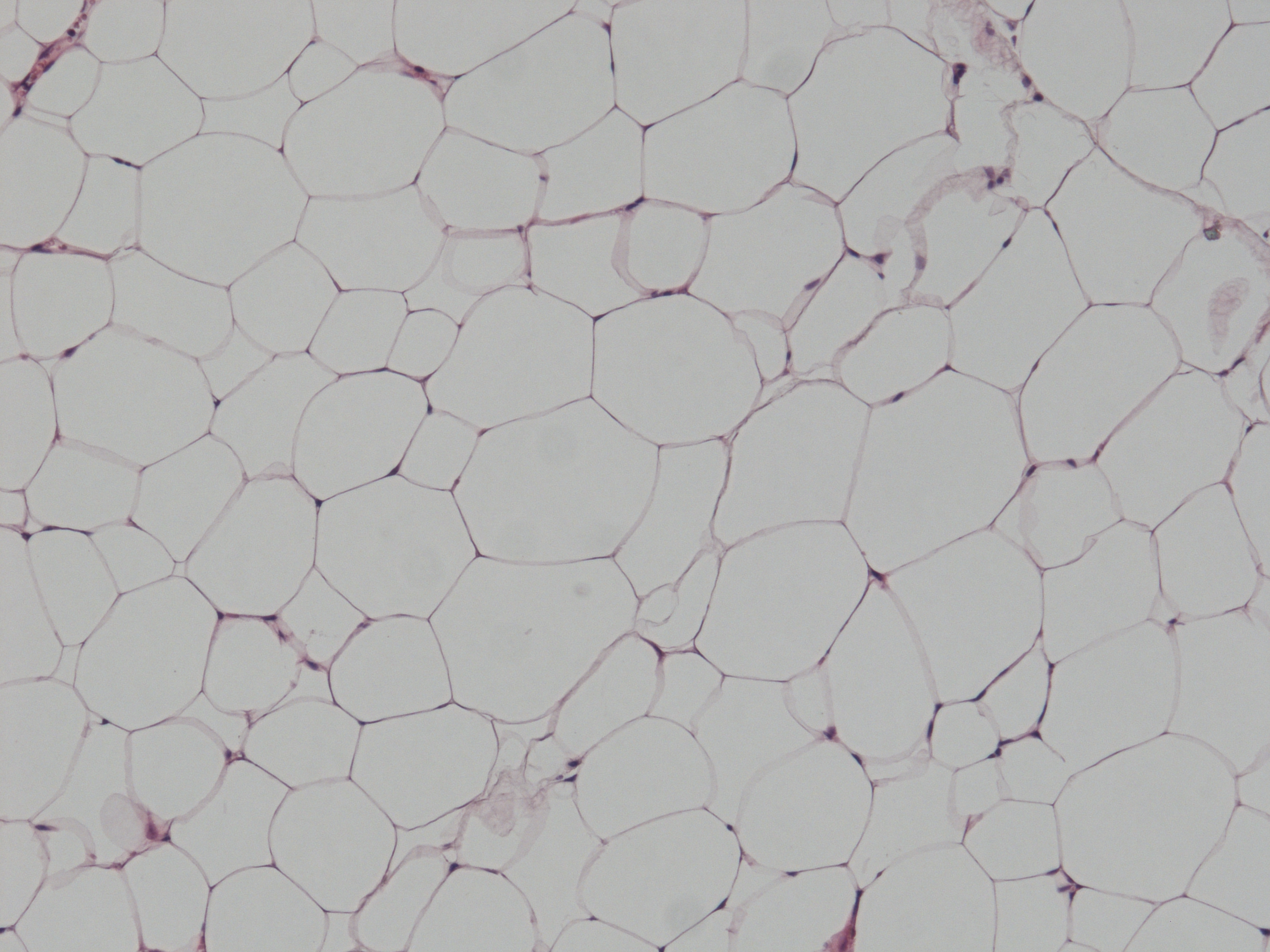

Supplement: Supplementary file 8 — Source Data for Figure 4 [file EMBR-21-e49807-s006.zip › EMBOR-2019-49807V1_Fig4C_HE_WT.tif]

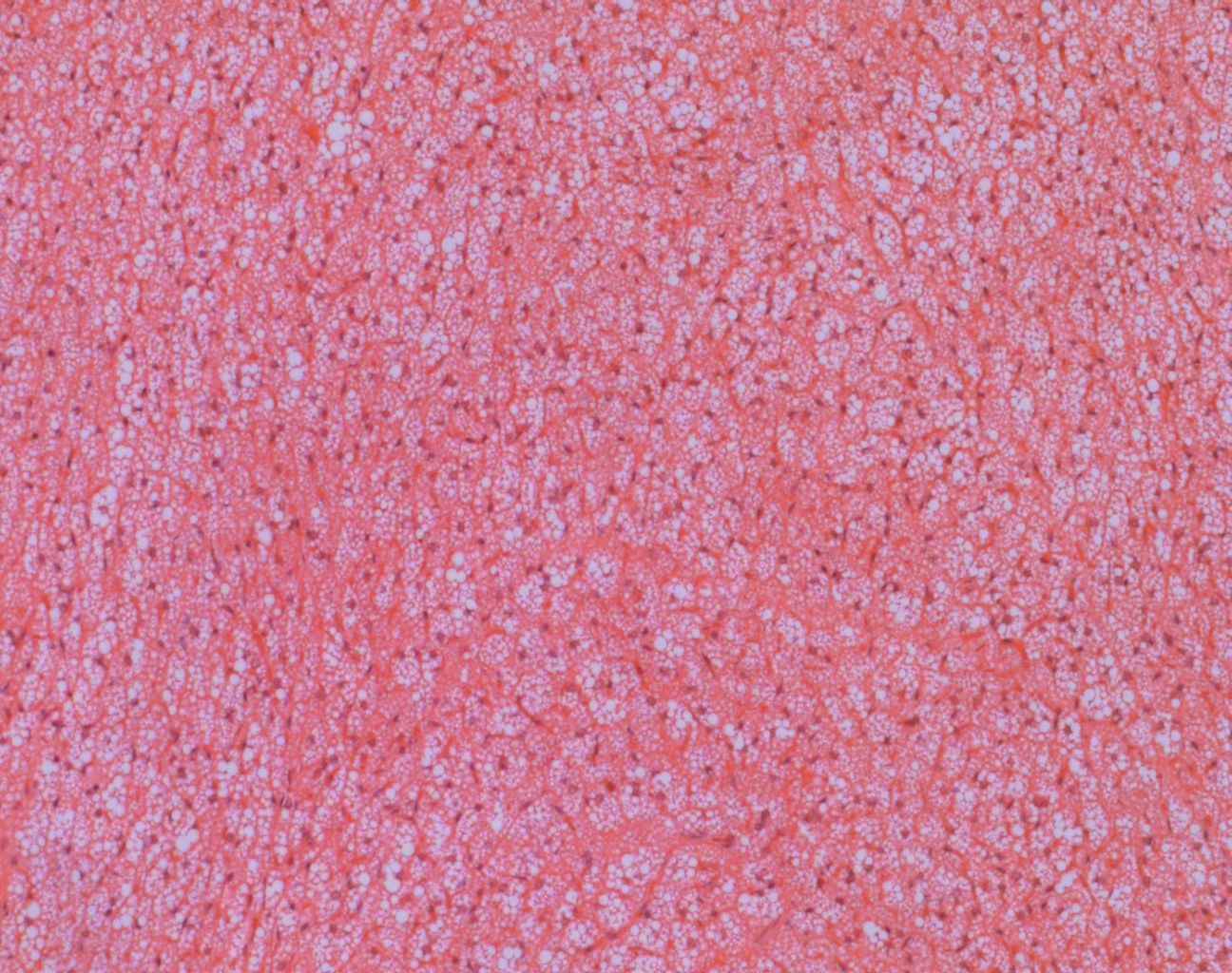

Supplement: Supplementary file 9 — Source Data for Figure 5 [file EMBR-21-e49807-s007.zip › EMBOR-2019-49807V1_Fig5D_HE_KO.tiff]

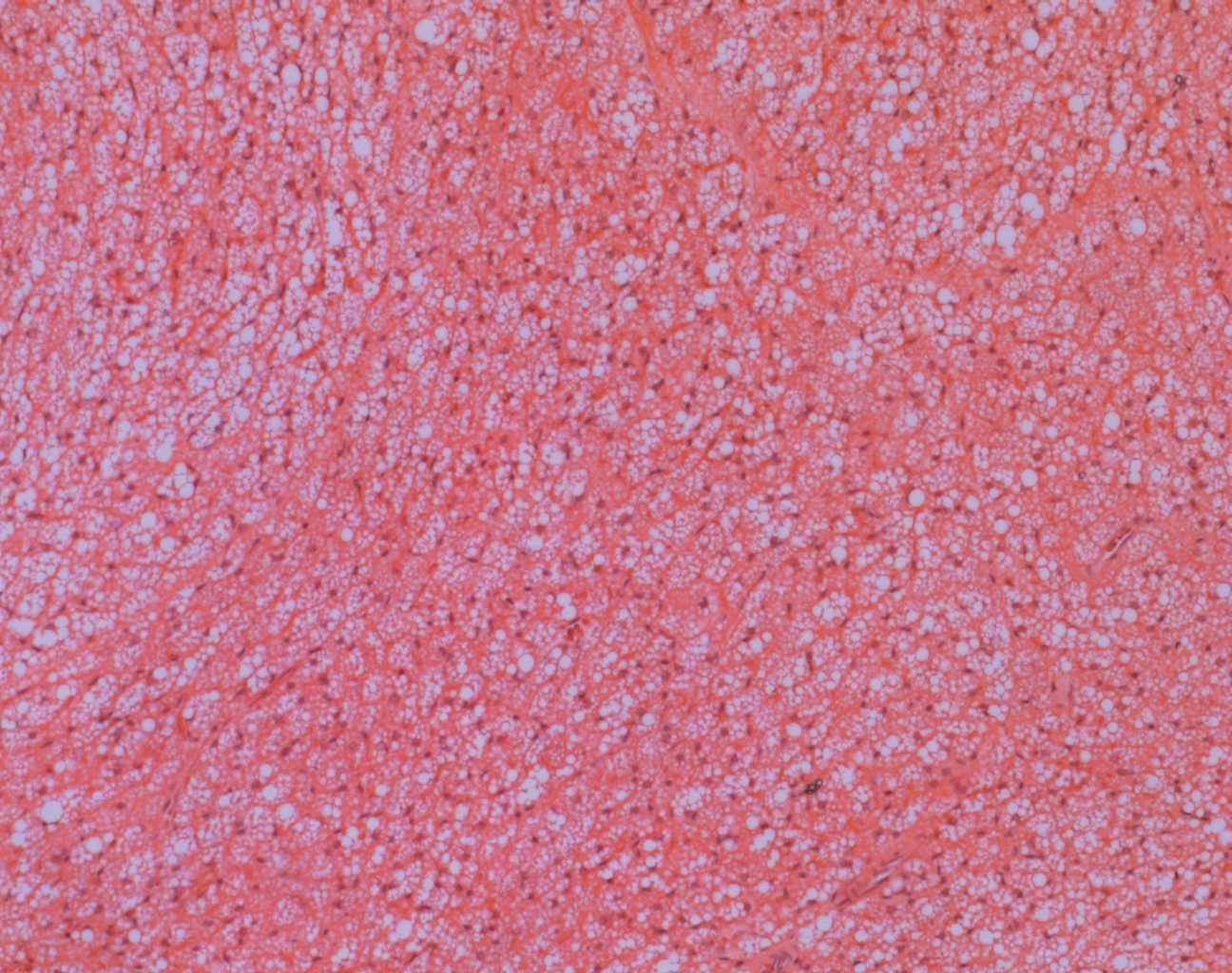

Supplement: Supplementary file 9 — Source Data for Figure 5 [file EMBR-21-e49807-s007.zip › EMBOR-2019-49807V1_Fig5D_HE_WT.tif]

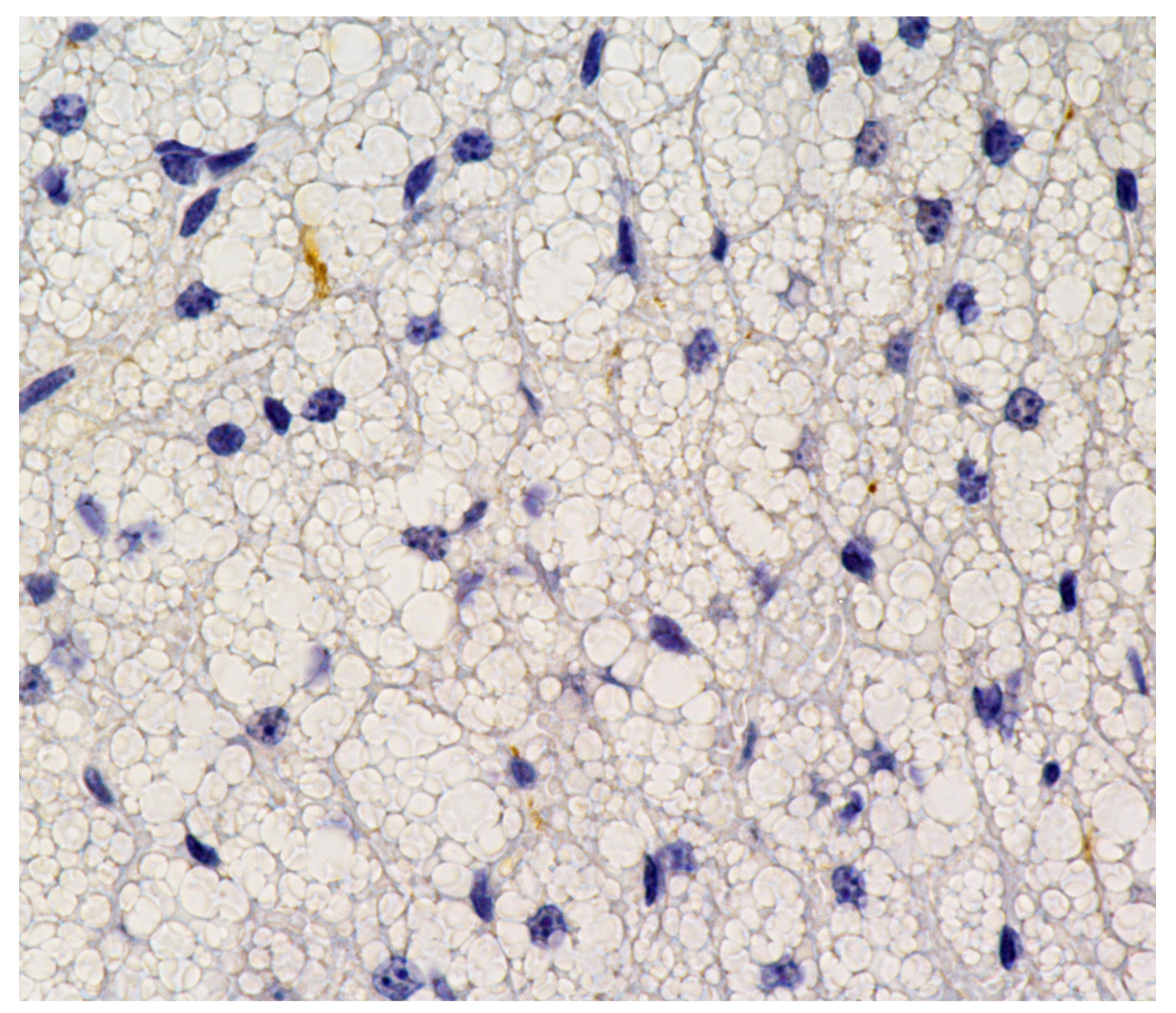

Supplement: Supplementary file 9 — Source Data for Figure 5 [file EMBR-21-e49807-s007.zip › EMBOR-2019-49807V1_Fig5F_TH_KO.jpg]

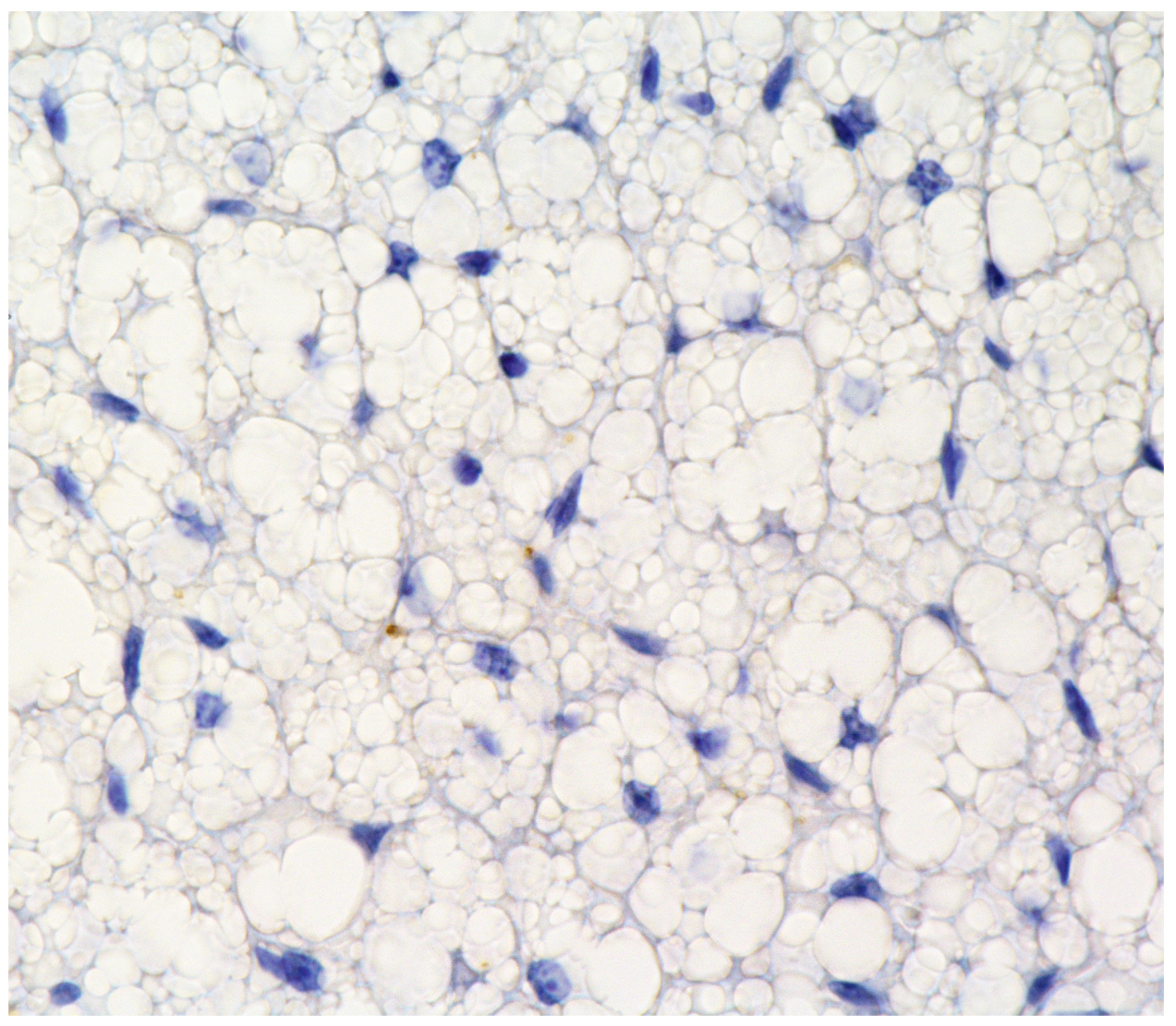

Supplement: Supplementary file 9 — Source Data for Figure 5 [file EMBR-21-e49807-s007.zip › EMBOR-2019-49807V1_Fig5F_TH_WTjpg.jpg]

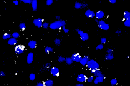

Supplement: Supplementary file 10 — Source Data for Figure 6 [file EMBR-21-e49807-s008.zip › EMBOR-2019-49807V1_Fig6C6_MERGE_WT.tif]

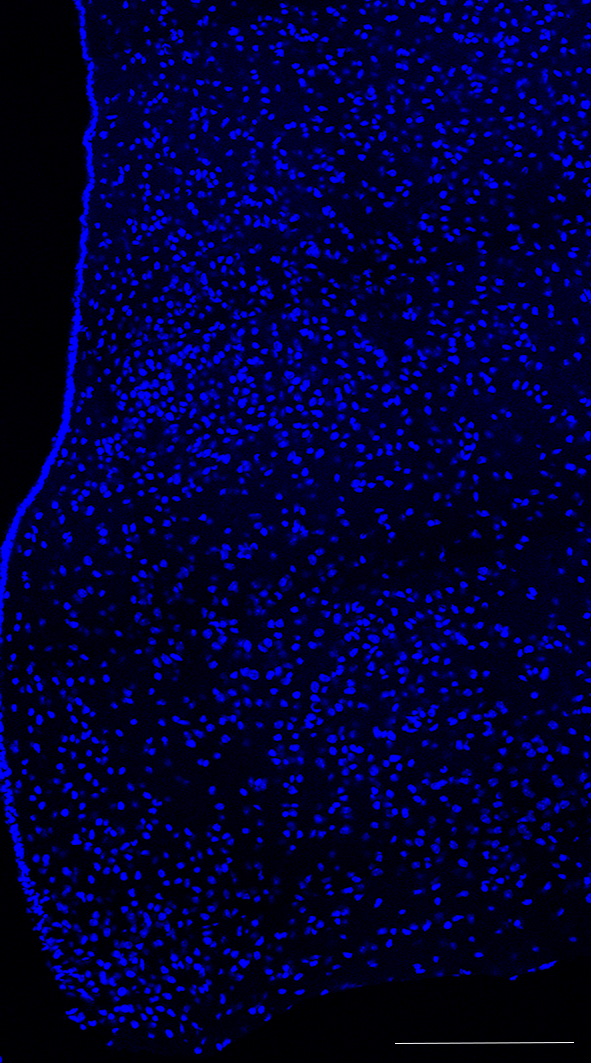

Supplement: Supplementary file 10 — Source Data for Figure 6 [file EMBR-21-e49807-s008.zip › EMBOR-2019-49807V1_Fig6D1_HOECHST_KO.tif]

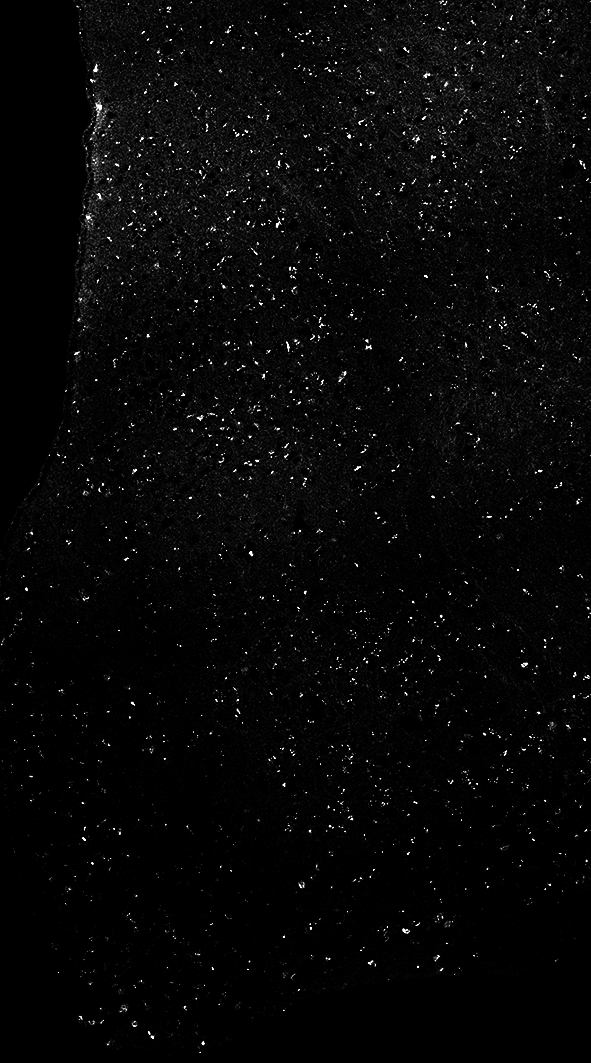

Supplement: Supplementary file 10 — Source Data for Figure 6 [file EMBR-21-e49807-s008.zip › EMBOR-2019-49807V1_Fig6D2_FOS_KO.tif]

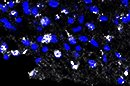

Supplement: Supplementary file 10 — Source Data for Figure 6 [file EMBR-21-e49807-s008.zip › EMBOR-2019-49807V1_Fig6D3_MERGE_KO.tif]

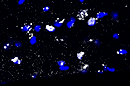

Supplement: Supplementary file 10 — Source Data for Figure 6 [file EMBR-21-e49807-s008.zip › EMBOR-2019-49807V1_Fig6D4_MERGE_KO.tif]

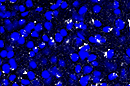

Supplement: Supplementary file 10 — Source Data for Figure 6 [file EMBR-21-e49807-s008.zip › EMBOR-2019-49807V1_Fig6D5_MERGE_KO.tif]

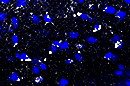

Supplement: Supplementary file 10 — Source Data for Figure 6 [file EMBR-21-e49807-s008.zip › EMBOR-2019-49807V1_Fig6D6_MERGE_KO.tif]

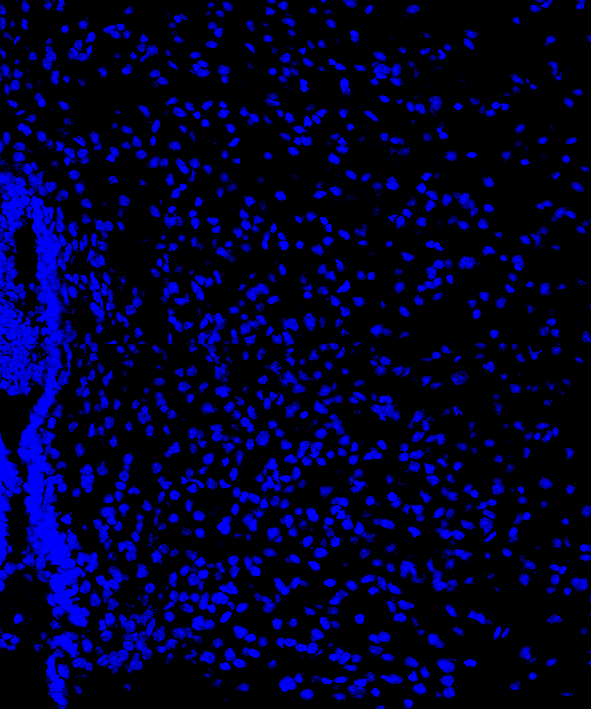

Supplement: Supplementary file 10 — Source Data for Figure 6 [file EMBR-21-e49807-s008.zip › EMBOR-2019-49807V1_Fig6A1_HOECHST_WT.tif]

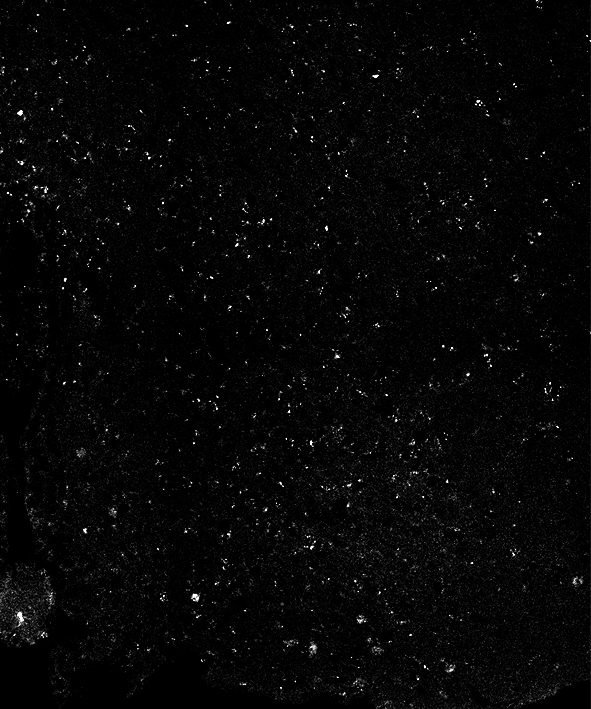

Supplement: Supplementary file 10 — Source Data for Figure 6 [file EMBR-21-e49807-s008.zip › EMBOR-2019-49807V1_Fig6A2_FOS_WT.tif]

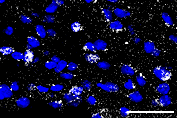

Supplement: Supplementary file 10 — Source Data for Figure 6 [file EMBR-21-e49807-s008.zip › EMBOR-2019-49807V1_Fig6A3_MERGE_WT.tif]

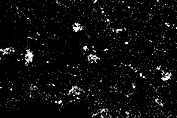

Supplement: Supplementary file 10 — Source Data for Figure 6 [file EMBR-21-e49807-s008.zip › EMBOR-2019-49807V1_Fig6A4_FOS_WT.tif]

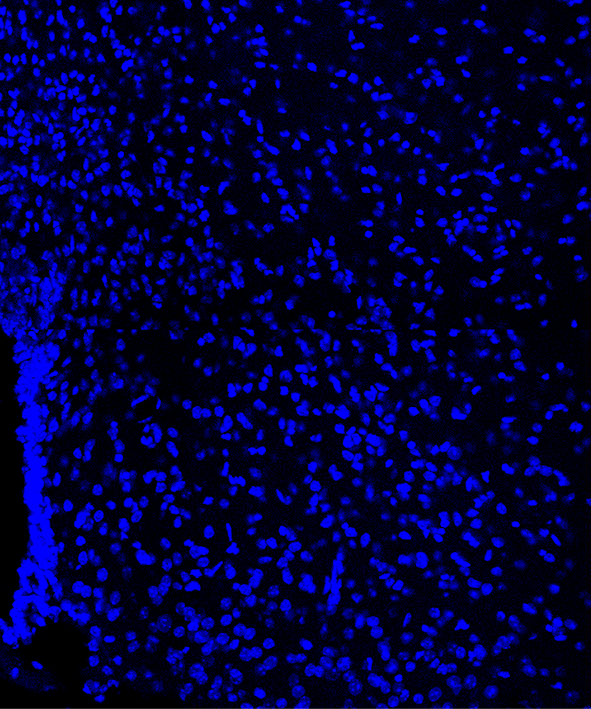

Supplement: Supplementary file 10 — Source Data for Figure 6 [file EMBR-21-e49807-s008.zip › EMBOR-2019-49807V1_Fig6B1_HOECHST_KO.tif]

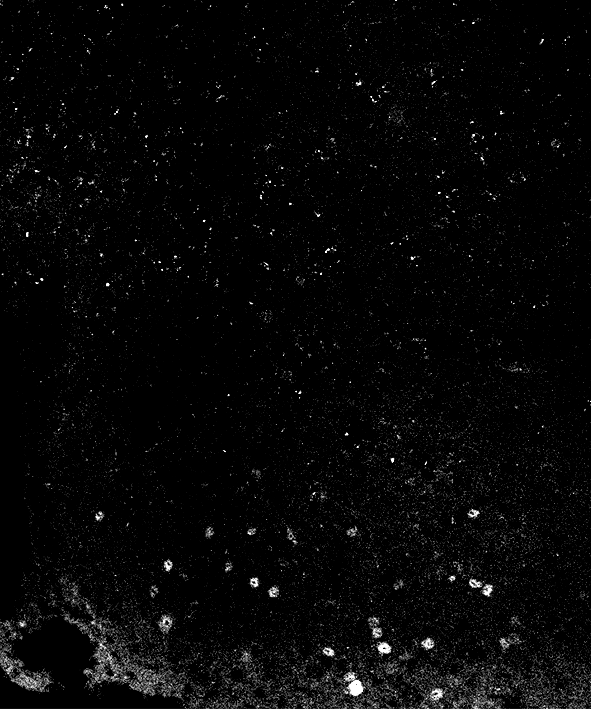

Supplement: Supplementary file 10 — Source Data for Figure 6 [file EMBR-21-e49807-s008.zip › EMBOR-2019-49807V1_Fig6B2_FOS_KO.tif]

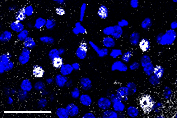

Supplement: Supplementary file 10 — Source Data for Figure 6 [file EMBR-21-e49807-s008.zip › EMBOR-2019-49807V1_Fig6B3_MERGE_KO.tif]

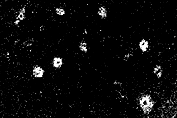

Supplement: Supplementary file 10 — Source Data for Figure 6 [file EMBR-21-e49807-s008.zip › EMBOR-2019-49807V1_Fig6B4_FOS_KO.tif]

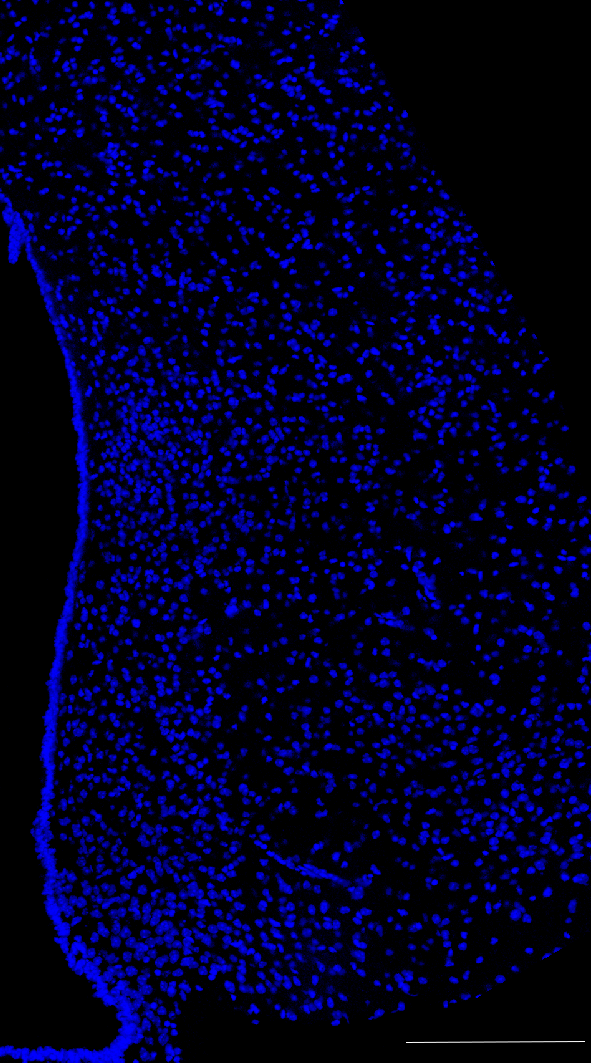

Supplement: Supplementary file 10 — Source Data for Figure 6 [file EMBR-21-e49807-s008.zip › EMBOR-2019-49807V1_Fig6C1_HOECHST_WT.tif]

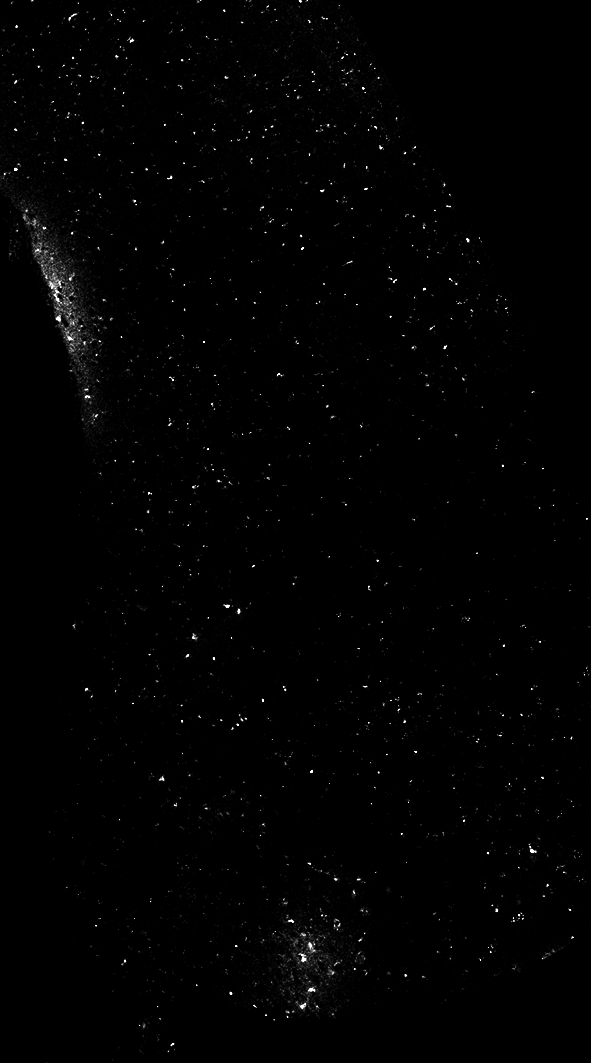

Supplement: Supplementary file 10 — Source Data for Figure 6 [file EMBR-21-e49807-s008.zip › EMBOR-2019-49807V1_Fig6C2_FOS_WT.tif]

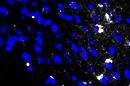

Supplement: Supplementary file 10 — Source Data for Figure 6 [file EMBR-21-e49807-s008.zip › EMBOR-2019-49807V1_Fig6C3_MERGE_WT.tif]

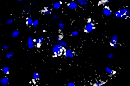

Supplement: Supplementary file 10 — Source Data for Figure 6 [file EMBR-21-e49807-s008.zip › EMBOR-2019-49807V1_Fig6C4_MERGE_WT.tif]

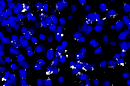

Supplement: Supplementary file 10 — Source Data for Figure 6 [file EMBR-21-e49807-s008.zip › EMBOR-2019-49807V1_Fig6C5_MERGE_WT.tif]
